# Global, regional, and national burden of schizophrenia: epidemiological trends, decomposition, joinpoint analysis, and projections to 2036 based on GBD 2021

**DOI:** 10.3389/fpsyt.2026.1702808

**Published:** 2026-02-12

**Authors:** Wanting Zhou, Jiujie He, Lishuo Wu, Liwen Wang, Kejun Wu, Zongyu Li, Gang Chen, Donglian Li, Yi Yang, Jian Dai

**Affiliations:** 1 Department of Clinical Psychology, Jiangbin Hospital of Guangxi Zhuang Autonomous Region, Nanning, Guangxi, China; 2 Department of Traditional Chinese Medicine, Guangxi Medical University Cancer Hospital, Nanning, Guangxi, China; 3 Department of Pathology, The First Affiliated Hospital of Guangxi Medical University, Nanning, Guangxi, China

**Keywords:** DALYs, decomposition, epidemiological trends, incidence, prevalence, schizophrenia

## Abstract

**Background:**

Schizophrenia continues to impose a substantial burden within the global spectrum of disease. This study utilizes the most recent data from the Global Burden of Disease Study 2021 (GBD 2021) to analyze epidemiological trends specific to schizophrenia and generate empirical evidence to guide the development of targeted prevention and clinical management strategies.

**Methods:**

Leveraging the GBD 2021 dataset, we examined age-standardized incidence, prevalence, and disability-adjusted life years (DALYs) for schizophrenia. We further evaluated temporal trends and age-sex-specific variations; executed decomposition analysis to identify primary determinants; conducted Joinpoint regression analysis; and generated projections through 2036.

**Results:**

In 2021, schizophrenia incidence demonstrated a declining trend, whereas prevalence and DALYs exhibited increasing trends. The burden disproportionately affected younger and middle-aged males, with a concomitant progressive increase observed among females. Significant disparities existed across geographic regions, high-SDI regions exhibited persistently elevated burden with upward trajectories, whereas low-SDI regions demonstrated progressive increases from lower baseline levels. These patterns were primarily attributable to population growth, SDI levels, and epidemiological parameter shifts, while population aging attenuated the schizophrenia burden.

**Conclusions:**

While schizophrenia currently exerts a disproportionately higher burden on young and middle-aged males, our projections indicate a notable epidemiological shift, with females expected to experience a steeper rise in incidence, prevalence, and DALYs over the next 15 years. This emerging female-centric trend contrasts sharply with the current male-dominated profile, underscoring the urgent need for sex-responsive mental health strategies.

## 1 Introduction

Schizophrenia is a severe, chronic psychiatric disorder characterized by disturbances in thought, perception, affect and behavior that profoundly impair cognitive, social and occupational functioning (1). With a lifetime prevalence of approximately 0.3% worldwide, onset typically occurs in early adulthood and follows a chronic, often lifelong course (2). Schizophrenia is associated with numerous adverse outcomes—including unemployment (3), homelessness (4), substance use disorders (5), non-suicidal self-injury (NSSI) and suicidal behaviors (6, 7) and confers a two- to three-fold increase in all-cause mortality (8–10), collectively imposing substantial socioeconomic burdens on individuals, families and health systems. Accurate, ongoing quantification of disease burden is therefore essential for designing evidence-based preventive interventions, optimizing mental-health resource allocation and formulating targeted public-health policies across diverse settings.

While previous studies have provided useful cross-sectional snapshots of schizophrenia burden (11–16), they have not yet fully exploited the predictive value of longitudinal, sex-specific data; have rarely translated age-stratified findings into actionable intervention targets; have often grouped heterogeneous psychiatric conditions under a single umbrella; and have tended to offer broad recommendations without socio-demographic stratification. These gaps motivate the present, more granular and forward-looking analysis. Up-to-date, integrated epidemiological intelligence remains scarce, impeding evaluation of how COVID-19, armed conflicts and economic crises have disrupted mental-health services and exacerbated social isolation. Furthermore, underutilization of Joinpoint regression, decomposition analysis and ARIMA modelling has hindered elucidation of temporal dynamics and their underlying drivers.

To address these gaps, we analyzed GBD 2021 estimates of schizophrenia prevalence, incidence and DALYs for 204 countries and territories. Joinpoint regression was employed to detect significant trend inflections, and Das Gupta decomposition to attribute temporal changes in incidence, prevalence and DALYs to demographic versus epidemiological drivers. We provide the sex-stratified ARIMA projections of global schizophrenia burden to 2036. Together, these analyses generate robust evidence to inform resource allocation and equity-oriented, context-specific prevention strategies.

## 2 Methods

### 2.1 Data source

The GBD 2021 provides comprehensive epidemiological estimates for 204 countries and territories, which are grouped into five Socio-demographic Index (SDI) quintiles and 21 GBD regions, covering 371 diseases and injuries as well as 88 risk factors over the period 1990–2021 (17). This article analyses the incidence, prevalence, and DALYs attributable to schizophrenia in 204 countries and territories for 1990–2021. All data were extracted from the publicly available GBD 2021 repository (https://vizhub.healthdata.org/gbd-results/). We systematically extracted country- and territory-specific estimates of incidence, prevalence, and DALY counts for schizophrenia from the same source. Specifically, we obtained incident cases, prevalent cases, DALY counts, age-standardized incidence rates (ASIR), age-standardized DALY rates (ASDR), and their 95% uncertainty intervals (UIs). All estimates were generated in accordance with the Guidelines for Accurate and Transparent Health Estimates Reporting (GATHER) (18), thereby guaranteeing methodological transparency and reproducibility.

The Social Demographic Index (SDI), a composite indicator introduced in 2015 by the Institute for Health Metrics and Evaluation (IHME), summarizes the developmental level of countries and territories and is used to explore how social development correlates with population health outcomes (17). Its calculation combines three dimensions: average lag-distributed income per capita, mean years of schooling for adults aged ≥15 years, and the total fertility rate (19). SDI takes values between 0 and 1, with higher scores indicating greater socioeconomic development. The 204 countries and regions were grouped into five development strata: low, low-middle, middle, high-middle, and high.

### 2.2 Advanced statistical analysis

#### 2.2.1 Decomposition analysis

Decomposition analysis (DA) applies Das Gupta’s method for partitioning changes in the schizophrenia burden into three components: population ageing, population growth, and shifts in disease prevalence, thereby quantifying each factor’s contribution to the net change (20). When this method is used to examine schizophrenia trends, it allows us to estimate the relative contribution of demographic versus epidemiological drivers and to infer the potential impact of public-health interventions. Consequently, DA pinpoints specific areas where public-health action is most needed, facilitating the design of targeted strategies. Drawing upon the research methodology of Bai Zihao et al. (21), the specific calculation formula is detailed in the **Supplementary Materials**.

#### 2.2.2 Joinpoint regression analysis

We used Joinpoint regression (version 4.9.1.0; NCI, USA) to characterize piece-wise linear trends in age-standardized incidence, prevalence and DALY rates of schizophrenia. The algorithm sequentially tests H_0_: no joinpoint versus H_1_ k joinpoints (k ≤ 5), with each additional inflection point retained only if the Monte-Carlo permutation p < 0.05. Model selection follows a hierarchical procedure in which the simplest model with the smallest Bayesian Information Criterion (BIC) is preferred. Segment-specific annual percentage change (APC) and overall average annual percentage change (AAPC), together with their 95% confidence intervals (CIs), were the primary metrics (22, 23). APC estimates the yearly rate of change within each segment, whereas AAPC provides a weighted summary of these APCs across the whole study period. A trend was deemed statistically significant if the 95% CI did not cross zero; the direction of change was inferred from the sign of the point estimate. The specific calculation formula is detailed in the **Supplementary Materials**, referencing the research methodology of Gillis D et al. (24).

#### 2.2.3 Autoregressive integrated moving average model analysis

The ARIMA model—a benchmark approach for time-series forecasting—was employed to predict global incidence, prevalence and DALYs from schizophrenia over the period 2022–2036. After differencing to achieve stationarity, the model accounts for both autoregressive and moving-average components, thereby capturing long-term trends and short-term fluctuations (25, 26). The optimal order (p, d, q) was identified using the auto. Arima () function in R, which minimizes the Akaike Information Criterion (AIC); residual white noise was subsequently verified with the Ljung-Box test (p > 0.05) (26, 27). The specific calculation formula is detailed in the **Supplementary Materials**, referencing the research methodology of KE Arunkumar et al. (28).

All statistical analyses and data visualizations were implemented using R software (version 4.3.3). Descriptive statistics for key variables were computed and reported as means accompanied by 95% uncertainty intervals (UIs) or 95% confidence intervals (CIs), as appropriate to each metric. Statistical significance in trend analyses was defined *a priori* as P<0.05.

## 3 Results

### 3.1 Global burden of schizophrenia

**Figure 1:**
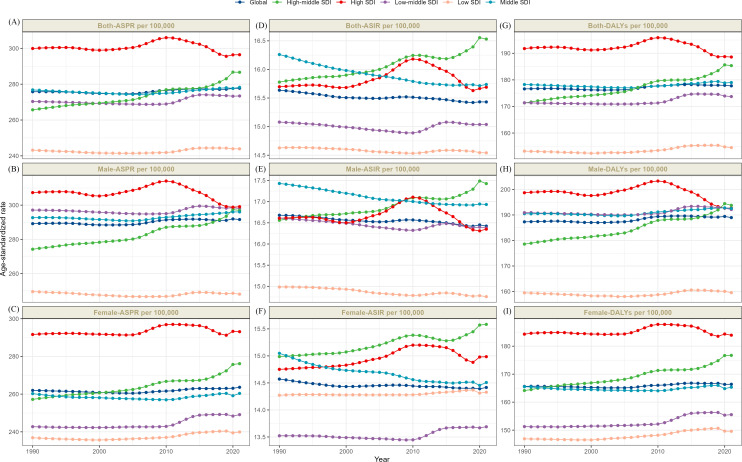
The trends of ASRs o both-ASPR **(A)**, male-ASPR **(B)**, female-ASPR **(C)**, both-ASIR **(D)**, male-ASIR **(E)**, female-ASIR **(F)** and both-DALYs **(G)**, male- DALYs **(H)**, female- DALYs **(I)** of schizophrenia by global and 5 SDI regions, 1990-2021.

**Figure 2:**
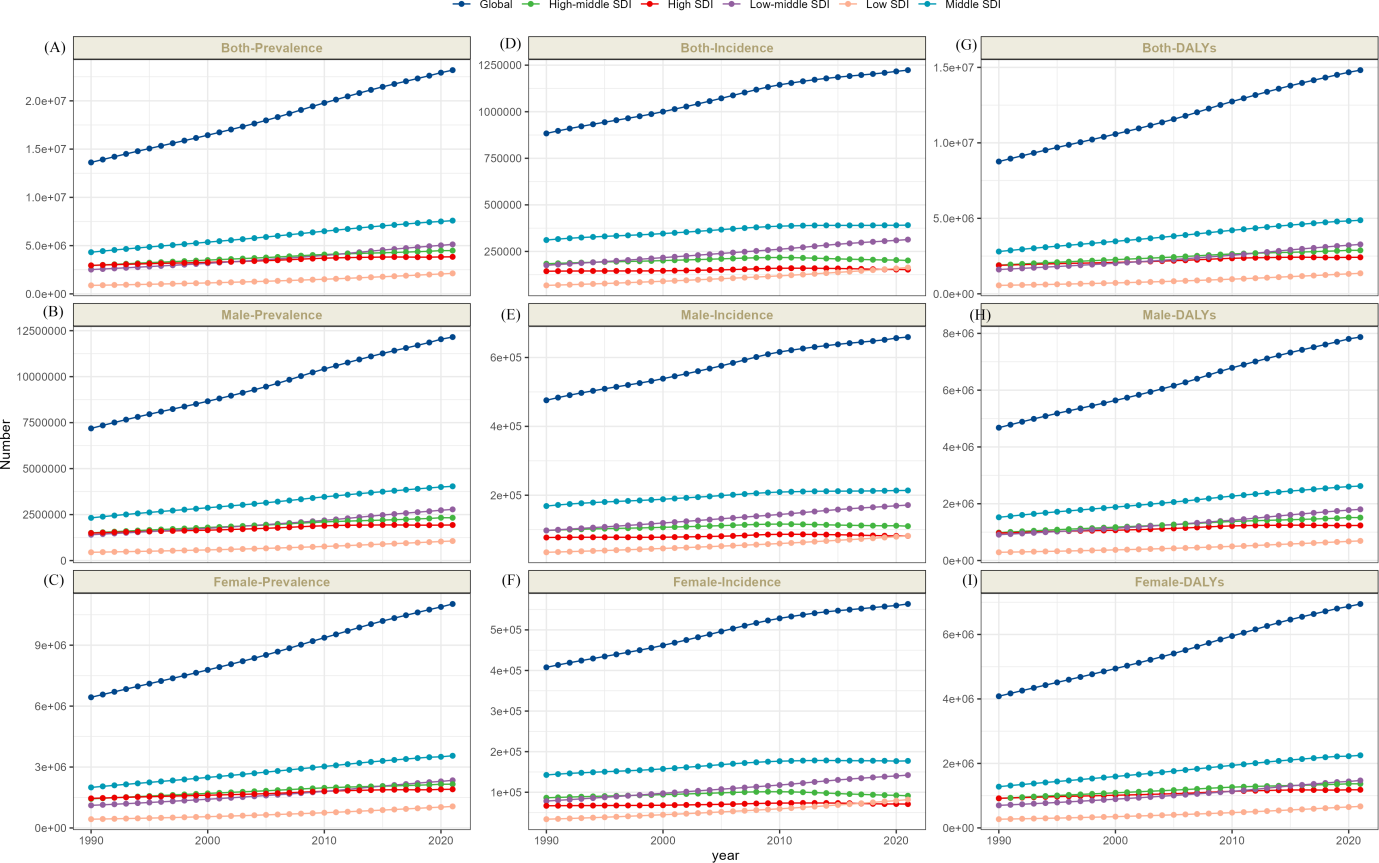
The trends of cases number of both-prevalence **(A)**, male- prevalence **(B)**, female- prevalence **(C)**, both-incidence **(D)**, male- incidence **(E)**, female- incidence **(F)** and both-DALYs **(G)**, male- DALYs **(H)**, female- DALYs **(I)** of schizophrenia by global and 5 SDI regions, 1990-2021.

In 2021, globally, incidence schizophrenia cases totaled 1223221 (95% UI: 1008219 - 1473083), corresponding to an age-standardized incidence rate (ASIR) of 15.43 per 100,000 population (95% UI: 12.74 - 18.62), representing a 0.04% decline versus 1990 (EAPC −0.04, 95% CI: −0.04 to −0.03). Prevalent cases reached 23182109 globally (95% UI: 19203759 -27,423,876), with an age-standardized prevalence rate (ASPR) of 277.71 per 100,000 (95% UI: 229.77–329.06), marking a 0.03% increase from 1990 (EAPC 0.03, 95% CI: 0.02 to 0.04). Schizophrenia accounted for 1486611 DALYs (95% UI: 1092646 - 909536), yielding an age-standardized DALYs rate (ASDR) of 177.75 per 100,000 (95% UI: 131.51–228.80), a 0.04% increase versus 1990 (EAPC 0.04, 95% CI: 0.03 to 0.05). Collectively, these patterns indicate that prevalence and DALYs constitute the primary drivers of schizophrenia’s growing disease burden (**Supplementary Tables S1**-**S3**; **Figures 1**, **2**). Additionally, the annual percentage changes in the incidence, prevalence, and DALYs of schizophrenia worldwide are detailed in **Table 1**.

**Table 1:** Average annual percent change in incidence, prevalence, and DALYs of schizophrenia by SDI and sex.

Characteristics	Global	High SDI	High-middle SDI	Middle SDI	Low-middle SDI	Low SDI
Incidence						
Both	-0.04 (-0.05 - -0.04)	-0.00 (-0.01 - 0.01)	0.16 (0.13 - 0.18)	-0.11 (-0.11 - -0.10)	-0.01 (-0.01 - -0.01)	-0.02 (-0.03 - -0.01)
Female	-0.04 (-0.04 - -0.03)	0.06 (0.05 - 0.06)	0.13 (0.11 - 0.14)	-0.12 (-0.14 - -0.11)	0.04 (0.03 - 0.04)	0.01 (0.00 - 0.02)
Male	-0.05 (-0.06 - -0.04)	-0.05 (-0.08 - -0.02)	0.17 (0.14 - 0.20)	-0.09 (-0.10 - -0.08)	-0.04 (-0.05 - -0.04)	-0.05 (-0.05 - -0.04)
Prevalence						
Both	0.02 (0.02 - 0.03)	-0.04 (-0.05 - -0.03)	0.25 (0.22 - 0.28)	0.02 (0.01 - 0.02)	0.03 (0.03 - 0.04)	0.01 (0.00 - 0.02)
Female	0.02 (0.01 - 0.03)	0.02 (0.01 - 0.03)	0.23 (0.21 - 0.25)	-0.00 (-0.02 - 0.01)	0.09 (0.07 - 0.10)	0.04 (0.02 - 0.06)
Male	0.03 (0.01 - 0.05)	-0.09 (-0.10 - -0.08)	0.26 (0.23 - 0.29)	0.04 (0.03 - 0.04)	0.01 (0.00 - 0.02)	-0.02 (-0.03 - -0.01)
DALYs						
Both	0.02 (0.01 - 0.03)	-0.05 (-0.07 - -0.04)	0.26 (0.23 - 0.29)	0.01 (0.01 - 0.02)	0.04 (0.04 - 0.05)	0.03 (0.02 - 0.03)
Female	0.01 (0.01 - 0.02)	-0.00 (-0.02 - 0.01)	0.24 (0.21 - 0.27)	-0.01 (-0.03 - 0.01)	0.09 (0.06 - 0.11)	0.05 (0.05 - 0.06)
Male	0.03 (0.01 - 0.05)	-0.10 (-0.12 - -0.09)	0.27 (0.23 - 0.31)	0.04 (0.03 - 0.04)	0.02 (0.01 - 0.03)	0.00 (-0.01 - 0.02)

### 3.2 Regional burden of schizophrenia

In 2021, among the five SDI regions, low SDI regions had the lowest age-standardized incidence rate of Schizophrenia (14.54 per 100,000 [95% UI: 11.76–17.87]), the prevalence rate (243.92 per 100,000 [95% UI: 197.83–297.13]), and DALYs (154.51 per 100,000 [95% UI: 112.40–202.64]). Conversely, high SDI regions demonstrated the highest ASPR (296.45 per 100,000; 95% CI: 247.61–349.30) and ASDR 188.61 per 100,000; 95% CI: 140.84–241.79). The highest ASIR occurred in high-middle SDI regions (16.53 per 100,000; 95% UI: 14.15–19.33). Longitudinal analysis revealed divergent trends: prevalence rates and DALYs showed no significant change in high SDI areas, while the remaining four SDI tiers exhibited upward trajectories, indicating a worsening disease burden across these four SDI levels. Similarly, incidence rates increased in high and high-middle SDI regions but decreased in low, low-middle, and middle SDI regions (**Supplementary Tables S1**-**S3**; **Figures 1**, **2**). Additionally, the annual percentage changes in incidence, prevalence, and DALYs for schizophrenia in the 5SDI regions are detailed in **Table 1**.

**Figure 3:**
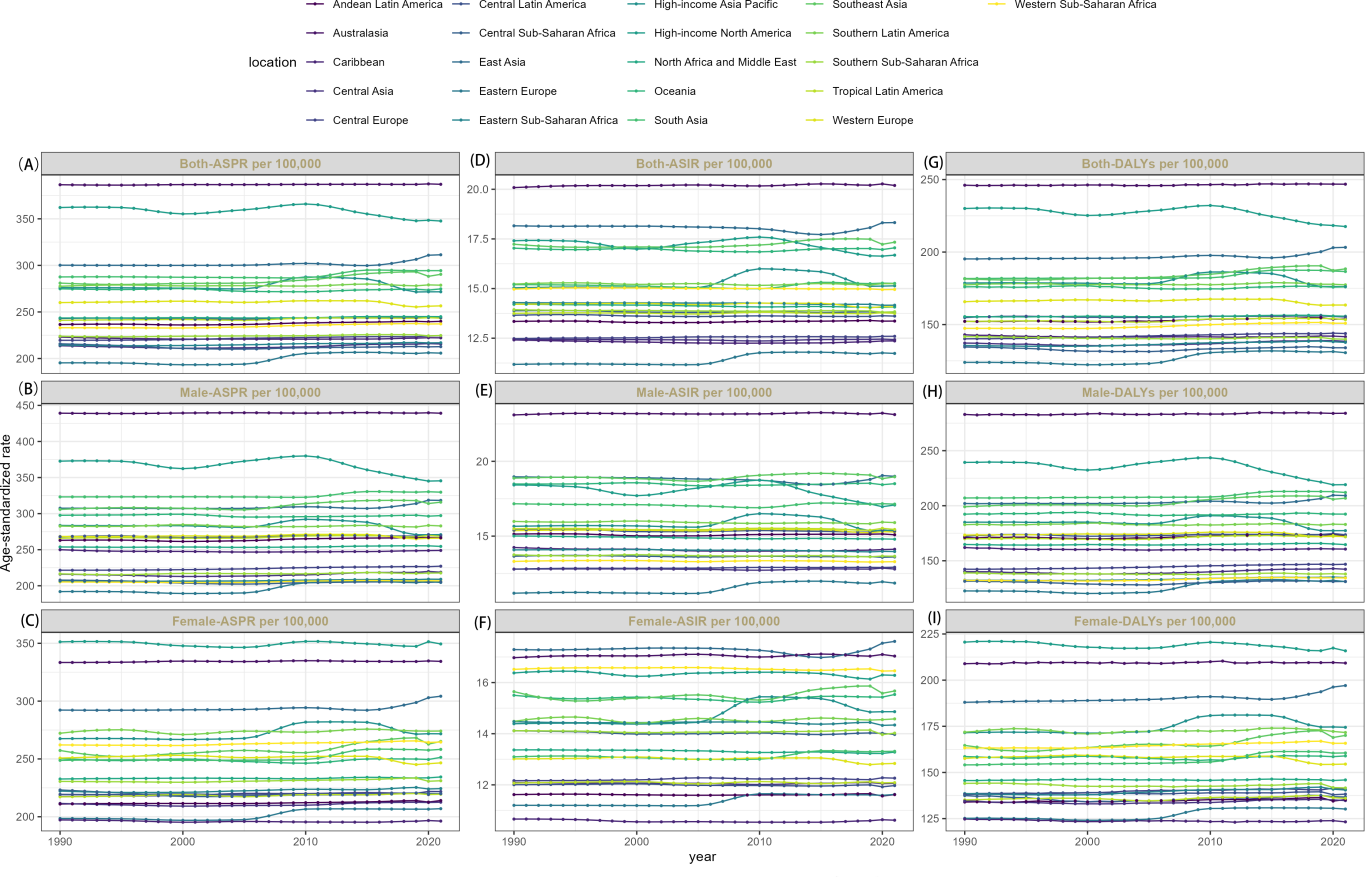
The trends of ASRs of both-ASPR **(A)**, male-ASPR **(B)**, female-ASPR **(C)**, both-ASIR **(D)**, male-ASIR **(E)**, female-ASIR **(F)** and both-DALYs **(G)**, male- DALYs **(H)**, female- DALYs **(I)** of schizophrenia by 21 GBD regions, 1990-2021.

**Figure 4:**
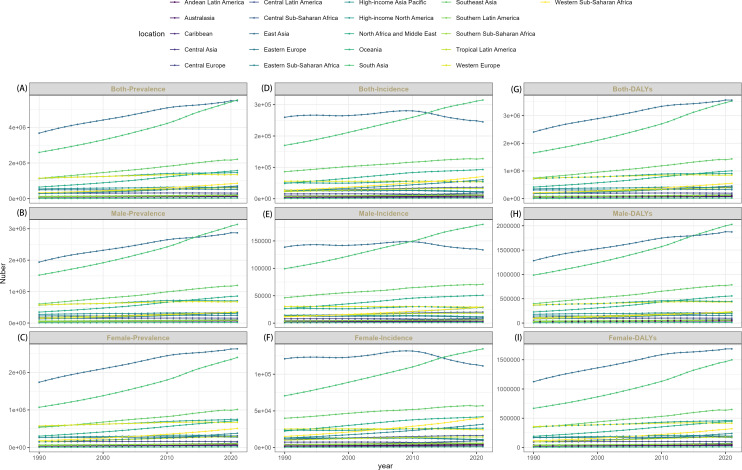
The trends of cases number of both-prevalence **(A)**, male- prevalence **(B)**, female- prevalence **(C)**, both-incidence **(D)**, male- incidence **(E)**, female- incidence **(F)** and both-DALYs **(G)**, male- DALYs **(H)**, female- DALYs **(I)** of schizophrenia by 21 GBD regions, 1990-2021.

In 2021, across the 21 GBD regions, Eastern Europe exhibited the lowest age-standardized incidence rate (11.73 per 100,000; 95% UI: 9.65-14.21), prevalence rate (205.80 per 100,000; 95% UI: 169.71-245.81), and DALYs rate (130.60 per 100,000; 95% UI: 96.54-169.32). Conversely, Australasia demonstrated the highest incidence rate (20.19 per 100,000; 95% UI: 17.87-22.74), prevalence rate (387.08 per 100,000; 95% UI: 355.39-420.53), and DALYs rate (246.81 per 100,000; 95% UI: 186.31-300.92). Longitudinal analysis of EAPC revealed incidence rates exhibited stable or increasing trends in 7 regions, while prevalence rates demonstrated stable or increasing trends in 16 regions, and DALYs rates demonstrated increasing trends in 16 regions. In the remaining regions, schizophrenia burden declined, with the most substantial reductions in incidence, prevalence, and DALYs occurring in High-income North America (**Supplementary Tables S1**–**S3**; **Figures 3**, **4**).

### 3.3 National burden of schizophrenia

Significant cross-national variations emerged in schizophrenia’s age-standardized burden metrics across 204 countries and territories in 2021.

**Figure 5:**
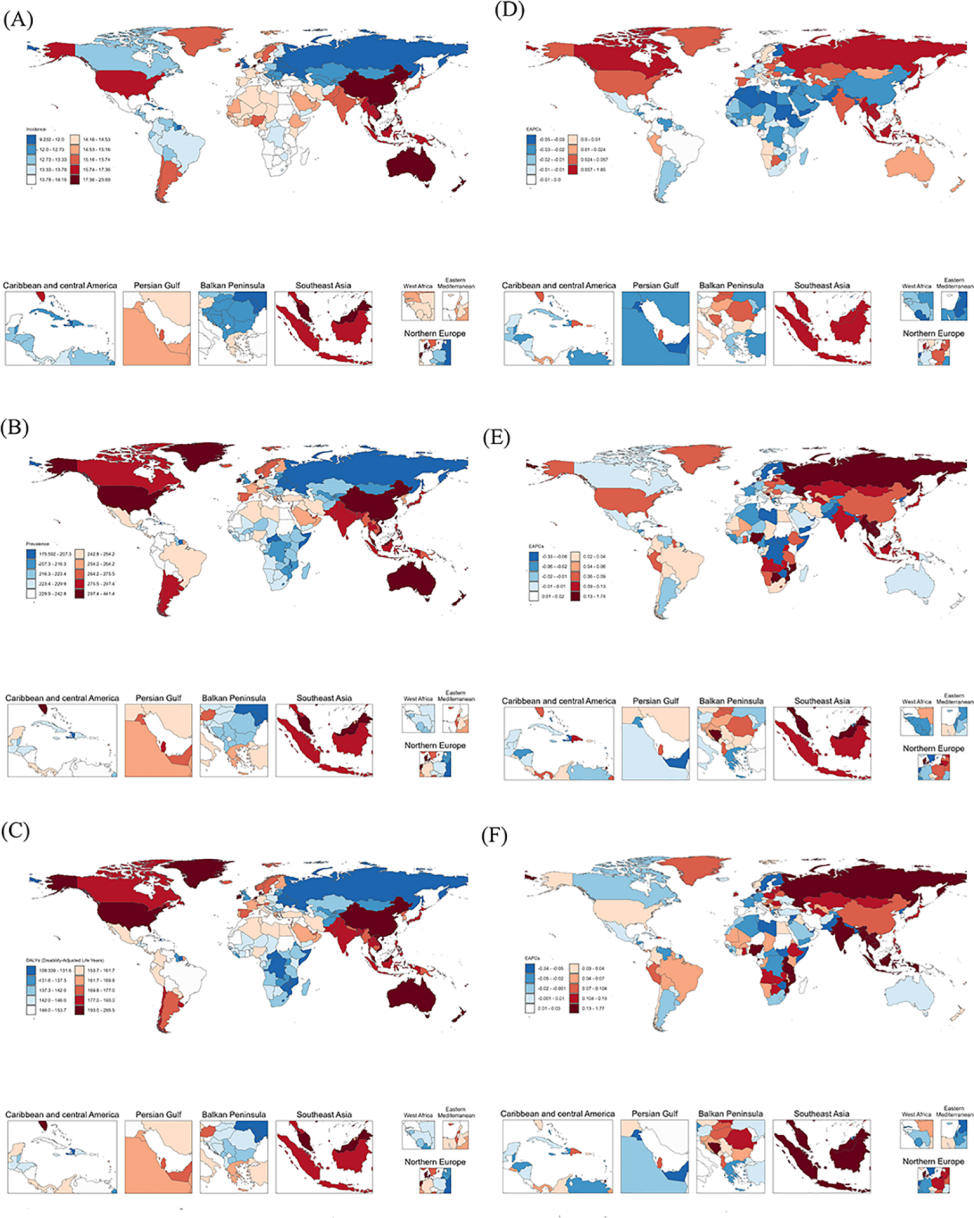
Global burden of ASMR **(A)**, ASPR **(B)**, ASDR **(C)**, EAPC of ASMR **(D)**, EAPC of ASPR **(E)** and EAPC of ASDR **(F)** of schizophrenia in 2021.

Suriname exhibited the lowest incidence rate globally (10.77 per 100,000; 95% UI: 9.37–12.46), while Somalia demonstrated both the lowest prevalence rate (196.06 per 100,000; 95% UI: 152.11–248.66) and lowest DALYs rate (124.77 per 100,000; 95% UI: 88.99–168.39). Conversely, Denmark recorded the highest incidence rate (21.28 per 100,000; 95% UI: 19.39–22.28), whereas Australia showed peak prevalence rate (388.26 per 100,000; 95% UI: 358.09–419.41) and DALYs burden (247.58 per 100,000; 95% UI: 186.20–300.20) (**Figures 5A–C**).

Analysis of EAPCs revealed schizophrenia incidence remained stable or exhibited gradual increases in 84 countries, with Denmark demonstrating the most pronounced increase. Conversely, 120 countries experienced declining trends, with the United Kingdom showed the most substantial reduction (**Figure 5D**). Regarding prevalence, 74 countries exhibited declining trends, with the United Kingdom experiencing the greatest decrease. In contrast, 130 countries exhibited stability or increasing trends, with Denmark demonstrating the most significant increase (**Figure 5E**). For DALYs, 131 countries showed increasing trends, with Denmark demonstrating the highest increase; 7 countries remained stable, while 66 experienced reductions, with the United Kingdom exhibiting the most substantial decline (**Figure 5F**). The detailed data on the schizophrenia burden across 204 countries and regions are presented in **Supplementary Tables S4**-**S6**.

### 3.4 Age-sex-time trends in schizophrenia disease burden

**Figure 6:**
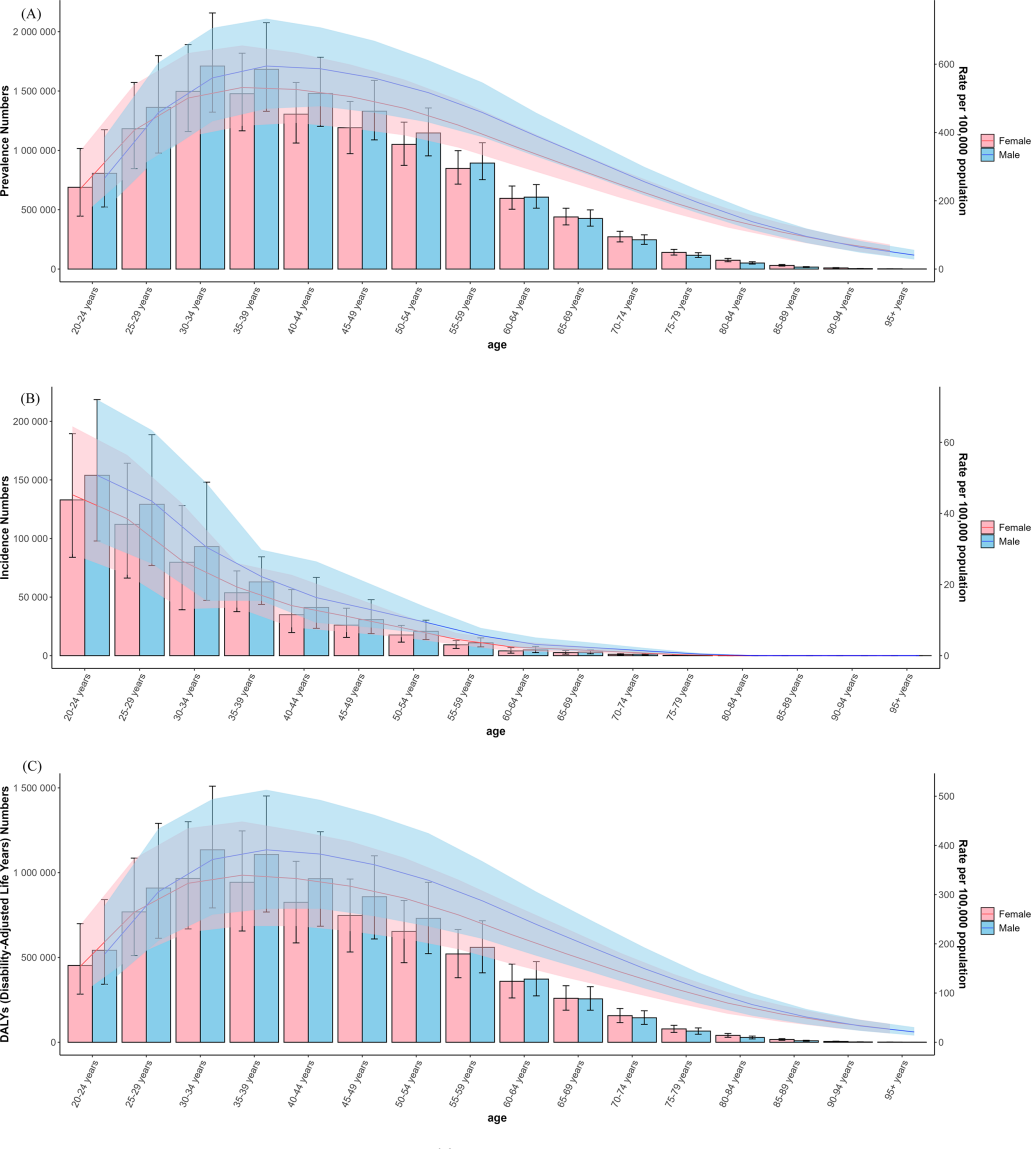
The global of age-gender trends of prevalence **(A)**, incidence **(B)** and DALYs **(C)** in schizophrenia burden.

Global analyses indicated higher schizophrenia prevalence, incidence, and DALY rates among males than females (**Figures 6A–C**). The most pronounced sex-based disparities occurred in younger and middle-aged populations: before age 25, males exhibited lower prevalence and DALYs than females, though incidence was significantly higher in males from age 20 onward. In 2021, male prevalence and DALY rates peaked at ages 35–39 years. Absolute prevalent cases and DALYs reached maxima in the 30–34 age group, declining progressively through older age strata. Incidence demonstrated a sustained downward trajectory from age 20 through the oldest cohorts.

### 3.5 Relationship between schizophrenia burden and SDI

**Figure 7:**
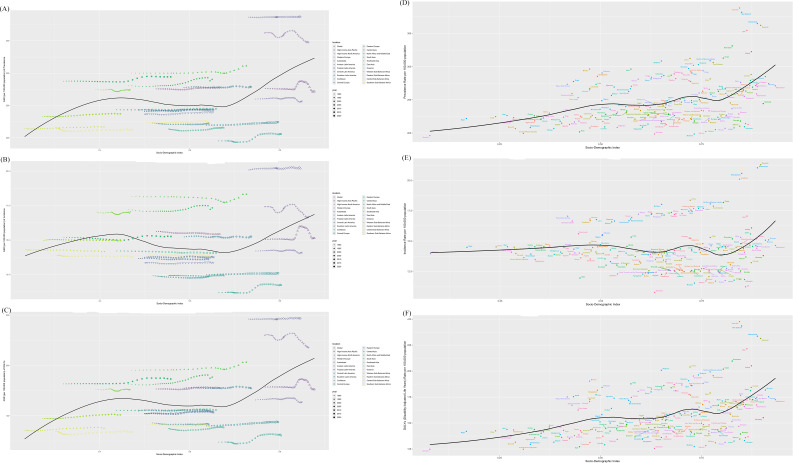
The SDI analysis results of prevalence across 21 regions **(A)**, incidence across 21 regions **(B)**, DALYs across 21 regions **(C)**, prevalence across 204 regions **(D)**, incidence across 204 regions **(E)** and DALYs across 204 regions **(F)**.

Globally and across all 21 GBD regions, schizophrenia’s age-standardized burden metrics (incidence, prevalence, DALYs) exhibited a U-shaped association with the SDI. This pattern indicates that the burden was lowest within the mid-SDI range of 0.45–0.65 and increased significantly at SDI levels below 0.45 and above 0.65. Notably, Australasia, Western Europe, and East Asia experienced a substantial escalation in burden, which is evident from the steeper slopes of the curves in these regions (**Figures 7A–C**), whereas other regions showed more stable or moderately increasing trajectories. In 204 countries, a nonlinear association was identified between the Socio-Demographic Index (SDI) and the age-standardized incidence rates, prevalence rates, and Disability-Adjusted Life Years (DALYs) for schizophrenia in 2021, as depicted by the S-shaped curves in **Figures 7D–F**). The disease burden initially increases with SDI, reaching a plateau around SDI 0.5, and then escalates again beyond SDI 0.76, indicating a complex relationship where the burden is not consistently increasing with SDI.

### 3.6 Results of the decomposition analysis for schizophrenia

**Figure 8:**
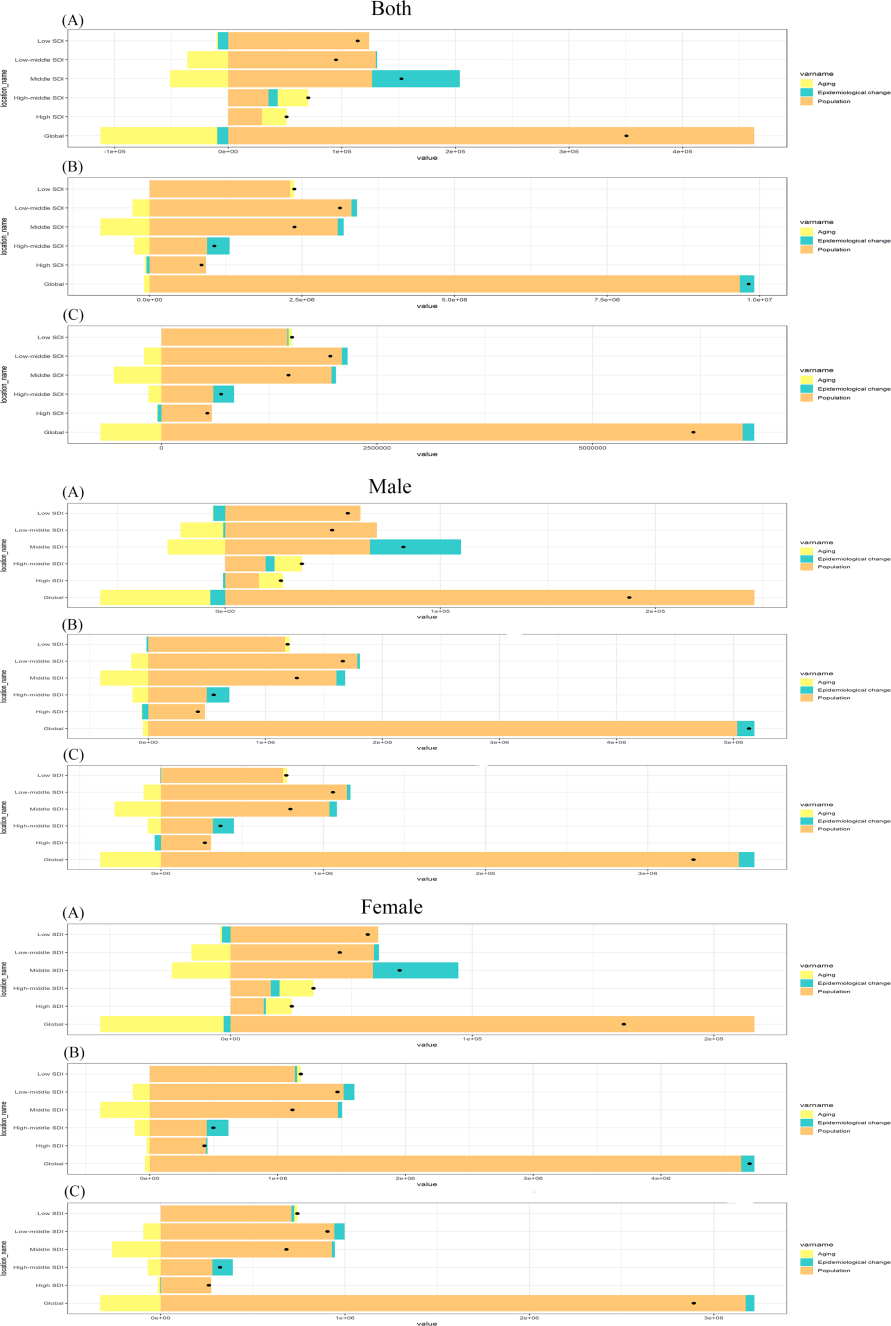
Decomposition analysis results of incidence **(A)**, prevalence **(B)** and DALYs **(C)** by the global and 5 SDI regions.

Globally, aging, population, and epidemiological changes have led to changes in schizophrenia incidence rates of -29.36%, 132.12%, and -2.76%; prevalence rates of -0.94%, 98.53%, and 2.4%; and DALYs of -11.48%, 109.31%, and 2.17%. Population growth consistently increased burden across all Socio-demographic Index (SDI) regions. Regional analysis determined that population growth is the main driver of the increased burden of schizophrenia in both men and women, followed by epidemiological changes, while aging in some SDI regions helps alleviate the burden of schizophrenia (**Figure 8**; **Supplementary Tables S7**). 21 regions detailed results of the regional decomposition analysis are provided in **Supplementary Tables S8**.

### 3.7 Joinpoint regression analysis for schizophrenia

**Figure 9:**
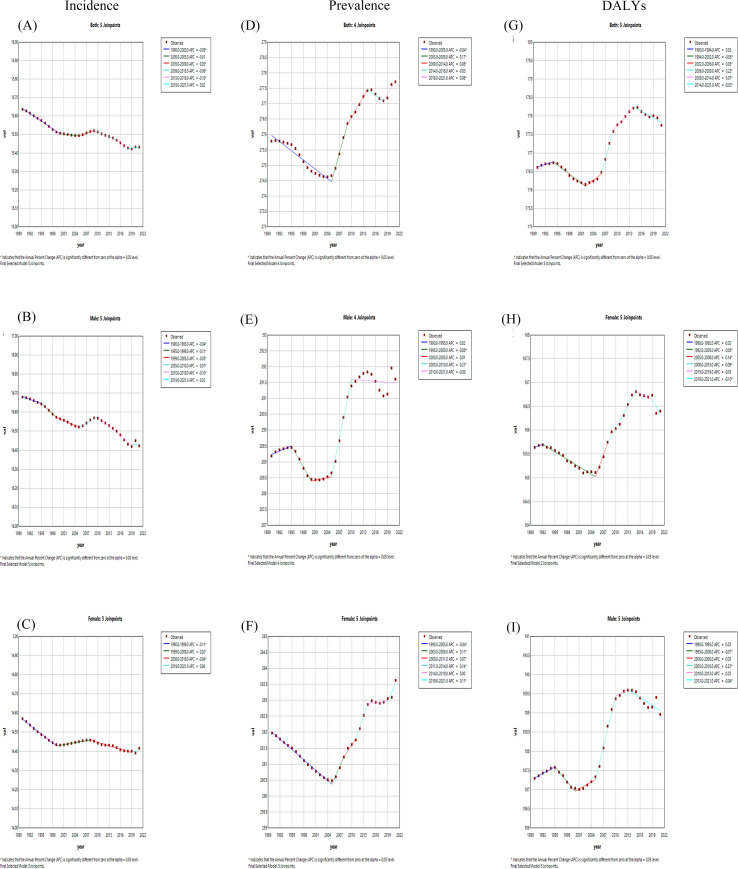
Joinpoint regression analysis of both-incidence **(A)**, male-incidence **(B)**, female-incidence **(C)**, both-prevalence **(D)**, male- prevalence **(E)**, female- prevalence **(F)** and both-DALYs **(G)**, male- DALYs **(H)**, female- DALYs **(I)** of age-standardized rate for schizophrenia in global from 1990-2021.

Joinpoint regression analysis revealed globally declining schizophrenia incidence trends for both sexes (**Figures 9A–C**), while prevalence and DALYs exhibited initial declines followed by subsequent increases (**Figures 9D, I**). For prevalence, both sexes combined and female demonstrated a sharp decline (1990–2005) followed by an upward trajectory (2005–2021). Male displayed an initial slow increase (1990–2005), subsequent decline with stabilization, and ultimately an upward trend (2005–2021), culminating in an overall prevalence increase (**Figures 9D–F**). DALY trends were consistent across sex stratifications: slow initial increase (1990–2005), rapid decline, then accelerated growth peaking circa 2014 before subsequent reduction (**Figures 9G–I**)). Detailed results of DALYs node analysis and APC are presented in **Table 2**. Additionally, detailed results of Incidence and Prevalence node analysis and APC are provided in **Supplementary Tables S9**–**S10**.

**Table 2:** Joinpoint Analysis and APC in DALYs for schizophrenia by the SDI and Sex.

Characteristics	Sex	K_Joinpoints	Joinpoints	APC
Global	Both	6	1990, 1994, 2002, 2006, 2009, 2014	0.02, -0.05,0.05,0.22,0.07, -0.03
	Female	6	1990, 1992, 2005, 2008, 2015, 2019	0.02, -0.03,0.14,0.09, -0.03, -0.01
	Male	6	1990, 1995, 2000, 2005, 2010, 2013	0.03, -0.07,0.03,0.23,0.03, -0.04
SDI				
High SDI	Both	7	1990, 1995, 2000, 2005, 2010, 2015, 2019	0.06, -0.14,0.12,0.41, -0.28, -0.64,0.03
	Female	6	1990, 1993, 2005, 2010, 2015, 2019	0.1, -0.03,0.41, -0.08, -0.5,0.16
	Male	7	1990, 1995, 2000, 2005, 2010, 2015, 2019	0.06, -0.2,0.24,0.4, -0.46, -0.78, -0.09
High-middle SDI	Both	4	1990, 2005, 2010, 2016	0.15,0.47,0.06,0.61
	Female	4	1990, 2005, 2010, 2016	0.15,0.38,0.04,0.61
	Male	4	1990, 2005, 2010, 2016	0.15,0.55,0.06,0.6
Middle	Both	3	1990, 2006, 2019	-0.05,0.11, -0.14
	Female	4	1990, 1996, 2010, 2018	-0.09, -0.03, 0.15, -0.17
	Male	5	1990, 1994, 2005, 2010, 2019	0.01, -0.05, 0.17, 0.09, -0.03
Low-middle	Both	5	1990, 2004, 2010, 2015, 2019	-0.02,0.04,0.41, -0.03, -0.25
	Female	5	1990, 1995, 2010, 2015, 2018	-0.02, 0.04, 0.53, 0.06, -0.21
	Male	5	1990, 2006, 2011, 2014, 2019	-0.03, 0.09, 0.44, -0.02, -0.24
Low middle	Both	6	1990, 2000, 2006, 2011, 2015, 2019	-0.05, 0.03, 0.13, 0.27, 0, -0.29
	Female	5	1990, 2000, 2011, 2014, 2019	-0.03, 0.12, 0.34, 0.1, -0.4
	Male	5	1990, 2005, 2010, 2015, 2019	-0.06, 0.09, 0.25, -0.06, -0.2

APC, annual percent change; SDI, sociodemographic index

### 3.8 The ARIMA forecasts for schizophrenia

**Figure 10:**
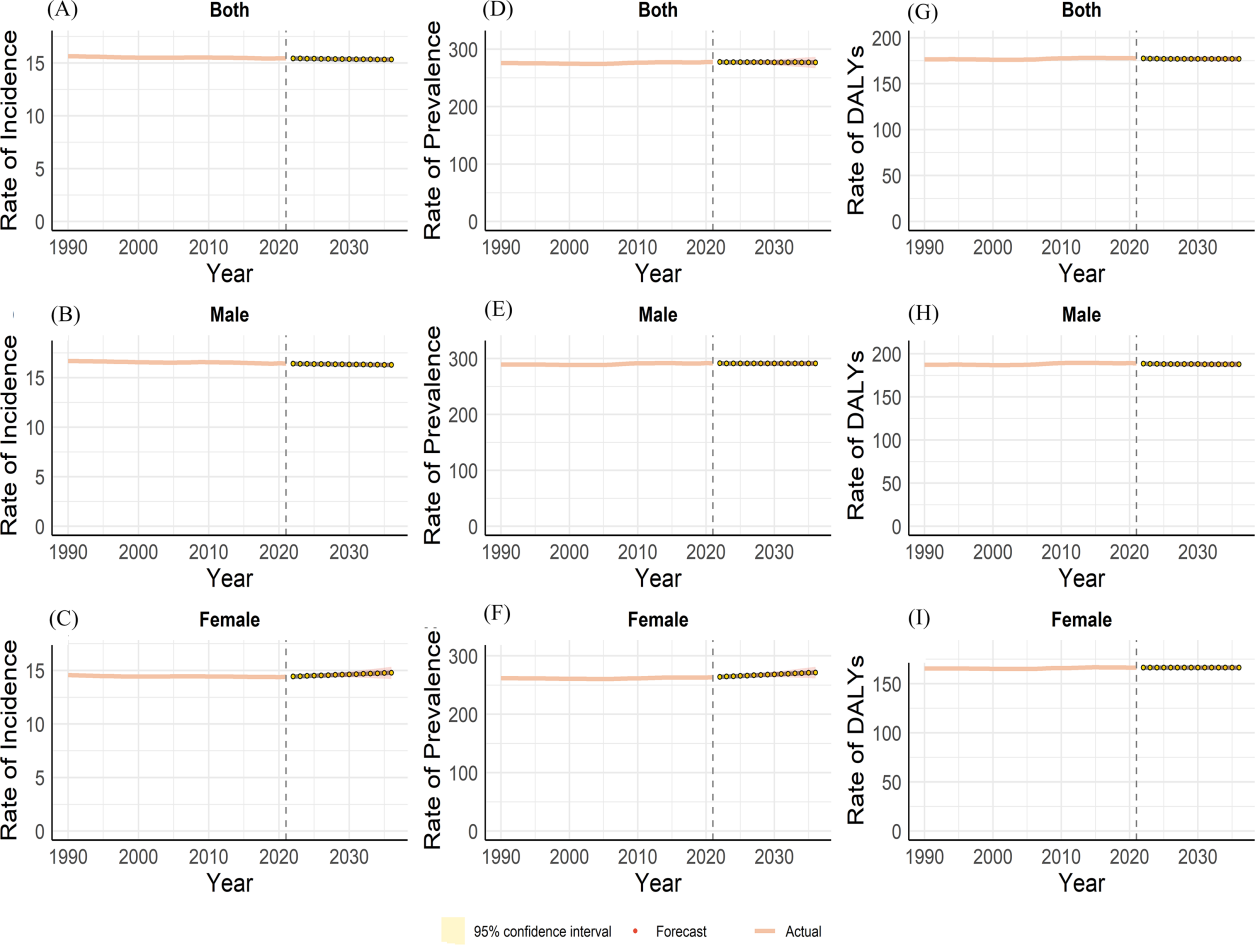
Predictions of both-incidence **(A)**, male-incidence **(B)**, female-incidence **(C)**, both-prevalence **(D)**, male- prevalence **(E)**, female- prevalence **(F)** and both-DALYs **(G)**, male- DALYs **(H)**, female- DALYs **(I)** for schizophrenia from 1990 to 2036 by global based on ARIMA.

ARIMA model predictions (2022 – 2036) indicate stable, non-significant declining trends in schizophrenia incidence and prevalence for both overall and male populations. Conversely, females exhibit significant increasing trajectories across incidence, prevalence, and DALY rates, indicating that schizophrenia will have a greater impact on women’s health over the next 15 years. Therefore, over the next 15 years, there should be an appropriate increase in attention to mental illnesses among women, with proactive measures for prevention and treatment to alleviate the burden on female patients with this disease (**Figure 10A–I**).

## 4 Discussion

This study used the GBD 2021 to comprehensively analyses schizophrenia epidemiology by estimating incidence, prevalence, and DALYs. Das Gupta decomposition was applied to quantify the contributions of population growth, ageing, epidemiological change, and SDI to the overall burden. Stratified analyses by SDI quintile, sex, and age group were conducted to identify context-specific intervention priorities. Finally, ARIMA models forecasted the burden to 2036, providing evidence-based projections for health policy.

Our findings align with and extend several recent studies on the burden of schizophrenia based on GBD data. Consistent with reports by Solmi et al. (9)and Wang et al. (15), we observed generally stable or slightly increasing age-standardized rates globally and across SDI groups. By integrating decomposition analysis, joinpoint regression, and ARIMA modeling, our analytical framework not only describes trends in disease burden but also quantifies the contributions of population growth, aging, and epidemiological shifts, while providing sex-stratified long-term projections. Through this approach, we identified a persistent U-shaped association between SDI and disease burden—a pattern not fully examined previously—and further suggest a potential gradual rise in incidence, prevalence, and disability-adjusted life years among females over the next 15 years. These results corroborate projections by Zhou et al. (29) and Yu & Sun (30) and imply a possible shift in the historically male-dominated burden profile, underscoring the need for gender-sensitive and development-stratified mental health strategies.

The apparent paradox of a marginal decrease in age-standardized incidence concurrent with an increasing absolute burden reflects a balance between the expansive effect of population growth and the protective effect of population ageing, with the working-age group (20–54 years) bearing the largest share of incident cases. In 2021, schizophrenia accounted for an estimated 2.32 (95% UI 2.12–2.53) million prevalent cases, 1.22 (95% UI 1.10–1.35) million incident cases and 1.49 (95% UI 1.34–1.65) million DALYs worldwide. This burden was concentrated among adults aged 20–54 years, resulting in considerable productivity losses and increased healthcare expenditure. Between 1990 and 2021, the age-standardized incidence rate declined marginally (EAPC −0.04; 95% CI −0.04 to −0.03), whereas corresponding rates of prevalence and DALYs increased (EAPC 0.03; 95% CI 0.02–0.04 and 0.04; 95% CI 0.03–0.05, respectively), denoting an expanding disease burden that aligns with Join-point analyses and previous estimates^2^. Decomposition analysis attributed the majority of the absolute increase to population growth, whereas population ageing exerted a marked attenuating effect on incident cases. These patterns underscore the interplay of demographic change, rising psychosocial stressors and environmental risk factors (9, 31, 32). Public-health strategies should therefore priorities mitigation of schizophrenia incidence linked to continued population growth and simultaneously capitalize on the protective effect of ageing, with particular emphasis on middle-, low-middle- and low-SDI settings.

Although age-standardized DALY rates remained stable between 1990 and 2021, absolute DALYs rose by 40.9%, with divergent trends across SDI quintiles. Globally, age-standardized DALY rates were static over 1990–2021, whereas crude DALYs increased by 40.9%, driven primarily by population growth. DALY rates rose in high, high-middle, middle and low-middle SDI quintiles, while the low-SDI quintile showed the smallest absolute increase but continued an upward gradient. In 2021, this gap persisted, plausibly reflecting greater case detection and prolonged survival in high-SDI settings, the cumulative disability of chronic schizophrenia and possible sex-specific risk transitions (9, 16). Concurrently, under-detection and under-reporting attributable to scarce mental-health services and weak surveillance systems in low-SDI settings probably lead to substantial underestimation of true burden. These findings underscore the urgent need to strengthen global schizophrenia care, especially by improving diagnostic capacity, treatment access and community-based support in resource-constrained settings (32, 33).

Marked regional heterogeneity in schizophrenia burden is driven primarily by differential data quality, healthcare capacity and sociopolitical context, rather than by true variation in epidemiological risk. In 2021, Eastern Europe recorded the lowest age-standardized rates among 21 GBD regions: incidence 11.7 (95% UI 9.7–14.2) per 100,000, prevalence 205.8 (169.7–245.8) per 100,000 and DALYs 130.6 (96.5–169.3) per 100,000. This paradox contrasts with the region’s high-middle SDI position and substantial alcohol-related mortality burden. Local evidence suggests that under-reporting, fragmented mental-health information systems and competing mortality risks explain this underestimation (34–36). Conversely, Australasia showed the highest rates, plausibly reflecting persistent colonial-era inequities that increase psychosocial stress (37, 38) and high-performance health systems that prolong survival and improve case detection (39). At the national level, Suriname’s low incidence (10.8 per 100,000; 95% UI 9.4–12.5) and Somalia’s implausibly low prevalence (196.1 per 100,000; 152.1–248.7) exemplify surveillance collapse in fragile states (13, 40, 41). These outliers underscore the inherent limitations of GBD estimates when national health information systems are dysfunctional. Thus, the “low burden” observed in Eastern Europe reflects under-detection, under-diagnosis and competing mortality rather than low true risk. In Australasia, the “high burden” is primarily an artefact of high case ascertainment and longer survival afforded by advanced healthcare. Similarly, the extremely low estimates for fragile countries such as Suriname and Somalia highlight the complete breakdown of routine surveillance.

The SDI demonstrates a U-shaped relationship with schizophrenia burden globally and across all 21 GBD regions. Below SDI ≈ 0.45 and above SDI ≈ 0.65, each unit increase in SDI was positively associated with higher age-standardized DALY rates; this association is plausibly mediated by urbanization, rising psychosocial stressors and differential case-detection capacity (42–45). At the national level, the slope steepened markedly once SDI exceeded 0.76, indicating an accelerated rise in schizophrenia DALYs. This inflection likely reflects the dual influence of advanced health-care systems that enhance case detection and of urbanization-related stressors that increase population vulnerability to psychosis (46–49). Collectively, these data imply that urbanization, higher economic development and associated psychosocial stressors elevate the incidence of psychotic disorders. Consequently, mental-health services must be scaled up in tandem with urban and economic growth to mitigate the emerging schizophrenia burden.

Marked sex differences characterize the epidemiology of schizophrenia. However, our forecast models suggested a disproportionate future increase in schizophrenia-related DALYs among women, driven by structural inequalities, economic precarity and inadequate access to mental-health care, necessitating multilevel interventions that enhance social position, quality of life and service provision. We corroborate that males experience 1.4-fold higher age-standardized incidence rates, with peak burden in young and middle-aged adults, in line with previous estimates (2, 9, 15, 50). This excess is plausibly mediated by heightened early-male neurodevelopmental vulnerability, lower oestrogen-mediated neuroprotection, and greater exposure to cannabis and psychosocial stressors (50–53). However, this male-predominant pattern is poised to reverse. Our ARIMA projections indicate that female incidence, prevalence, and DALYs will rise significantly over the next 15 years, while male burden stabilizes or declines. This epidemiological shift marks a critical inflection point: the current schizophrenia landscape remains male-dominated, but the future burden will increasingly be borne by women. Such a gender crossover challenges the prevailing male-centric paradigm in schizophrenia research and policy, and underscores the urgent need for female-sensitive diagnostics, sex-stratified prevention, and gender-responsive service delivery. Conversely, ARIMA projections indicate a steeper rise in future DALY rates among females, reflecting interactive biological, psychosocial and health-system determinants (53, 54). Consequently, policies must adopt a multisectoral approach that simultaneously tackles social inequities, economic barriers and health-service gaps to improve women’s social position, quality of life and access to psychological and medical care.

Comorbid drug-use disorders (DUD), particularly those involving cannabis, amphetamines, and opioids, represent a major modifiable driver of the global schizophrenia burden (55, 56). According to GBD 2021 estimates, these conditions accounted for 16.0% (2.38 million DALYs) of the total schizophrenia burden worldwide (57). The attributable fraction exhibited a steep socio-demographic gradient, rising from 9.4% in low-SDI countries to 19.5% in high-SDI countries—a pattern consistent with the epidemiological trends described in Section 3.2. Notably, regions with the highest absolute schizophrenia burden, such as Australasia and high-income North America, also recorded the highest cannabis-attributable DALY rates. These findings underscore that substance-use comorbidity is not merely a clinical concern but quantifiably accounts for one in every six schizophrenia-related DALYs globally. Integrating evidence-based DUD interventions—including early detection and combined pharmacological and psychosocial treatments—into schizophrenia care programs is therefore critical for reducing the population-level disease burden, particularly among high-risk groups such as young males in high-SDI settings.

Although we present a comprehensive analysis of global schizophrenia burden, several methodological limitations should be acknowledged. First, it is important to acknowledge that the accuracy of GBD estimates is contingent upon the quality and coverage of the underlying source data, which may lead to underestimation in data-sparse regions. Second, although the GBD analytical platform continues to evolve, model choice and underlying data quality inevitably introduce uncertainty into the estimates. Notwithstanding these limitations, our findings offer empirical evidence to inform policy and prevention strategies aimed at reducing the global schizophrenia burden.

## 5 Conclusions

Schizophrenia continues to impose a substantial and steadily increasing global disease burden, characterized by marked geographic heterogeneity and pronounced sex disparities. Importantly, age-standardized DALY rates exhibit a positive association with SDI once the index exceeds approximately 0.65, indicating elevated risk in upper-middle and high-SDI settings. These findings underscore the need for evidence-based, SDI-stratified prevention and intervention strategies that adapt to the U-shaped relationship between SDI and schizophrenia burden, particularly addressing the increasing burden in low and high SDI regions.

## Data Availability

The original contributions presented in the study are included in the article/**Supplementary Material**. Further inquiries can be directed to the corresponding author.
