# Supplementary material for: Global, regional, and national burden of schizophrenia: epidemiological trends, decomposition, joinpoint analysis, and projections to 2036 based on GBD 2021
Source: Front Psychiatry. 2026 Feb 12;17:1702808. doi: 10.3389/fpsyt.2026.1702808 (PMC12938685; doi:10.3389/fpsyt.2026.1702808)
Supplement: Supplementary file 1 [file DataSheet1.docx]

**Supplemental Tables**

**Supplemental Tables S1** The numbers and ASRs of incidence of schizophrenia burden for the global, 5 SDI regions and 21 GBD regions.

| **Location**  **name** | **1990** | | **2021** | | **1990-2021** | **RC of**  **number**  **s (%)** | **RC of**  **ASR**  **(%)** |
| --- | --- | --- | --- | --- | --- | --- | --- |
|  | **Case number**  **(95% UI)** | **ASR**  **(95% UI)** | **Case number**  **(95% UI)** | **ASR**  **(95% UI)** | **EAPC**  **(95% CI)** |  |  |
| Global | 883493.49(721489.12-1065739.75) | 15.64(13.04-18.62) | 1223221.03(1008219.03-1473083.27) | 15.43(12.74-18.62) | -0.04(-0.04, -0.03) | 38.45 | -1.31 |
| **SDI regions** |  |  |  |  |  |  |  |
| High SDI | 143838.13(118990.99-171970.25) | 15.70(13.01-18.80) | 152489.89(126713.76-183179.81) | 15.69(12.95-18.76) | 0.04(0.00,0.08) | 6.01 | -0.05 |
| High-middle SDI | 184070.11(156454.58-215712.93) | 15.78(13.53-18.40) | 201510.84(172551.09-236757.03) | 16.53(14.15-19.33) | 0.14(0.12,0.15) | 9.48 | 4.77 |
| Middle SDI | 311192.25(253781.37-375053.17) | 16.26(13.65-19.43) | 391070.76(321493.87-471393.43) | 15.74(13.01-18.96) | -0.11(-0.12, -0.10) | 25.67 | -3.22 |
| Low-middle SDI | 175840.37(140125.78-217436.73) | 15.08(12.27-18.34) | 313593.33(251893.83-386296.28) | 15.04(12.21-18.29) | -0.01(-0.02,0.01) | 78.34 | -0.28 |
| Low SDI | 67855.76(52972.93-84442.09) | 14.63(11.83-17.88) | 163692.03(127545.18-203659.37) | 14.54(11.76-17.87) | -0.02(-0.03, -0.01) | 141.24 | -0.59 |
| **Regions** |  |  |  |  |  |  |  |
| Andean Latin America | 5248.90(3963.81-6801.88) | 13.35(10.25-17.01) | 9504.78(7301.51-12140.14) | 13.36(10.31-16.96) | 0.01(-0.00,0.02) | 81.08 | 0.13 |
| Australasia | 4253.69(3755.74-4797.19) | 20.09(17.77-22.63) | 5551.30(4848.82-6304.66) | 20.19(17.87-22.74) | 0.02(0.01,0.02) | 30.51 | 0.53 |
| Caribbean | 4719.77(3596.90-6069.46) | 12.41(9.67-15.64) | 5992.33(4650.50-7578.13) | 12.36(9.55-15.61) | -0.02(-0.04, -0.01) | 26.96 | -0.41 |
| Central Asia | 8960.05(6752.34-11589.46) | 12.44(9.61-15.88) | 12222.24(9355.68-15539.79) | 12.43(9.55-15.74) | -0.00(-0.01,0.00) | 36.41 | -0.07 |
| Central Europe | 15423.44(12258.63-19259.59) | 12.48(9.82-15.62) | 12540.44(10077.54-15505.33) | 12.60(9.97-15.82) | 0.03(0.02,0.03) | -18.69 | 0.95 |
| Central Latin America | 23645.32(18376.15-29734.57) | 13.66(10.92-16.91) | 36521.22(29189.50-45125.04) | 13.61(10.88-16.81) | -0.02(-0.03, -0.01) | 54.45 | -0.31 |
| Central Sub-Saharan Africa | 7089.51(5341.90-9106.03) | 13.88(10.77-17.56) | 18469.35(14119.93-23687.66) | 13.79(10.85-17.57) | -0.02(-0.03, -0.01) | 160.52 | -0.68 |
| East Asia | 259598.60(221425.35-300729.98) | 18.16(15.66-20.98) | 244885.36(209048.18-286291.68) | 18.32(15.77-21.17) | -0.04(-0.07, -0.01) | -5.67 | 0.89 |
| Eastern Europe | 25246.62(20671.80-30695.69) | 11.18(9.18-13.55) | 21245.22(17519.41-25696.32) | 11.73(9.65-14.21) | 0.23(0.18,0.28) | -15.85 | 4.95 |
| Eastern Sub-Saharan Africa | 24723.70(19099.27-30991.46) | 14.29(11.56-17.68) | 60847.20(47224.88-76142.64) | 14.16(11.46-17.53) | -0.02(-0.03, -0.02) | 146.11 | -0.91 |
| High-income Asia Pacific | 27504.91(22411.46-33376.27) | 15.04(12.24-18.31) | 22803.86(18712.43-27722.61) | 15.14(12.25-18.55) | 0.14(0.06,0.22) | -17.09 | 0.66 |
| High-income North America | 50619.73(41922.84-60930.94) | 17.41(14.42-20.77) | 54816.57(45915.08-65191.14) | 16.68(13.81-19.82) | -0.09(-0.15, -0.04) | 8.29 | -4.16 |
| North Africa and Middle East | 48836.09(37285.68-61571.75) | 14.21(11.23-17.66) | 93042.34(73553.26-116456.47) | 14.07(11.05-17.66) | -0.04(-0.04, -0.03) | 90.52 | -0.96 |
| Oceania | 1142.60(863.06-1462.92) | 17.04(13.34-21.36) | 2473.21(1911.94-3183.78) | 17.05(13.41-21.67) | -0.00(-0.02,0.01) | 116.45 | 0.1 |
| South Asia | 170021.36(136997.38-208810.08) | 15.20(12.42-18.42) | 314672.71(253849.44-382451.66) | 15.26(12.45-18.51) | 0.02(-0.01,0.04) | 85.08 | 0.41 |
| Southeast Asia | 86321.16(68048.37-106067.30) | 17.23(14.03-21.06) | 127997.46(104673.60-155998.79) | 17.34(14.18-21.06) | 0.07(0.05,0.09) | 48.28 | 0.6 |
| Southern Latin America | 7608.64(5709.97-9693.48) | 15.23(11.56-19.33) | 10585.24(8109.86-13526.02) | 15.25(11.64-19.37) | -0.01(-0.02, -0.00) | 39.12 | 0.13 |
| Southern Sub-Saharan Africa | 7493.90(5979.39-9179.64) | 13.94(11.41-16.96) | 12040.08(9761.64-14601.56) | 13.83(11.29-16.73) | -0.01(-0.02, -0.01) | 60.67 | -0.75 |
| Tropical Latin America | 22629.93(18290.29-27506.72) | 13.75(11.31-16.61) | 32547.27(26913.62-39418.73) | 13.75(11.35-16.60) | -0.00(-0.01,0.01) | 43.82 | 0.01 |
| Western Europe | 55778.71(46652.05-66798.31) | 14.21(11.84-16.95) | 53702.01(45054.83-64511.45) | 14.08(11.57-16.97) | -0.02(-0.04, -0.01) | -3.72 | -0.89 |
| Western Sub-Saharan Africa | 26626.87(20950.99-33116.54) | 14.97(12.19-18.43) | 70760.83(55512.52-87820.46) | 14.95(12.13-18.41) | -0.01(-0.02, -0.01) | 165.75 | -0.1 |

**Supplemental Tables S2** The numbers and ASRs of prevalence of schizophrenia burden for the global, 5 SDI regions and 21 GBD regions.

| **Location**  **name** | **1990** | | **2021** | | **1990-2021** | **RC of**  **number**  **s (%)** | **RC of**  **ASR**  **(%)** |
| --- | --- | --- | --- | --- | --- | --- | --- |
|  | **Case number**  **(95% UI)** | **ASR**  **(95% UI)** | **Case number**  **(95% UI)** | **ASR**  **(95% UI)** | **EAPC**  **(95% CI)** |  |  |
| Global | 13621402.27(11333556.70-16166722.18) | 275.78(229.94-324.02) | 23182108.90(19203758.75-27423876.46) | 277.71(229.77-329.06) | 0.03(0.02,0.04) | 70.19 | 0.7 |
| **SDI regions** |  |  |  |  |  |  |  |
| High SDI | 2952553.70(2460547.92-3473575.55) | 299.93(249.94-353.06) | 3837953.50(3233342.78-4488357.02) | 296.45(247.61-349.30) | -0.00(-0.04,0.03) | 29.99 | -1.16 |
| High-middle SDI | 2939653.14(2511212.34-3401078.04) | 265.69(227.19-306.34) | 4484411.56(3871242.16-5119819.65) | 286.65(246.57-329.25) | 0.22(0.20,0.24) | 52.55 | 7.89 |
| Middle SDI | 4318248.49(3584548.58-5127622.65) | 276.81(231.87-325.85) | 7591052.75(6294522.08-8991069.29) | 278.26(230.98-330.63) | 0.02(0.00,0.03) | 75.79 | 0.52 |
| Low-middle SDI | 2518085.20(2034913.17-3059205.88) | 270.34(220.90-325.04) | 5127385.41(4146444.23-6190285.48) | 273.43(224.02-328.22) | 0.05(0.03,0.07) | 103.62 | 1.14 |
| Low SDI | 881534.55(706902.12-1088125.71) | 243.17(197.43-294.98) | 2124992.02(1699837.56-2616104.31) | 243.92(197.83-297.13) | 0.03(0.01,0.04) | 141.06 | 0.31 |
| **Regions** |  |  |  |  |  |  |  |
| Andean Latin America | 72584.54(55904.64-93817.78) | 236.55(185.40-299.32) | 162743.38(127620.42-205736.74) | 239.92(188.89-300.78) | 0.05(0.04,0.06) | 124.21 | 1.42 |
| Australasia | 85195.33(77871.14-92735.52) | 386.61(353.38-420.60) | 134947.53(123734.31-146497.57) | 387.08(355.39-420.53) | 0.01(0.01,0.01) | 58.4 | 0.12 |
| Caribbean | 71392.71(55514.30-90536.44) | 222.99(175.25-279.25) | 112862.12(89833.92-140947.28) | 222.19(176.38-278.19) | -0.01(-0.02,0.00) | 58.09 | -0.36 |
| Central Asia | 129136.73(99439.53-165054.18) | 213.71(166.39-269.11) | 216293.79(167406.48-273702.53) | 215.61(167.53-271.96) | 0.05(0.03,0.07) | 67.49 | 0.89 |
| Central Europe | 299470.35(238748.18-367673.23) | 219.54(173.79-270.73) | 312508.10(255244.22-382643.08) | 224.46(179.00-276.40) | 0.08(0.07,0.08) | 4.35 | 2.24 |
| Central Latin America | 321169.83(256344.32-397632.80) | 242.99(197.60-295.28) | 653292.10(528794.48-797930.02) | 243.71(197.48-297.44) | 0.01(0.00,0.02) | 103.41 | 0.29 |
| Central Sub-Saharan Africa | 84684.74(64946.74-109579.59) | 215.78(168.71-273.87) | 223795.78(169956.84-287507.04) | 212.73(165.35-266.97) | -0.03(-0.06, -0.00) | 164.27 | -1.42 |
| East Asia | 3677016.25(3168631.92-4222686.70) | 300.20(259.78-343.13) | 5498241.03(4783147.46-6253121.90) | 311.41(270.11-356.30) | 0.06(0.04,0.09) | 49.53 | 3.74 |
| Eastern Europe | 493061.08(409730.08-588697.07) | 195.34(160.70-234.34) | 513418.58(429002.54-604368.48) | 205.80(169.71-245.81) | 0.26(0.20,0.32) | 4.13 | 5.35 |
| Eastern Sub-Saharan Africa | 281115.46(221152.40-353361.44) | 214.52(173.50-263.96) | 710915.02(560238.36-886943.61) | 216.90(175.26-266.07) | 0.05(0.05,0.06) | 152.89 | 1.11 |
| High-income Asia Pacific | 544321.64(450438.56-650697.60) | 276.12(227.68-330.81) | 603908.80(500971.63-713804.71) | 271.65(221.53-328.49) | 0.05(-0.02,0.12) | 10.95 | -1.62 |
| High-income North America | 1131991.55(957789.17-1321674.96) | 362.14(306.42-422.69) | 1458582.41(1252650.09-1685610.58) | 347.65(296.76-402.86) | -0.09(-0.14, -0.04) | 28.85 | -4 |
| North Africa and Middle East | 650417.56(511037.46-811854.40) | 243.50(193.62-299.35) | 1574967.69(1251996.52-1957684.33) | 244.94(195.26-301.81) | 0.02(0.02,0.03) | 142.15 | 0.59 |
| Oceania | 14477.58(10957.07-18633.22) | 274.85(213.20-345.65) | 34436.01(26449.55-44207.32) | 274.77(213.32-347.47) | -0.01(-0.03,0.00) | 137.86 | -0.03 |
| South Asia | 2595651.25(2130584.06-3094140.36) | 287.60(238.39-340.76) | 5536958.29(4558070.35-6588274.83) | 294.36(243.49-348.60) | 0.09(0.06,0.12) | 113.32 | 2.35 |
| Southeast Asia | 1142606.71(914321.49-1407312.48) | 280.98(227.47-339.99) | 2209594.56(1791364.44-2672002.18) | 290.41(235.84-351.11) | 0.16(0.13,0.19) | 93.38 | 3.35 |
| Southern Latin America | 132890.02(103984.70-167191.66) | 277.60(217.06-348.42) | 209688.39(163480.27-263330.96) | 278.76(217.86-351.60) | 0.01(-0.00,0.02) | 57.79 | 0.42 |
| Southern Sub-Saharan Africa | 94100.01(75882.76-115041.09) | 223.91(183.31-269.51) | 181544.11(147510.08-220321.60) | 224.70(184.62-271.20) | 0.03(0.03,0.04) | 92.93 | 0.35 |
| Tropical Latin America | 325817.64(265949.77-392477.27) | 240.98(199.53-286.79) | 624741.24(517173.00-743686.45) | 243.34(201.46-290.42) | 0.04(0.03,0.05) | 91.75 | 0.98 |
| Western Europe | 1149136.98(969245.48-1358309.02) | 260.06(217.15-308.91) | 1346135.25(1137842.90-1579942.47) | 256.54(213.26-306.09) | -0.03(-0.06, -0.01) | 17.14 | -1.35 |
| Western Sub-Saharan Africa | 325164.31(259749.38-404778.68) | 233.05(189.54-283.76) | 862534.73(686972.85-1070550.61) | 237.21(192.63-289.33) | 0.08(0.07,0.09) | 165.26 | 1.78 |

**Supplemental Tables S3** The numbers and ASRs of DALYs of schizophrenia burden for the global, 5 SDI regions and 21 GBD regions.

| **Location**  **name** | **1990** | | **2021** | | **1990-2021** | **RC of**  **number**  **s (%)** | **RC of**  **ASR**  **(%)** |
| --- | --- | --- | --- | --- | --- | --- | --- |
|  | **Case number**  **(95% UI)** | **ASR**  **(95% UI)** | **Case number**  **(95% UI)** | **ASR**  **(95% UI)** | **EAPC**  **(95% CI)** |  |  |
| Global | 8762311.80(6477261.17-11263749.70) | 176.61(130.85-226.02) | 14816611.18(10926460.05-19095362.42) | 177.75(131.51-228.80) | 0.04(0.03,0.05) | 69.09 | 0.64 |
| **SDI regions** | | | | | | | |
| High SDI | 1884298.84(1399687.31-2411617.86) | 191.78(142.73-245.66) | 2418425.13(1798078.75-3069349.31) | 188.61(140.84-241.79) | -0.01(-0.05,0.03) | 28.35 | -1.65 |
| High-middle SDI | 1899957.68(1421043.18-2407953.21) | 171.35(127.92-216.46) | 2879223.22(2139705.43-3609613.96) | 185.37(138.35-233.14) | 0.24(0.22,0.26) | 51.54 | 8.18 |
| Middle SDI | 2801552.42(2077439.18-3596383.47) | 178.26(132.35-227.98) | 4875339.59(3592613.46-6250839.31) | 178.99(132.63-229.93) | 0.02(0.01,0.04) | 74.02 | 0.41 |
| Low-middle SDI | 1609237.27(1180195.06-2103025.30) | 171.40(125.35-223.05) | 3273931.87(2394047.46-4314384.51) | 173.74(127.97-227.77) | 0.07(0.05,0.09) | 103.45 | 1.36 |
| Low SDI | 560005.13(400560.25-743413.45) | 153.19(110.14-201.11) | 1359273.61(980588.32-1803202.73) | 154.51(112.40-202.64) | 0.06(0.04,0.08) | 142.73 | 0.86 |
| **Region**s | | | | | | | |
| Andean Latin America | 47049.49(33025.25-65165.16) | 152.22(106.12-206.80) | 104569.76(73439.86-143714.87) | 153.79(107.21-210.87) | 0.06(0.05,0.07) | 122.25 | 1.03 |
| Australasia | 54185.09(40430.80-66507.07) | 246.10(183.50-302.09) | 85419.40(64460.39-103992.02) | 246.81(186.31-300.92) | 0.01(0.01,0.02) | 57.64 | 0.29 |
| Caribbean | 45978.50(32483.56-63107.60) | 142.96(99.86-195.01) | 71833.40(51417.06-97073.96) | 141.64(101.17-191.39) | -0.01(-0.02,-0.00) | 56.23 | -0.92 |
| Central Asia | 83394.33(58566.93-114805.60) | 137.24(96.80-186.75) | 139157.77(98002.52-188924.68) | 138.46(98.16-187.81) | 0.06(0.04,0.09) | 66.87 | 0.88 |
| Central Europe | 190571.02(136791.25-254061.31) | 140.16(99.74-187.76) | 197736.70(140249.59-261296.20) | 143.83(102.58-192.20) | 0.10(0.09,0.11) | 3.76 | 2.62 |
| Central Latin America | 206522.38(150230.74-274689.11) | 154.82(111.50-204.80) | 416368.33(300517.23-546016.77) | 155.23(112.09-203.52) | 0.01(0.00,0.02) | 101.61 | 0.26 |
| Central Sub-Saharan Africa | 53272.75(36839.11-72947.05) | 134.54(93.39-182.49) | 142342.83(101460.81-194608.70) | 134.01(94.98-180.41) | 0.02(-0.01,0.05) | 167.2 | -0.39 |
| East Asia | 2406751.21(1820500.07-3020636.24) | 195.28(147.49-244.06) | 3559796.60(2653372.10-4460256.68) | 203.25(151.83-255.72) | 0.08(0.05,0.11) | 47.91 | 4.08 |
| Eastern Europe | 311677.19(228697.86-402512.78) | 123.94(91.10-160.79) | 322426.56(234984.60-411202.55) | 130.60(96.54-169.32) | 0.28(0.22,0.35) | 3.45 | 5.38 |
| Eastern Sub-Saharan Africa | 179220.92(128318.13-238903.57) | 135.56(96.62-180.07) | 455959.59(328390.07-611300.59) | 137.72(99.56-182.66) | 0.09(0.08,0.11) | 154.41 | 1.59 |
| High-income Asia Pacific | 351503.77(257850.59-455383.34) | 178.60(130.94-232.88) | 386428.90(283590.62-497873.22) | 176.16(129.13-228.66) | 0.06(-0.01,0.13) | 9.94 | -1.36 |
| High-income North America | 718244.95(533903.96-907333.04) | 230.05(170.47-290.87) | 904417.32(677980.38-1130831.10) | 217.61(163.91-273.31) | -0.12(-0.18,-0.07) | 25.92 | -5.41 |
| North Africa and Middle East | 418311.12(300207.10-560220.33) | 155.47(111.33-207.70) | 1005508.08(723247.30-1352121.04) | 155.75(113.05-208.94) | 0.02(0.01,0.02) | 140.37 | 0.18 |
| Oceania | 9356.72(6534.73-12927.59) | 176.01(123.12-239.72) | 22230.12(15860.36-30696.18) | 176.00(124.92-242.09) | -0.01(-0.02,0.01) | 137.58 | -0.01 |
| South Asia | 1654224.20(1222741.94-2138134.12) | 181.72(134.38-234.54) | 3525714.89(2621573.86-4589423.23) | 186.57(138.37-241.76) | 0.11(0.08,0.14) | 113.13 | 2.67 |
| Southeast Asia | 743647.53(543820.85-992006.26) | 181.46(132.16-237.19) | 1434796.24(1038572.29-1879530.36) | 188.39(136.54-246.70) | 0.18(0.15,0.21) | 92.94 | 3.82 |
| Southern Latin America | 84968.57(59895.24-114349.51) | 177.36(124.81-238.44) | 133110.34(93082.66-180218.95) | 177.40(123.82-240.29) | 0.01(-0.00,0.02) | 56.66 | 0.02 |
| Southern Sub-Saharan Africa | 60025.25(43878.51-78352.47) | 141.66(103.60-184.10) | 113604.63(84192.94-148079.38) | 139.89(103.86-182.73) | -0.02(-0.03,-0.00) | 89.26 | -1.25 |
| Tropical Latin America | 207296.86(153327.29-266944.78) | 152.15(112.31-195.75) | 393257.59(286664.07-504323.12) | 153.38(112.43-197.40) | 0.05(0.04,0.07) | 89.71 | 0.81 |
| Western Europe | 728885.29(532945.74-936539.15) | 165.76(122.24-213.89) | 847986.83(619730.25-1085059.65) | 163.48(121.07-212.63) | -0.02(-0.05,0.00) | 16.34 | -1.37 |
| Western Sub-Saharan Africa | 207224.65(148970.40-275372.67) | 147.38(105.48-194.73) | 553945.30(396420.03-734734.69) | 150.91(108.56-198.24) | 0.11(0.10,0.12) | 167.32 | 2.4 |

**Supplemental Tables S4** The numbers and ASR of incidences of schizophrenia burden for the 204 countries/regions.

| Location | 1990 Case number  (95% UI) | 1990 ASR  (95% UI) | 2021 Case number  (95% UI) | 2021 ASR  (95% UI) | EAPCs  (95% CI) | RC of numbers (%) | RC of ASR (%) |
| --- | --- | --- | --- | --- | --- | --- | --- |
| Afghanistan | 1139.14(855.27-1477.75) | 13.59(10.54-17.07) | 4251.06(3136.45-5559.05) | 13.48(10.46-17.09) | -0.04(-0.06, -0.03) | 273.18 | -0.83 |
| Albania | 448.17(338.89-576.72) | 12.40(9.67-15.79) | 326.10(250.06-417.10) | 12.49(9.49-15.98) | 0.01(0.00,0.01) | -27.24 | 0.72 |
| Algeria | 3723.52(2751.39-4800.96) | 14.33(11.12-18.19) | 6298.88(4841.12-8089.45) | 14.18(10.82-18.18) | -0.05(-0.05, -0.04) | 69.16 | -1.07 |
| American Samoa | 9.13(6.85-11.67) | 17.77(13.72-22.28) | 8.40(6.53-10.45) | 17.53(13.72-22.16) | -0.03(-0.04,-0.02) | -7.97 | -1.33 |
| Andorra | 9.58(7.39-12.40) | 14.75(11.46-18.76) | 11.72(9.25-14.68) | 14.66(11.32-18.62) | -0.02(-0.02, -0.01) | 22.44 | -0.58 |
| Angola | 1344.56(1020.06-1713.06) | 13.91(10.76-17.69) | 4222.28(3194.83-5453.13) | 13.91(10.91-17.67) | 0.01(-0.00,0.01) | 214.03 | 0.02 |
| Antigua and Barbuda | 8.35(6.30-10.84) | 12.77(9.76-16.16) | 11.97(9.33-15.09) | 12.73(9.74-16.22) | 0.00(-0.01,0.02) | 43.22 | -0.29 |
| Argentina | 4901.73(3698.82-6192.17) | 15.21(11.55-19.25) | 7153.94(5444.31-9081.18) | 15.23(11.51-19.33) | -0.02(-0.02, -0.01) | 45.95 | 0.1 |
| Armenia | 461.73(354.72-599.20) | 12.59(9.74-16.13) | 359.06(278.39-454.78) | 12.59(9.72-16.07) | -0.01(-0.02,0.00) | -22.24 | 0.06 |
| Australia | 3518.20(3134.91-3977.42) | 20.03(17.95-22.50) | 4560.50(4021.28-5203.38) | 20.10(17.78-22.49) | 0.02(0.01,0.02) | 29.63 | 0.33 |
| Austria | 1182.19(911.19-1501.18) | 14.45(11.13-18.24) | 1184.60(939.79-1496.12) | 14.46(11.24-18.31) | 0.00(-0.00,0.01) | 0.2 | 0.05 |
| Azerbaijan | 1014.58(765.98-1327.67) | 12.54(9.67-16.13) | 1411.10(1089.69-1807.64) | 12.60(9.64-15.97) | 0.02(0.01,0.04) | 39.08 | 0.47 |
| Bahamas | 37.86(28.25-49.44) | 12.73(9.91-16.26) | 52.68(40.92-66.99) | 12.59(9.70-16.07) | -0.03(-0.03, -0.03) | 39.14 | -1.14 |
| Bahrain | 93.81(70.99-122.36) | 14.80(11.37-18.81) | 274.03(208.19-348.50) | 14.79(11.15-18.70) | -0.00(-0.01,0.00) | 192.1 | -0.07 |
| Bangladesh | 16247.04(12152.73-20896.78) | 14.97(11.36-18.90) | 26139.95(19829.05-33046.54) | 14.52(11.10-18.35) | -0.11(-0.12, -0.11) | 60.89 | -2.97 |
| Barbados | 40.95(33.35-49.27) | 14.93(12.27-17.90) | 39.51(31.16-49.17) | 14.04(10.85-17.84) | -0.06(-0.10, -0.03) | -3.5 | -5.97 |
| Belarus | 1213.35(938.34-1561.37) | 11.64(8.99-14.93) | 950.82(739.35-1193.09) | 11.70(8.78-14.84) | 0.01(0.00,0.02) | -21.64 | 0.43 |
| Belgium | 1458.73(1144.30-1850.06) | 14.44(11.28-18.38) | 1454.70(1143.13-1820.55) | 14.37(11.01-18.15) | -0.01(-0.01,0.00) | -0.28 | -0.53 |
| Belize | 22.83(17.14-29.77) | 12.47(9.65-15.82) | 59.98(45.97-75.94) | 12.37(9.62-15.59) | -0.04(-0.05, -0.04) | 162.69 | -0.83 |
| Benin | 616.03(464.95-790.15) | 14.69(11.36-18.51) | 1889.29(1431.43-2420.43) | 14.59(11.39-18.54) | -0.04(-0.05, -0.03) | 206.69 | -0.63 |
| Bermuda | 8.66(6.59-11.08) | 13.04(9.91-16.59) | 6.75(5.38-8.44) | 13.04(10.04-16.67) | 0.01(-0.00,0.02) | -22.11 | 0.06 |
| Bhutan | 104.66(78.34-135.45) | 15.11(11.49-19.24) | 132.57(100.14-170.91) | 14.85(11.29-18.89) | -0.06(-0.07, -0.06) | 26.66 | -1.71 |
| Bolivia (Plurinational State of) | 827.44(624.41-1055.87) | 13.21(10.21-16.87) | 1685.44(1293.38-2145.77) | 13.19(10.22-16.67) | -0.00(-0.01,0.00) | 103.69 | -0.13 |
| Bosnia and Herzegovina | 610.20(466.29-778.75) | 12.31(9.46-15.59) | 360.75(280.51-452.64) | 12.49(9.39-15.98) | 0.04(0.03,0.05) | -40.88 | 1.46 |
| Botswana | 174.22(131.58-221.67) | 13.78(10.73-17.41) | 375.35(290.08-480.95) | 13.88(10.82-17.68) | 0.03(0.02,0.04) | 115.44 | 0.74 |
| Brazil | 22093.63(17891.38-26817.18) | 13.76(11.33-16.60) | 31483.75(26104.61-38044.31) | 13.76(11.38-16.59) | 0.00(-0.01,0.01) | 42.5 | 0.02 |
| Brunei Darussalam | 44.47(33.61-58.11) | 14.56(11.24-18.67) | 77.46(59.84-98.81) | 14.42(11.17-18.25) | -0.02(-0.03, -0.02) | 74.17 | -0.91 |
| Bulgaria | 986.61(769.65-1247.27) | 12.04(9.29-15.36) | 663.69(524.14-839.86) | 12.07(9.21-15.43) | 0.01(0.00,0.01) | -32.73 | 0.28 |
| Burkina Faso | 1158.87(882.66-1478.18) | 14.61(11.48-18.25) | 3063.08(2317.41-3950.82) | 14.49(11.24-18.18) | -0.04(-0.05, -0.03) | 164.32 | -0.8 |
| Burundi | 711.99(541.91-923.92) | 14.07(10.90-17.97) | 1781.36(1366.13-2281.06) | 13.98(10.97-17.77) | -0.01(-0.02, -0.01) | 150.19 | -0.64 |
| Cabo Verde | 47.82(35.16-62.20) | 14.83(11.39-18.74) | 94.23(71.55-119.81) | 14.75(11.39-18.59) | -0.03(-0.04, -0.02) | 97.06 | -0.53 |
| Cambodia | 1606.78(1211.96-2057.46) | 16.43(12.88-20.74) | 3075.96(2373.38-3874.52) | 16.68(12.99-20.93) | 0.09(0.06,0.12) | 91.44 | 1.56 |
| Cameroon | 1380.67(1046.40-1788.61) | 14.61(11.33-18.60) | 4583.50(3480.01-5892.88) | 14.47(11.34-18.23) | -0.04(-0.05, -0.03) | 231.98 | -1 |
| Canada | 3541.08(3302.79-3868.95) | 12.57(11.80-13.61) | 4185.39(3890.38-4572.31) | 13.22(12.40-14.29) | 0.15(0.11,0.20) | 18.2 | 5.11 |
| Central African Republic | 352.99(268.97-454.57) | 13.72(10.86-17.26) | 735.34(557.15-933.55) | 13.64(10.79-17.05) | -0.01(-0.02, -0.01) | 108.32 | -0.58 |
| Chad | 751.59(568.54-960.84) | 14.59(11.40-18.46) | 2225.86(1671.39-2866.30) | 14.52(11.45-18.27) | -0.02(-0.02, -0.01) | 196.16 | -0.48 |
| Chile | 2245.18(1686.09-2876.49) | 15.25(11.64-19.41) | 2931.84(2255.34-3732.29) | 15.30(11.69-19.49) | 0.00(-0.01,0.01) | 30.58 | 0.29 |
| China | 251819.87(215196.35-291260.66) | 18.19(15.71-20.97) | 236174.66(201683.92-275708.46) | 18.36(15.86-21.18) | -0.04(-0.07, -0.01) | -6.21 | 0.95 |
| Colombia | 4785.23(3602.17-6242.32) | 13.41(10.27-17.19) | 7153.74(5457.50-9255.59) | 13.43(10.23-17.28) | -0.00(-0.01,0.01) | 49.5 | 0.12 |
| Comoros | 61.06(45.74-77.89) | 14.26(11.06-17.97) | 112.93(86.70-145.08) | 14.18(11.04-18.06) | -0.00(-0.01,0.00) | 84.97 | -0.52 |
| Congo | 322.74(241.47-413.30) | 13.89(10.87-17.33) | 781.42(598.53-1001.36) | 13.83(10.75-17.68) | 0.00(-0.01,0.01) | 142.12 | -0.4 |
| Cook Islands | 3.53(2.65-4.51) | 17.78(13.68-22.42) | 2.97(2.32-3.70) | 17.88(13.94-22.60) | 0.02(0.01,0.03) | -15.87 | 0.54 |
| Costa Rica | 442.62(335.54-568.39) | 13.56(10.47-17.22) | 682.31(527.41-870.50) | 13.55(10.46-17.39) | -0.01(-0.01, -0.00) | 54.15 | -0.08 |
| Croatia | 612.19(474.77-776.70) | 12.53(9.64-15.98) | 449.71(351.33-565.42) | 12.61(9.70-16.23) | 0.01(0.01,0.02) | -26.54 | 0.68 |
| Cuba | 1611.29(1210.68-2111.97) | 12.73(9.73-16.27) | 1297.08(1020.39-1613.94) | 12.65(9.74-16.01) | -0.02(-0.03, -0.01) | -19.5 | -0.64 |
| Cyprus | 117.81(91.38-148.53) | 14.47(11.26-18.13) | 200.16(156.16-253.19) | 14.48(11.18-18.24) | -0.00(-0.01,0.00) | 69.9 | 0.13 |
| Czechia | 1274.02(987.24-1601.79) | 12.80(9.69-16.32) | 1109.11(877.73-1390.93) | 12.87(9.98-16.47) | -0.00(-0.01,0.01) | -12.94 | 0.59 |
| C么te d’Ivoire | 1687.55(1272.84-2189.41) | 14.47(11.32-18.46) | 4024.47(3052.61-5146.48) | 14.45(11.15-18.22) | -0.02(-0.02, -0.01) | 138.48 | -0.2 |
| Democratic People’s Republic of Korea | 3754.64(2922.65-4758.32) | 16.96(13.34-21.12) | 4633.13(3692.86-5730.96) | 16.67(13.21-20.83) | -0.08(-0.10, -0.07) | 23.4 | -1.67 |
| Democratic Republic of the Congo | 4882.72(3685.74-6287.53) | 13.88(10.76-17.65) | 12221.72(9256.75-15784.97) | 13.74(10.82-17.53) | -0.03(-0.04, -0.03) | 150.31 | -1 |
| Denmark | 761.42(620.80-918.07) | 14.20(11.62-17.31) | 1117.77(1022.69-1170.61) | 21.28(19.39-22.28) | 1.85(1.49,2.20) | 46.8 | 49.83 |
| Djibouti | 61.18(46.20-78.77) | 14.18(11.12-17.80) | 194.70(150.59-246.58) | 14.10(11.02-17.73) | -0.01(-0.02, -0.00) | 218.22 | -0.58 |
| Dominica | 9.42(7.11-12.32) | 12.62(9.81-15.99) | 8.66(6.73-10.92) | 12.52(9.68-15.88) | -0.04(-0.04, -0.03) | -8.07 | -0.81 |
| Dominican Republic | 957.88(718.32-1250.54) | 12.41(9.58-15.76) | 1490.12(1136.48-1899.49) | 12.56(9.62-15.88) | 0.04(0.03,0.04) | 55.57 | 1.15 |
| Ecuador | 1397.08(1049.76-1830.72) | 13.36(10.25-17.13) | 2557.02(1955.11-3292.22) | 13.30(10.22-16.95) | 0.00(-0.01,0.01) | 83.03 | -0.48 |
| Egypt | 7994.85(5937.67-10208.42) | 14.15(10.88-17.98) | 15384.99(11810.24-19623.40) | 14.07(10.89-17.89) | -0.03(-0.04, -0.02) | 92.44 | -0.53 |
| El Salvador | 695.77(526.07-895.52) | 13.08(10.13-16.43) | 894.23(675.76-1145.24) | 13.04(10.09-16.58) | -0.01(-0.02, -0.00) | 28.52 | -0.3 |
| Equatorial Guinea | 52.02(39.56-66.96) | 13.89(10.88-17.65) | 239.01(179.49-307.98) | 14.03(10.89-17.78) | 0.08(0.06,0.10) | 359.48 | 0.99 |
| Eritrea | 447.36(337.20-570.28) | 14.10(10.99-17.69) | 975.62(742.40-1242.24) | 13.99(10.99-17.62) | -0.01(-0.01,0.00) | 118.08 | -0.78 |
| Estonia | 178.82(139.89-225.51) | 11.72(9.03-14.75) | 130.45(102.93-164.58) | 11.85(9.08-15.09) | 0.04(0.03,0.04) | -27.05 | 1.12 |
| Eswatini | 102.31(76.80-131.94) | 13.79(10.76-17.29) | 173.84(131.33-221.60) | 13.72(10.59-17.25) | -0.00(-0.01,0.01) | 69.91 | -0.49 |
| Ethiopia | 6630.22(5368.27-7968.04) | 14.76(12.30-17.62) | 16487.63(13296.65-19789.72) | 14.55(12.15-17.37) | -0.05(-0.05, -0.04) | 148.67 | -1.43 |
| Fiji | 141.04(106.83-179.22) | 17.25(13.36-21.69) | 163.83(128.59-205.55) | 17.35(13.61-21.84) | 0.02(0.01,0.04) | 16.16 | 0.62 |
| Finland | 735.93(622.25-876.68) | 14.49(12.14-17.19) | 653.64(520.04-812.70) | 13.68(10.69-17.20) | -0.19(-0.24, -0.14) | -11.18 | -5.53 |
| France | 8385.37(6549.73-10482.71) | 14.28(11.06-17.73) | 8142.53(6472.92-10167.10) | 14.23(11.16-18.06) | -0.02(-0.03, -0.01) | -2.9 | -0.36 |
| Gabon | 134.47(100.87-173.53) | 14.06(10.81-17.90) | 269.57(207.86-345.60) | 14.08(10.93-17.77) | 0.00(-0.00,0.01) | 100.47 | 0.17 |
| Gambia | 135.53(101.23-173.50) | 14.61(11.47-18.53) | 353.86(267.42-451.71) | 14.50(11.27-18.24) | -0.02(-0.03, -0.01) | 161.1 | -0.73 |
| Georgia | 706.33(542.28-901.50) | 12.57(9.68-16.08) | 393.01(307.26-493.57) | 12.53(9.50-15.90) | -0.01(-0.02,0.00) | -44.36 | -0.29 |
| Germany | 11426.56(9020.34-14121.50) | 13.80(10.97-17.12) | 10195.69(8079.85-12573.57) | 13.80(10.77-17.16) | -0.01(-0.03,0.00) | -10.77 | 0 |
| Ghana | 2070.20(1549.54-2660.32) | 14.60(11.31-18.45) | 5230.14(3990.10-6681.84) | 14.56(11.30-18.25) | -0.00(-0.01,0.00) | 152.64 | -0.26 |
| Greece | 1487.09(1158.79-1862.60) | 14.43(11.23-18.19) | 1226.39(971.99-1534.32) | 14.35(11.03-18.15) | -0.02(-0.02, -0.01) | -17.53 | -0.5 |
| Greenland | 10.76(8.02-13.86) | 15.30(11.62-19.18) | 8.13(6.30-10.26) | 15.18(11.64-19.01) | 0.05(0.01,0.08) | -24.46 | -0.78 |
| Grenada | 10.48(7.96-13.63) | 12.51(9.83-15.94) | 13.86(10.65-17.67) | 12.59(9.74-15.93) | 0.02(0.01,0.02) | 32.33 | 0.58 |
| Guam | 29.61(22.44-37.86) | 18.37(14.21-23.18) | 27.86(21.75-34.88) | 18.18(14.14-22.80) | -0.02(-0.03, -0.01) | -5.89 | -1.02 |
| Guatemala | 982.29(741.50-1261.27) | 13.06(10.04-16.70) | 2275.70(1737.87-2928.05) | 13.01(10.12-16.57) | -0.02(-0.03, -0.01) | 131.67 | -0.37 |
| Guinea | 759.08(578.73-961.25) | 14.66(11.45-18.39) | 1840.45(1386.68-2316.78) | 14.53(11.38-18.09) | -0.02(-0.03, -0.01) | 142.46 | -0.85 |
| Guinea-Bissau | 131.08(99.08-167.76) | 14.49(11.43-18.28) | 296.19(225.27-381.68) | 14.44(11.36-18.26) | -0.02(-0.03, -0.01) | 125.97 | -0.37 |
| Guyana | 103.62(77.53-133.99) | 12.26(9.60-15.52) | 101.22(77.85-128.51) | 12.25(9.54-15.28) | -0.01(-0.01, -0.00) | -2.32 | -0.05 |
| Haiti | 740.04(568.77-945.71) | 12.07(9.44-15.12) | 1656.39(1285.64-2085.42) | 11.95(9.35-15.03) | -0.04(-0.05, -0.04) | 123.82 | -0.95 |
| Honduras | 572.71(431.56-732.49) | 13.14(10.19-16.57) | 1464.76(1115.18-1883.93) | 13.01(10.06-16.62) | -0.04(-0.05, -0.04) | 155.76 | -0.99 |
| Hungary | 1224.05(942.76-1559.29) | 12.40(9.47-15.90) | 1016.47(793.50-1271.99) | 12.53(9.51-16.04) | 0.02(0.01,0.03) | -16.96 | 1.04 |
| Iceland | 39.08(29.89-49.19) | 14.56(11.22-18.19) | 48.53(38.26-60.74) | 14.56(11.38-18.47) | -0.00(-0.01,0.00) | 24.17 | 0 |
| India | 133991.46(108496.25-164227.50) | 15.16(12.46-18.27) | 243679.25(198452.49-295371.75) | 15.32(12.55-18.60) | 0.05(0.02,0.08) | 81.86 | 1.11 |
| Indonesia | 33902.80(27688.40-40733.25) | 17.03(14.30-20.37) | 51986.31(43248.39-62004.01) | 17.14(14.32-20.43) | 0.07(0.04,0.10) | 53.34 | 0.62 |
| Iran (Islamic Republic of) | 8041.74(6484.53-9687.67) | 14.43(11.93-17.38) | 12729.42(10396.88-15432.30) | 14.35(11.87-17.24) | -0.03(-0.03,-0.02) | 58.29 | -0.53 |
| Iraq | 2608.48(1922.81-3350.94) | 14.20(10.72-17.91) | 6422.19(4898.50-8182.00) | 14.10(10.84-17.76) | -0.03(-0.04,-0.02) | 146.2 | -0.73 |
| Ireland | 578.95(471.41-700.62) | 15.96(13.00-19.45) | 704.73(551.68-862.94) | 16.08(12.39-20.20) | 0.16(0.08,0.24) | 21.72 | 0.75 |
| Israel | 727.96(557.96-916.47) | 14.84(11.51-18.71) | 1307.93(1022.47-1640.39) | 14.53(11.31-18.35) | -0.05(-0.06,-0.03) | 79.67 | -2.08 |
| Italy | 8097.97(6774.86-9703.72) | 13.86(11.61-16.61) | 6833.59(5726.04-8203.64) | 13.90(11.59-16.69) | 0.01(0.01,0.01) | -15.61 | 0.27 |
| Jamaica | 312.97(236.18-405.58) | 12.58(9.72-15.99) | 391.27(300.82-500.28) | 12.52(9.69-15.79) | -0.02(-0.03,-0.02) | 25.02 | -0.46 |
| Japan | 19211.41(16011.59-22885.79) | 15.41(12.71-18.46) | 15043.47(12447.28-18173.80) | 15.58(12.88-18.76) | 0.22(0.10,0.34) | -21.7 | 1.1 |
| Jordan | 573.67(418.83-756.49) | 14.44(11.00-18.61) | 2004.04(1495.70-2549.67) | 14.30(10.90-18.08) | -0.05(-0.06,-0.04) | 249.34 | -0.94 |
| Kazakhstan | 2165.57(1650.82-2813.83) | 12.47(9.63-16.01) | 2323.37(1799.43-2942.89) | 12.57(9.63-15.97) | 0.03(0.03,0.04) | 7.29 | 0.84 |
| Kenya | 3070.21(2471.61-3723.66) | 14.36(11.99-17.25) | 7635.91(6214.86-9181.52) | 14.27(11.89-17.05) | -0.02(-0.02,-0.01) | 148.71 | -0.68 |
| Kiribati | 12.82(9.66-16.50) | 16.65(13.09-20.86) | 21.08(16.38-26.74) | 16.58(12.98-20.85) | -0.01(-0.02,0.00) | 64.43 | -0.47 |
| Kuwait | 317.31(237.08-411.45) | 14.97(11.36-19.18) | 801.18(625.00-1025.36) | 14.79(11.38-18.86) | -0.06(-0.08,-0.04) | 152.49 | -1.2 |
| Kyrgyzstan | 561.61(425.74-726.18) | 12.38(9.49-15.77) | 870.44(660.30-1112.63) | 12.27(9.36-15.47) | -0.03(-0.04,-0.03) | 54.99 | -0.87 |
| Lao People’s Democratic Republic | 650.01(488.04-825.71) | 16.56(12.72-20.91) | 1370.40(1050.07-1735.93) | 16.77(12.99-21.09) | 0.10(0.07,0.12) | 110.83 | 1.23 |
| Latvia | 303.25(232.79-381.14) | 11.71(8.93-14.85) | 178.55(141.77-225.84) | 11.77(9.02-14.94) | 0.03(0.02,0.04) | -41.12 | 0.56 |
| Lebanon | 436.66(332.58-555.30) | 14.40(11.13-18.04) | 852.92(663.02-1093.79) | 14.34(11.03-18.26) | -0.03(-0.04,-0.02) | 95.33 | -0.43 |
| Lesotho | 183.99(141.43-232.17) | 13.57(10.70-17.10) | 278.84(213.61-356.98) | 13.53(10.59-17.11) | 0.00(-0.00,0.01) | 51.55 | -0.33 |
| Liberia | 328.73(245.83-424.06) | 14.59(11.26-18.51) | 807.32(619.25-1023.99) | 14.46(11.20-18.39) | -0.05(-0.06,-0.04) | 145.59 | -0.85 |
| Libya | 627.10(466.55-818.52) | 14.57(11.25-18.60) | 1119.71(861.21-1415.60) | 14.19(10.83-18.02) | -0.11(-0.12,-0.09) | 78.55 | -2.62 |
| Lithuania | 435.69(336.37-559.96) | 11.66(8.96-14.92) | 273.15(217.39-346.43) | 11.83(9.09-15.18) | 0.03(0.02,0.04) | -37.31 | 1.44 |
| Luxembourg | 58.59(45.60-74.96) | 14.58(11.26-18.44) | 92.20(72.64-115.31) | 14.62(11.26-18.49) | 0.02(0.02,0.03) | 57.37 | 0.28 |
| Madagascar | 1567.56(1189.86-2009.02) | 14.16(11.10-17.75) | 4090.75(3100.86-5274.76) | 14.09(10.89-17.85) | -0.02(-0.02,-0.01) | 160.96 | -0.45 |
| Malawi | 1280.44(962.23-1658.21) | 14.01(11.10-17.78) | 2735.13(2065.12-3529.47) | 13.89(10.97-17.68) | -0.02(-0.03,-0.01) | 113.61 | -0.8 |
| Malaysia | 3275.49(2509.44-4242.73) | 17.41(13.60-22.19) | 6336.95(4889.63-8005.59) | 17.69(13.67-22.36) | 0.10(0.08,0.13) | 93.47 | 1.63 |
| Maldives | 36.70(27.59-47.95) | 17.39(13.60-21.62) | 112.75(87.51-141.67) | 18.02(13.91-22.52) | 0.18(0.14,0.22) | 207.2 | 3.61 |
| Mali | 1091.80(838.11-1385.91) | 14.62(11.48-18.57) | 3169.63(2366.17-4076.66) | 14.53(11.29-18.39) | -0.03(-0.03,-0.02) | 190.31 | -0.65 |
| Malta | 54.32(42.57-68.04) | 14.46(11.15-18.22) | 56.03(43.63-71.23) | 14.54(11.22-18.64) | 0.01(0.00,0.02) | 3.16 | 0.55 |
| Marshall Islands | 7.13(5.36-9.21) | 16.85(13.16-21.19) | 10.29(7.93-13.12) | 16.82(13.02-21.32) | 0.00(-0.01,0.01) | 44.34 | -0.18 |
| Mauritania | 279.30(208.92-359.45) | 14.70(11.47-18.73) | 625.55(475.42-793.53) | 14.70(11.53-18.42) | -0.02(-0.03,-0.01) | 123.97 | -0.04 |
| Mauritius | 217.63(167.40-278.93) | 17.34(13.46-21.87) | 222.15(174.78-277.10) | 17.46(13.73-22.02) | 0.07(0.05,0.10) | 2.08 | 0.71 |
| Mexico | 12590.80(10116.68-15341.20) | 13.92(11.49-16.73) | 19104.73(15711.18-23000.32) | 13.93(11.44-16.78) | -0.01(-0.01,0.00) | 51.74 | 0.07 |
| Micronesia (Federated States of) | 16.56(12.69-21.08) | 16.80(13.10-21.16) | 18.71(14.23-23.85) | 16.87(13.05-21.28) | 0.01(0.00,0.03) | 13.04 | 0.4 |
| Monaco | 4.10(3.23-5.16) | 15.02(11.70-18.98) | 4.35(3.44-5.42) | 15.07(11.66-19.10) | -0.00(-0.02,0.01) | 6.07 | 0.34 |
| Mongolia | 272.64(204.35-350.42) | 12.27(9.45-15.48) | 412.48(324.58-524.59) | 12.35(9.67-15.67) | 0.02(0.01,0.03) | 51.29 | 0.69 |
| Montenegro | 81.85(62.92-104.75) | 12.44(9.57-15.85) | 71.33(55.42-89.92) | 12.45(9.47-15.92) | -0.00(-0.00,0.00) | -12.85 | 0.08 |
| Morocco | 3759.57(2774.73-4843.59) | 14.12(10.88-17.79) | 5439.08(4132.87-6892.84) | 14.01(10.64-17.76) | -0.05(-0.06,-0.04) | 44.67 | -0.82 |
| Mozambique | 1641.81(1271.57-2054.90) | 14.02(11.09-17.65) | 4089.10(3073.26-5273.80) | 13.92(10.94-17.65) | -0.01(-0.02,-0.01) | 149.06 | -0.73 |
| Myanmar | 7091.35(5436.01-9027.35) | 16.45(12.95-20.52) | 9937.25(7750.35-12454.84) | 16.65(13.06-20.80) | 0.10(0.07,0.13) | 40.13 | 1.18 |
| Namibia | 191.59(143.75-248.81) | 13.87(10.88-17.64) | 367.45(278.80-471.97) | 13.87(10.71-17.59) | 0.01(0.01,0.02) | 91.79 | -0.06 |
| Nauru | 1.79(1.37-2.27) | 17.57(13.69-22.16) | 2.03(1.54-2.60) | 17.20(13.21-21.85) | -0.06(-0.10,-0.01) | 13.27 | -2.11 |
| Nepal | 2804.82(2113.89-3598.60) | 14.81(11.40-18.67) | 5018.28(3743.16-6376.23) | 14.33(10.85-18.08) | -0.13(-0.15,-0.12) | 78.92 | -3.21 |
| Netherlands | 3533.38(3028.37-4143.23) | 21.76(18.69-25.47) | 3233.16(2711.50-3860.00) | 21.03(17.37-25.27) | -0.13(-0.17,-0.10) | -8.5 | -3.31 |
| New Zealand | 735.48(603.35-885.53) | 20.38(16.74-24.57) | 990.79(813.95-1195.56) | 20.58(16.89-24.88) | 0.02(0.01,0.03) | 34.71 | 0.98 |
| Nicaragua | 497.06(364.99-642.58) | 13.31(10.17-16.91) | 977.29(743.35-1263.28) | 13.30(10.21-16.98) | -0.01(-0.02,-0.00) | 96.62 | -0.08 |
| Niger | 1003.71(761.34-1277.33) | 14.58(11.51-18.42) | 3120.97(2309.09-3985.12) | 14.48(11.35-18.24) | -0.04(-0.05,-0.03) | 210.94 | -0.68 |
| Nigeria | 13101.46(10713.18-15812.03) | 15.37(12.90-18.38) | 34526.51(28211.77-41511.31) | 15.46(12.94-18.49) | 0.01(0.01,0.02) | 163.53 | 0.6 |
| Niue | 0.37(0.29-0.46) | 17.36(13.46-21.63) | 0.28(0.22-0.35) | 17.45(13.66-22.08) | 0.04(0.03,0.05) | -25.18 | 0.5 |
| North Macedonia | 260.69(197.45-337.08) | 12.44(9.43-16.06) | 265.61(207.06-336.37) | 12.47(9.61-15.94) | -0.00(-0.01,0.00) | 1.89 | 0.27 |
| Northern Mariana Islands | 10.71(8.12-14.03) | 18.43(14.27-23.62) | 8.43(6.65-10.44) | 18.07(14.04-22.72) | -0.07(-0.09,-0.05) | -21.24 | -1.97 |
| Norway | 651.47(545.78-782.31) | 15.09(12.63-18.19) | 759.93(634.97-918.87) | 15.09(12.51-18.20) | 0.00(-0.00,0.01) | 16.65 | 0.01 |
| Oman | 304.31(228.42-393.74) | 14.68(11.27-18.68) | 831.77(634.72-1085.58) | 14.61(11.20-18.69) | -0.03(-0.04,-0.02) | 173.33 | -0.51 |
| Pakistan | 16873.37(13539.74-20453.42) | 15.89(13.00-19.17) | 39702.66(32124.82-48183.42) | 15.53(12.76-18.78) | -0.10(-0.11,-0.10) | 135.3 | -2.26 |
| Palau | 3.05(2.35-3.90) | 17.41(13.62-22.02) | 3.07(2.42-3.76) | 17.69(13.93-22.32) | 0.05(0.03,0.06) | 0.84 | 1.59 |
| Palestine | 282.37(205.66-370.49) | 14.31(10.82-18.29) | 800.93(593.63-1030.16) | 14.15(10.86-17.87) | -0.07(-0.08,-0.06) | 183.65 | -1.15 |
| Panama | 349.97(266.36-447.00) | 13.52(10.47-17.12) | 601.86(458.79-771.48) | 13.65(10.44-17.42) | 0.02(0.02,0.03) | 71.97 | 0.91 |
| Papua New Guinea | 708.51(533.71-909.92) | 16.95(13.34-21.33) | 1868.52(1443.93-2416.56) | 17.05(13.40-21.71) | 0.01(-0.01,0.02) | 163.72 | 0.56 |
| Paraguay | 536.30(404.94-681.12) | 13.45(10.35-17.08) | 1063.52(803.84-1366.96) | 13.48(10.27-17.19) | -0.01(-0.02,-0.00) | 98.31 | 0.21 |
| Peru | 3024.39(2272.24-3917.02) | 13.38(10.28-17.04) | 5262.31(4032.00-6794.38) | 13.45(10.34-17.21) | 0.02(0.01,0.03) | 74 | 0.59 |
| Philippines | 11282.08(9188.24-13571.71) | 17.12(14.35-20.29) | 21174.79(17464.86-25292.53) | 17.14(14.31-20.45) | 0.04(0.02,0.07) | 87.69 | 0.12 |
| Poland | 4715.22(3888.93-5686.83) | 12.60(10.41-15.23) | 4288.48(3538.28-5201.81) | 12.74(10.48-15.39) | 0.03(0.02,0.03) | -9.05 | 1.1 |
| Portugal | 1449.01(1144.18-1811.39) | 14.21(11.19-17.91) | 1294.94(1021.68-1611.33) | 14.28(11.10-18.23) | -0.00(-0.01,0.00) | -10.63 | 0.48 |
| Puerto Rico | 476.13(367.85-603.36) | 12.77(9.90-16.18) | 375.06(293.13-469.11) | 12.79(9.86-16.32) | 0.02(0.01,0.03) | -21.23 | 0.2 |
| Qatar | 87.36(65.75-113.98) | 15.22(11.56-19.51) | 614.50(458.67-808.89) | 15.30(11.78-19.70) | 0.04(0.03,0.05) | 603.38 | 0.49 |
| Republic of Korea | 7706.56(5822.79-9875.84) | 14.41(11.09-18.22) | 6909.00(5428.62-8683.32) | 14.41(11.13-18.48) | 0.00(-0.00,0.01) | -10.35 | -0.02 |
| Republic of Moldova | 517.03(403.04-658.95) | 11.55(8.95-14.80) | 391.73(307.95-495.86) | 11.54(8.85-14.69) | -0.02(-0.02,-0.01) | -24.23 | -0.15 |
| Romania | 2845.53(2206.78-3592.53) | 12.36(9.47-15.71) | 1963.84(1554.42-2470.97) | 12.46(9.64-15.79) | 0.03(0.02,0.03) | -30.99 | 0.8 |
| Russian Federation | 16600.63(13670.45-20078.68) | 10.91(9.01-13.12) | 14842.01(12310.53-17922.09) | 11.75(9.71-14.11) | 0.35(0.28,0.41) | -10.59 | 7.68 |
| Rwanda | 932.34(703.22-1203.75) | 14.14(11.00-17.93) | 1959.10(1499.32-2494.04) | 14.02(11.05-17.75) | -0.01(-0.02,-0.01) | 110.13 | -0.85 |
| Saint Kitts and Nevis | 5.37(4.02-6.86) | 12.58(9.75-15.92) | 7.91(6.16-9.94) | 12.73(9.86-16.17) | 0.02(0.02,0.02) | 47.37 | 1.14 |
| Saint Lucia | 17.84(13.29-23.50) | 12.52(9.60-16.03) | 23.02(17.82-28.97) | 12.57(9.62-15.92) | 0.01(-0.00,0.01) | 28.99 | 0.37 |
| Saint Vincent and the Grenadines | 14.36(10.50-18.74) | 12.50(9.50-15.80) | 14.16(11.03-17.95) | 12.43(9.70-15.83) | -0.01(-0.02,-0.01) | -1.42 | -0.54 |
| Samoa | 29.06(21.77-37.56) | 17.19(13.44-21.69) | 35.94(27.95-45.50) | 17.22(13.44-21.75) | 0.01(-0.00,0.01) | 23.7 | 0.14 |
| San Marino | 3.71(2.88-4.71) | 14.65(11.51-18.42) | 4.03(3.18-5.07) | 14.55(11.25-18.57) | -0.02(-0.02,-0.01) | 8.43 | -0.7 |
| Sao Tome and Principe | 15.75(11.79-20.15) | 14.76(11.44-18.62) | 33.62(25.71-42.50) | 14.70(11.49-18.50) | -0.02(-0.03,-0.02) | 113.48 | -0.41 |
| Saudi Arabia | 2480.84(1821.87-3178.11) | 14.62(11.18-18.51) | 6850.34(5282.81-8653.71) | 14.54(11.19-18.29) | -0.03(-0.05,-0.02) | 176.13 | -0.57 |
| Senegal | 1001.94(750.23-1279.13) | 14.69(11.41-18.59) | 2333.43(1770.36-2976.55) | 14.58(11.47-18.38) | -0.02(-0.03,-0.01) | 132.89 | -0.76 |
| Serbia | 1200.39(929.29-1520.25) | 12.47(9.50-15.86) | 1022.66(797.19-1289.18) | 12.44(9.52-15.94) | -0.00(-0.01,0.00) | -14.81 | -0.22 |
| Seychelles | 13.86(10.48-17.72) | 17.45(13.42-22.01) | 18.50(14.49-22.97) | 17.68(13.64-22.24) | 0.08(0.06,0.10) | 33.53 | 1.32 |
| Sierra Leone | 577.57(438.31-739.26) | 14.64(11.51-18.35) | 1322.52(995.45-1697.64) | 14.46(11.23-18.34) | -0.05(-0.06,-0.04) | 128.98 | -1.24 |
| Singapore | 542.47(411.49-705.54) | 13.99(10.81-17.78) | 773.93(599.71-986.86) | 14.07(10.90-17.78) | -0.00(-0.01,0.01) | 42.67 | 0.57 |
| Slovakia | 662.15(505.85-848.38) | 12.46(9.50-15.99) | 610.23(476.83-767.25) | 12.60(9.62-16.10) | 0.03(0.02,0.04) | -7.84 | 1.13 |
| Slovenia | 255.71(197.40-327.81) | 12.57(9.67-16.16) | 209.93(164.26-263.00) | 12.71(9.67-16.36) | 0.01(0.01,0.02) | -17.9 | 1.13 |
| Solomon Islands | 53.59(40.33-68.81) | 16.67(13.04-20.83) | 116.66(88.39-147.42) | 16.65(12.92-21.00) | -0.00(-0.02,0.01) | 117.72 | -0.13 |
| Somalia | 983.31(754.30-1250.60) | 14.06(10.98-17.69) | 2774.72(2105.88-3537.50) | 13.96(11.03-17.60) | -0.02(-0.03,-0.01) | 182.18 | -0.7 |
| South Africa | 5522.80(4506.98-6694.16) | 14.02(11.63-16.90) | 8716.43(7158.20-10522.52) | 13.95(11.51-16.76) | -0.01(-0.01,-0.00) | 57.83 | -0.5 |
| South Sudan | 802.31(604.53-1023.43) | 14.12(11.08-17.73) | 1253.29(949.76-1606.86) | 14.07(10.98-17.85) | -0.00(-0.01,0.01) | 56.21 | -0.34 |
| Spain | 5572.43(5038.02-6166.06) | 14.02(12.66-15.50) | 5624.14(4892.40-6344.51) | 14.37(12.90-16.00) | 0.05(0.02,0.08) | 0.93 | 2.53 |
| Sri Lanka | 3233.39(2476.25-4114.22) | 17.12(13.39-21.48) | 3797.83(3001.56-4754.96) | 17.27(13.49-21.85) | 0.07(0.04,0.11) | 17.46 | 0.85 |
| Sudan | 2698.36(2012.10-3529.21) | 13.88(10.75-17.76) | 6501.48(4854.47-8410.37) | 13.78(10.59-17.37) | -0.05(-0.06,-0.03) | 140.94 | -0.73 |
| Suriname | 45.16(38.42-52.44) | 10.80(9.30-12.50) | 62.83(54.78-72.39) | 10.77(9.37-12.46) | -0.02(-0.02,-0.02) | 39.14 | -0.29 |
| Sweden | 1266.12(1071.08-1513.57) | 15.03(12.87-17.77) | 1439.70(1208.67-1759.04) | 15.23(12.93-18.46) | 0.01(-0.01,0.03) | 13.71 | 1.37 |
| Switzerland | 1067.87(826.46-1361.96) | 14.72(11.36-18.60) | 1175.90(935.89-1476.06) | 14.63(11.43-18.64) | -0.01(-0.02,0.01) | 10.12 | -0.63 |
| Syrian Arab Republic | 1746.24(1281.38-2264.48) | 14.19(10.99-18.01) | 1923.29(1470.34-2521.88) | 13.90(10.61-17.51) | -0.08(-0.10,-0.07) | 10.14 | -2.06 |
| Taiwan (Province of China) | 4024.09(3144.69-5051.16) | 17.42(13.78-21.57) | 4077.57(3223.84-4989.26) | 18.36(14.34-23.01) | 0.12(0.09,0.14) | 1.33 | 5.35 |
| Tajikistan | 658.84(484.26-844.60) | 12.41(9.51-15.79) | 1317.17(990.94-1689.02) | 12.29(9.40-15.64) | -0.04(-0.05,-0.03) | 99.92 | -0.92 |
| Thailand | 11273.51(8395.81-14344.02) | 17.05(13.14-21.55) | 10657.67(8369.33-13122.33) | 17.19(13.35-21.64) | 0.08(0.05,0.10) | -5.46 | 0.78 |
| Timor-Leste | 124.80(94.66-158.46) | 15.64(12.17-19.56) | 230.09(171.17-297.23) | 15.60(12.17-19.42) | 0.06(0.03,0.08) | 84.36 | -0.28 |
| Togo | 487.33(363.81-622.51) | 14.58(11.36-18.42) | 1219.40(934.95-1537.70) | 14.47(11.33-18.26) | -0.02(-0.03,-0.01) | 150.22 | -0.81 |
| Tokelau | 0.25(0.19-0.32) | 17.03(13.44-21.71) | 0.23(0.18-0.29) | 17.25(13.45-21.85) | 0.05(0.04,0.06) | -8.32 | 1.27 |
| Tonga | 16.37(12.37-21.27) | 17.20(13.47-21.76) | 17.65(13.35-22.41) | 17.27(13.33-21.77) | 0.01(-0.00,0.02) | 7.83 | 0.39 |
| Trinidad and Tobago | 123.12(112.99-136.90) | 9.72(8.92-10.77) | 168.35(133.75-209.99) | 12.59(9.82-15.79) | 0.59(0.33,0.85) | 36.74 | 29.55 |
| Tunisia | 1273.58(942.56-1650.54) | 14.35(10.84-18.28) | 1651.64(1281.36-2088.85) | 14.22(10.78-18.06) | -0.04(-0.05,-0.04) | 29.68 | -0.87 |
| Turkey | 8642.26(7101.71-10504.58) | 14.00(11.61-16.98) | 12006.88(10019.19-14671.08) | 13.96(11.61-17.06) | -0.03(-0.03,-0.02) | 38.93 | -0.24 |
| Turkmenistan | 476.88(355.76-619.53) | 12.44(9.63-15.96) | 686.98(522.03-872.46) | 12.58(9.61-15.88) | 0.03(0.01,0.04) | 44.06 | 1.16 |
| Tuvalu | 1.55(1.19-1.97) | 16.66(13.00-21.15) | 2.19(1.69-2.75) | 16.95(13.10-21.22) | 0.06(0.05,0.06) | 41.6 | 1.73 |
| Uganda | 2165.34(1628.15-2771.98) | 14.00(11.01-17.76) | 5802.25(4331.61-7442.80) | 13.93(10.87-17.45) | -0.01(-0.02,-0.01) | 167.96 | -0.51 |
| Ukraine | 5997.85(4872.41-7291.90) | 11.80(9.72-14.33) | 4478.50(3698.94-5492.64) | 11.71(9.62-14.20) | -0.03(-0.03,-0.02) | -25.33 | -0.73 |
| United Arab Emirates | 357.33(266.02-461.93) | 15.27(11.61-19.33) | 1483.38(1117.87-1942.28) | 14.80(11.22-18.89) | -0.10(-0.11,-0.09) | 315.13 | -3.05 |
| United Kingdom | 7063.22(5938.56-8474.17) | 12.38(10.41-14.71) | 6888.37(5834.76-8315.77) | 11.23(9.45-13.45) | -0.25(-0.36,-0.14) | -2.48 | -9.28 |
| United Republic of Tanzania | 3320.92(2498.47-4325.53) | 14.08(10.96-17.81) | 8117.52(6228.64-10304.95) | 14.01(11.00-17.66) | 0.00(-0.01,0.01) | 144.44 | -0.53 |
| United States Virgin Islands | 13.60(10.52-17.06) | 12.71(9.73-16.20) | 8.72(6.92-10.98) | 12.85(9.86-16.47) | 0.03(0.03,0.04) | -35.87 | 1.07 |
| United States of America | 47066.72(38456.19-57258.28) | 17.93(14.64-21.63) | 50622.20(41824.18-60838.44) | 17.05(13.90-20.52) | -0.11(-0.17,-0.05) | 7.55 | -4.9 |
| Uruguay | 461.36(354.74-590.14) | 15.25(11.70-19.60) | 498.88(386.67-631.87) | 15.24(11.74-19.38) | -0.02(-0.03,-0.01) | 8.13 | -0.08 |
| Uzbekistan | 2641.86(1964.91-3432.80) | 12.37(9.54-15.70) | 4448.64(3405.79-5699.46) | 12.36(9.44-15.72) | -0.01(-0.02,-0.00) | 68.39 | -0.1 |
| Vanuatu | 24.66(18.43-31.88) | 16.81(12.99-21.20) | 54.02(41.20-69.15) | 16.82(13.06-21.36) | 0.01(-0.01,0.02) | 119.05 | 0.06 |
| Venezuela (Bolivarian Republic of) | 2728.87(2039.81-3523.82) | 13.50(10.38-17.26) | 3366.60(2631.78-4209.37) | 13.30(10.19-16.83) | -0.03(-0.04,-0.02) | 23.37 | -1.48 |
| Viet Nam | 13487.89(10177.97-17096.86) | 18.70(14.59-23.29) | 18898.28(14922.67-23487.42) | 18.96(14.88-23.53) | 0.09(0.07,0.11) | 40.11 | 1.4 |
| Yemen | 1620.87(1203.74-2073.85) | 13.89(10.68-17.78) | 4713.84(3575.19-6005.35) | 13.61(10.51-17.27) | -0.08(-0.08,-0.07) | 190.82 | -2 |
| Zambia | 1029.97(767.07-1334.77) | 14.01(10.87-17.88) | 2784.21(2105.33-3568.13) | 13.97(10.90-17.82) | -0.01(-0.01,0.00) | 170.32 | -0.29 |
| Zimbabwe | 1318.99(983.89-1708.67) | 13.70(10.59-17.32) | 2128.17(1621.23-2724.26) | 13.52(10.56-17.15) | -0.04(-0.05,-0.03) | 61.35 | -1.26 |

Abbreviations: ASR, age-standardized rate; RC, relative change; EAPC, estimated annual percentage change; SDI, sociodemographic index; UI, uncertainty interval; CI, confidence interval.

**Supplemental Tables S5** The number and ASR of prevalence of Schizophrenia burden for the 204 countries/regions.

| Location | 1990 Case number  (95% UI) | 1990 ASR  (95% UI) | 2021 Case number  (95% UI) | 2021 ASR  (95% UI) | EAPCs  (95%, CI) | RC of numbers (%) | RC of ASR (%) |
| --- | --- | --- | --- | --- | --- | --- | --- |
| Afghanistan | 15403.44(11992.55-19136.84) | 221.31(173.56-280.24) | 49198.90(36930.82-64346.49) | 218.33(168.88-278.40) | -0.02(-0.06,0.01) | 219.4 | -1.35 |
| Albania | 6498.55(4976.90-8438.41) | 216.51(168.48-276.96) | 6848.66(5395.63-8591.43) | 221.34(173.40-281.90) | 0.08(0.07,0.08) | 5.39 | 2.23 |
| Algeria | 47256.66(35918.89-60348.61) | 248.48(195.62-311.92) | 113232.79(88122.82-144217.43) | 246.67(193.01-312.21) | -0.02(-0.02,-0.01) | 139.61 | -0.73 |
| American Samoa | 120.85(91.34-156.12) | 301.29(230.44-378.27) | 145.28(112.25-181.36) | 292.21(223.19-367.78) | -0.08(-0.09,-0.07) | 20.22 | -3.01 |
| Andorra | 178.62(138.21-225.01) | 273.82(214.92-340.96) | 311.34(248.33-389.35) | 271.42(212.91-343.81) | -0.03(-0.04,-0.02) | 74.31 | -0.87 |
| Angola | 16374.27(12168.26-21267.79) | 218.91(170.22-276.26) | 52167.25(39189.38-67970.47) | 223.96(172.96-283.56) | 0.12(0.09,0.14) | 218.59 | 2.31 |
| Antigua and Barbuda | 131.58(100.30-170.55) | 233.05(179.67-297.62) | 247.16(191.98-312.04) | 234.12(180.95-295.93) | 0.02(0.01,0.03) | 87.84 | 0.46 |
| Argentina | 88382.77(68829.99-111523.63) | 277.33(215.92-350.51) | 138109.81(107232.34-172804.69) | 277.59(215.91-348.36) | -0.01(-0.01,0.00) | 56.26 | 0.09 |
| Armenia | 7354.69(5641.27-9423.22) | 217.70(168.26-277.18) | 7700.20(6065.19-9693.32) | 220.29(170.76-279.37) | 0.06(0.05,0.07) | 4.7 | 1.19 |
| Australia | 71416.47(65728.11-76937.77) | 388.26(357.60-418.49) | 112864.25(103874.68-121734.63) | 388.26(358.09-419.41) | 0.01(0.00,0.01) | 58.04 | 0 |
| Austria | 24008.54(19041.24-29952.64) | 265.94(211.22-335.58) | 29477.45(23378.79-36374.68) | 266.03(210.06-335.72) | -0.00(-0.01,0.00) | 22.78 | 0.03 |
| Azerbaijan | 14537.92(11210.62-18909.29) | 217.23(168.91-276.64) | 27071.50(20931.09-34459.92) | 220.74(170.56-279.96) | 0.10(0.06,0.14) | 86.21 | 1.62 |
| Bahamas | 556.26(424.41-726.09) | 233.08(180.32-295.86) | 1017.77(785.37-1281.36) | 230.51(178.16-291.77) | -0.02(-0.03,-0.02) | 82.97 | -1.1 |
| Bahrain | 1376.19(1015.90-1819.96) | 261.89(205.00-334.29) | 5084.49(3830.64-6484.52) | 263.00(202.71-332.55) | 0.02(0.01,0.02) | 269.46 | 0.42 |
| Bangladesh | 229498.61(173870.79-293277.16) | 290.91(225.82-365.54) | 475978.06(365129.81-600223.91) | 285.29(220.53-358.33) | -0.08(-0.09,-0.08) | 107.4 | -1.93 |
| Barbados | 676.86(553.09-811.92) | 261.71(214.71-313.41) | 887.61(696.89-1101.38) | 248.17(190.49-310.26) | -0.06(-0.09,-0.02) | 31.14 | -5.18 |
| Belarus | 23205.03(17879.97-29220.51) | 202.10(154.91-255.59) | 23199.91(18197.73-28956.14) | 205.11(157.88-260.44) | 0.07(0.05,0.08) | -0.02 | 1.49 |
| Belgium | 30797.23(24582.36-38292.75) | 265.03(208.10-333.61) | 35843.94(28869.18-44609.01) | 264.11(210.54-336.65) | -0.01(-0.01,0.00) | 16.39 | -0.34 |
| Belize | 295.04(223.62-385.47) | 220.74(172.68-280.31) | 931.93(725.45-1177.27) | 221.32(173.02-277.39) | -0.00(-0.01,0.00) | 215.87 | 0.26 |
| Benin | 7347.27(5526.72-9524.05) | 228.87(178.14-287.16) | 22504.99(17114.93-29239.00) | 229.89(178.50-289.65) | 0.01(0.00,0.02) | 206.3 | 0.44 |
| Bermuda | 169.35(130.45-216.69) | 242.84(187.73-308.01) | 194.13(152.40-243.58) | 245.34(190.62-310.65) | 0.05(0.04,0.06) | 14.63 | 1.03 |
| Bhutan | 1374.59(1041.84-1757.55) | 293.41(226.56-369.17) | 2408.95(1830.56-3058.71) | 296.32(229.94-372.69) | 0.02(0.02,0.02) | 75.25 | 0.99 |
| Bolivia (Plurinational State of) | 11382.08(8767.90-14568.36) | 230.18(182.52-290.37) | 27364.38(21212.68-35289.24) | 233.83(182.47-298.95) | 0.04(0.03,0.05) | 140.42 | 1.59 |
| Bosnia and Herzegovina | 10387.83(8076.81-13253.40) | 212.37(165.37-270.49) | 8825.50(6958.37-10971.96) | 221.61(171.21-280.54) | 0.14(0.12,0.17) | -15.04 | 4.35 |
| Botswana | 2062.08(1577.48-2664.20) | 219.15(170.95-277.74) | 5638.03(4350.65-7242.64) | 228.13(179.36-288.15) | 0.14(0.12,0.15) | 173.41 | 4.1 |
| Brazil | 318174.22(260159.79-382515.35) | 241.00(199.86-286.74) | 607170.58(504437.66-720997.24) | 243.37(202.19-289.75) | 0.04(0.03,0.05) | 90.83 | 0.98 |
| Brunei Darussalam | 625.78(471.36-821.40) | 265.08(209.74-341.89) | 1393.57(1098.88-1757.55) | 259.64(206.53-324.95) | -0.04(-0.05,-0.04) | 122.7 | -2.05 |
| Bulgaria | 21327.98(16798.98-26585.68) | 215.35(168.24-273.77) | 18056.74(14491.28-22424.87) | 218.27(170.37-276.79) | 0.04(0.03,0.06) | -15.34 | 1.36 |
| Burkina Faso | 14030.74(10858.04-18026.03) | 222.34(176.52-283.74) | 36564.47(27871.28-47400.97) | 223.39(176.90-284.44) | 0.01(0.00,0.01) | 160.6 | 0.47 |
| Burundi | 8303.93(6271.52-10948.54) | 212.07(166.93-272.57) | 20557.54(15881.16-26625.97) | 206.60(161.70-263.86) | -0.07(-0.08,-0.07) | 147.56 | -2.58 |
| Cabo Verde | 576.22(435.83-753.04) | 239.41(185.96-306.32) | 1440.77(1108.85-1849.74) | 240.85(188.38-304.82) | 0.03(0.02,0.03) | 150.04 | 0.6 |
| Cambodia | 19508.97(14780.59-25016.44) | 256.02(197.19-321.16) | 45820.42(34575.45-58744.58) | 267.19(202.81-338.15) | 0.19(0.15,0.23) | 134.87 | 4.36 |
| Cameroon | 16793.59(12771.21-21896.23) | 228.55(179.86-292.75) | 55714.88(42426.64-72421.41) | 226.55(176.71-286.17) | -0.02(-0.03,-0.01) | 231.76 | -0.88 |
| Canada | 89321.36(88195.02-90355.92) | 287.97(284.41-291.36) | 125956.11(124158.32-127804.50) | 288.17(284.43-291.67) | 0.00(0.00,0.00) | 41.01 | 0.07 |
| Central African Republic | 4133.72(3136.24-5400.45) | 203.85(157.98-259.57) | 8638.06(6559.12-11174.86) | 199.29(154.55-254.65) | -0.07(-0.08,-0.06) | 108.97 | -2.24 |
| Chad | 9000.58(6948.55-11704.30) | 222.87(174.65-282.02) | 25096.62(19055.07-32501.80) | 224.75(175.71-283.57) | 0.04(0.04,0.05) | 178.83 | 0.85 |
| Chile | 35646.17(27651.57-45414.81) | 278.16(218.18-351.22) | 61024.18(47993.00-77295.72) | 281.45(219.50-357.28) | 0.04(0.03,0.05) | 71.19 | 1.18 |
| China | 3558618.53(3076266.50-4080968.01) | 300.81(260.98-343.19) | 5322429.61(4637002.61-6043639.53) | 312.36(271.69-356.39) | 0.07(0.04,0.10) | 49.56 | 3.84 |
| Colombia | 67545.19(51867.50-86876.96) | 240.12(187.67-302.78) | 131170.14(102534.37-166332.58) | 242.00(189.15-307.48) | 0.03(0.02,0.04) | 94.2 | 0.78 |
| Comoros | 729.54(551.41-947.13) | 226.13(176.33-285.82) | 1582.06(1229.30-2052.54) | 224.19(176.23-288.35) | -0.02(-0.02,-0.01) | 116.86 | -0.86 |
| Congo | 3807.25(2905.91-4902.52) | 218.91(169.82-274.20) | 10740.26(8366.53-13749.69) | 219.91(173.85-279.04) | 0.04(0.03,0.06) | 182.1 | 0.46 |
| Cook Islands | 50.18(38.10-63.33) | 300.78(228.53-376.60) | 58.69(45.82-72.85) | 306.12(235.53-385.58) | 0.05(0.04,0.06) | 16.95 | 1.78 |
| Costa Rica | 6360.09(4882.24-8251.63) | 244.28(193.17-308.11) | 13177.14(10325.27-16659.88) | 246.45(192.70-311.86) | 0.03(0.02,0.04) | 107.18 | 0.89 |
| Croatia | 12544.73(9813.55-15859.11) | 222.70(172.55-284.45) | 11356.92(8939.91-14135.80) | 225.66(174.53-285.33) | 0.05(0.04,0.05) | -9.47 | 1.33 |
| Cuba | 26018.56(20059.06-33034.56) | 230.73(178.59-290.55) | 31151.26(24613.83-38799.76) | 228.78(179.41-285.66) | 0.00(-0.02,0.02) | 19.73 | -0.84 |
| Cyprus | 2176.92(1728.58-2723.12) | 265.24(210.48-331.63) | 4533.48(3576.53-5682.36) | 265.80(209.55-332.88) | -0.01(-0.02,-0.00) | 108.25 | 0.21 |
| Czechia | 25869.11(20267.71-32776.40) | 225.99(174.16-287.99) | 29111.55(23256.15-36251.28) | 228.86(176.90-292.22) | 0.03(0.02,0.03) | 12.53 | 1.27 |
| C么te d'Ivoire | 19598.44(14848.82-25821.31) | 225.57(174.34-287.44) | 51009.06(38651.38-66047.85) | 226.54(175.51-286.63) | -0.01(-0.02,0.00) | 160.27 | 0.43 |
| Democratic People's Republic of Korea | 56819.99(43946.47-72013.96) | 273.55(212.49-343.80) | 82644.61(64323.38-103892.60) | 262.50(204.38-330.74) | -0.17(-0.18,-0.15) | 45.45 | -4.04 |
| Democratic Republic of the Congo | 57984.08(44482.53-75327.57) | 215.14(168.35-273.46) | 145658.96(110904.61-187082.56) | 208.48(161.09-260.70) | -0.10(-0.13,-0.06) | 151.21 | -3.1 |
| Denmark | 14948.53(12510.05-17762.78) | 247.07(205.26-295.20) | 24415.33(21793.75-26762.23) | 362.11(322.98-394.80) | 1.74(1.41,2.08) | 63.33 | 46.56 |
| Djibouti | 705.10(529.31-915.81) | 224.36(174.70-282.30) | 2759.86(2118.15-3538.17) | 223.16(174.25-280.99) | -0.00(-0.01,0.01) | 291.41 | -0.54 |
| Dominica | 138.79(107.04-177.77) | 224.12(174.36-283.46) | 169.57(132.91-210.81) | 225.23(175.82-282.80) | 0.02(0.01,0.03) | 22.17 | 0.5 |
| Dominican Republic | 12905.94(9720.17-16742.06) | 221.18(171.57-278.39) | 25873.23(20061.32-32922.76) | 227.74(178.23-288.08) | 0.10(0.09,0.11) | 100.48 | 2.96 |
| Ecuador | 19211.33(14689.44-25081.35) | 237.39(184.21-302.20) | 43346.00(34249.84-55004.20) | 238.35(189.57-301.18) | 0.03(0.02,0.04) | 125.63 | 0.4 |
| Egypt | 109644.58(83881.22-140796.57) | 240.16(187.62-304.94) | 238661.35(182299.02-303932.60) | 242.74(187.98-305.36) | 0.03(0.03,0.04) | 117.67 | 1.07 |
| El Salvador | 9319.65(7142.27-11931.75) | 228.58(179.62-287.14) | 14677.39(11473.92-18572.29) | 230.92(181.04-291.79) | 0.04(0.03,0.04) | 57.49 | 1.02 |
| Equatorial Guinea | 639.85(494.93-830.34) | 213.91(167.26-271.07) | 2843.96(2157.20-3679.32) | 234.44(182.37-297.44) | 0.44(0.36,0.52) | 344.47 | 9.6 |
| Eritrea | 5126.93(3904.58-6595.85) | 212.99(167.02-267.76) | 12141.36(9197.37-15627.79) | 214.82(166.39-272.08) | 0.02(-0.00,0.04) | 136.82 | 0.86 |
| Estonia | 3559.08(2813.74-4477.30) | 204.51(159.86-258.41) | 3231.11(2577.24-4092.32) | 210.26(163.64-269.16) | 0.10(0.09,0.11) | -9.22 | 2.81 |
| Eswatini | 1172.05(883.17-1516.36) | 217.43(167.93-277.92) | 2260.46(1707.17-2936.32) | 220.88(171.08-279.18) | 0.06(0.05,0.07) | 92.86 | 1.59 |
| Ethiopia | 76262.76(62342.18-92089.19) | 218.90(182.54-260.37) | 189549.75(154158.25-229566.58) | 222.73(184.02-265.41) | 0.08(0.06,0.09) | 148.55 | 1.75 |
| Fiji | 1888.80(1417.43-2402.81) | 283.39(217.53-354.05) | 2692.70(2096.17-3405.83) | 287.05(223.84-362.22) | 0.04(0.03,0.06) | 42.56 | 1.29 |
| Finland | 16472.24(13654.96-19502.90) | 277.60(227.97-330.05) | 17414.86(13919.43-21402.65) | 263.17(208.82-326.82) | -0.18(-0.23,-0.13) | 5.72 | -5.2 |
| France | 168054.25(133665.46-209082.26) | 260.27(205.29-325.53) | 197666.03(158868.26-247061.82) | 259.46(202.48-330.58) | -0.02(-0.03,-0.01) | 17.62 | -0.31 |
| Gabon | 1745.56(1308.45-2235.59) | 231.28(177.78-290.73) | 3747.30(2847.14-4754.36) | 231.71(177.83-291.01) | 0.01(0.00,0.01) | 114.68 | 0.19 |
| Gambia | 1557.72(1176.41-2005.94) | 226.60(176.43-284.50) | 4162.01(3150.11-5339.74) | 225.61(175.70-282.71) | -0.01(-0.02,0.00) | 167.19 | -0.44 |
| Georgia | 12809.49(10047.74-16187.10) | 218.35(170.46-277.15) | 8879.19(6970.41-11210.61) | 218.04(169.05-275.47) | 0.03(-0.00,0.06) | -30.68 | -0.14 |
| Germany | 235239.14(188844.36-290967.79) | 243.43(194.11-303.90) | 253729.96(203539.05-312553.25) | 244.30(192.46-309.41) | -0.01(-0.03,0.00) | 7.86 | 0.36 |
| Ghana | 25137.39(18877.35-32331.23) | 227.79(175.87-287.27) | 68854.57(51939.66-88752.58) | 233.13(181.22-295.36) | 0.09(0.08,0.10) | 173.91 | 2.34 |
| Greece | 31212.91(24935.48-38294.44) | 263.78(210.23-328.67) | 32757.17(26281.00-40764.87) | 262.73(203.53-329.52) | -0.02(-0.03,-0.02) | 4.95 | -0.4 |
| Greenland | 204.77(154.80-261.25) | 332.26(256.60-416.05) | 214.73(168.38-267.10) | 331.14(257.22-412.81) | 0.07(0.03,0.11) | 4.87 | -0.34 |
| Grenada | 153.43(118.47-196.90) | 222.24(174.66-281.19) | 261.73(204.18-325.98) | 227.87(177.32-286.02) | 0.08(0.07,0.08) | 70.59 | 2.53 |
| Guam | 427.41(322.26-557.06) | 316.69(244.19-403.18) | 533.61(418.21-668.45) | 312.78(241.27-398.78) | -0.03(-0.04,-0.02) | 24.85 | -1.23 |
| Guatemala | 12938.89(9897.26-16740.15) | 227.55(178.58-288.65) | 33428.56(25908.50-42766.06) | 229.07(180.83-288.28) | 0.01(0.01,0.02) | 158.36 | 0.67 |
| Guinea | 9949.13(7668.81-12674.09) | 226.68(176.78-284.11) | 22080.03(16673.93-28246.67) | 226.01(175.94-282.30) | -0.02(-0.03,-0.00) | 121.93 | -0.3 |
| Guinea-Bissau | 1519.10(1152.40-1992.02) | 220.91(172.37-279.86) | 3475.82(2636.43-4472.47) | 221.07(174.53-282.03) | -0.02(-0.03,-0.01) | 128.81 | 0.07 |
| Guyana | 1361.23(1031.39-1767.95) | 211.92(165.34-268.14) | 1676.72(1324.23-2121.19) | 217.19(172.20-273.53) | 0.07(0.06,0.07) | 23.18 | 2.49 |
| Haiti | 10190.92(7891.95-13154.94) | 205.78(161.65-260.26) | 24156.97(18525.22-30965.64) | 201.67(158.59-254.15) | -0.07(-0.08,-0.06) | 137.04 | -2 |
| Honduras | 7453.26(5673.15-9520.79) | 229.03(180.57-287.00) | 21172.49(16262.66-26932.42) | 228.12(179.46-286.02) | -0.02(-0.02,-0.01) | 184.07 | -0.4 |
| Hungary | 25768.81(20225.23-32478.94) | 219.10(170.24-279.03) | 25985.44(20781.31-32282.42) | 223.76(173.26-284.04) | 0.06(0.05,0.06) | 0.84 | 2.13 |
| Iceland | 707.41(550.61-882.90) | 268.87(208.97-334.67) | 1085.45(870.07-1351.80) | 269.02(212.94-338.99) | -0.00(-0.01,0.00) | 53.44 | 0.06 |
| India | 2083452.89(1715455.32-2473527.65) | 286.49(238.00-336.77) | 4373183.19(3639071.06-5178753.17) | 296.22(246.51-349.92) | 0.13(0.09,0.17) | 109.9 | 3.4 |
| Indonesia | 452063.13(367907.97-540857.98) | 278.85(231.14-331.58) | 888192.92(736988.99-1054079.73) | 286.49(238.10-339.27) | 0.13(0.10,0.17) | 96.48 | 2.74 |
| Iran (Islamic Republic of) | 104416.17(85018.62-125708.38) | 248.75(206.29-296.42) | 250268.80(206658.59-296635.26) | 250.71(207.03-297.11) | 0.03(0.02,0.03) | 139.68 | 0.79 |
| Iraq | 32396.62(24106.68-41705.47) | 243.67(188.79-308.36) | 95671.34(72994.05-122570.35) | 244.12(189.02-307.12) | 0.04(0.02,0.06) | 195.31 | 0.19 |
| Ireland | 11518.68(9427.70-13788.27) | 323.25(264.38-387.09) | 18282.94(14179.72-22576.23) | 322.51(246.67-403.06) | 0.13(0.04,0.21) | 58.72 | -0.23 |
| Israel | 12631.89(10045.92-15758.62) | 271.97(216.76-339.12) | 25804.24(20396.58-32250.91) | 266.62(210.03-335.44) | -0.04(-0.06,-0.03) | 104.28 | -1.97 |
| Italy | 168048.84(142984.61-195423.73) | 251.79(212.09-295.32) | 186299.24(160315.28-215635.59) | 251.90(212.48-296.66) | -0.00(-0.01,-0.00) | 10.86 | 0.04 |
| Jamaica | 4474.92(3431.10-5702.48) | 226.43(175.41-285.45) | 7043.20(5455.62-8881.23) | 226.30(175.34-284.91) | -0.01(-0.02,-0.01) | 57.39 | -0.06 |
| Japan | 416434.74(348737.47-487572.20) | 283.33(236.55-333.33) | 409399.01(348029.27-474911.36) | 279.02(232.22-332.33) | 0.11(0.00,0.21) | -1.69 | -1.52 |
| Jordan | 6690.32(5008.78-8784.66) | 249.47(194.15-317.97) | 30679.86(23551.64-39264.33) | 248.82(194.86-314.85) | -0.01(-0.02,-0.00) | 358.57 | -0.26 |
| Kazakhstan | 34017.80(26324.75-43740.84) | 215.39(166.94-274.21) | 44321.90(34717.05-56438.72) | 220.75(173.13-279.94) | 0.11(0.09,0.13) | 30.29 | 2.49 |
| Kenya | 33831.67(27558.12-41004.68) | 222.05(184.60-264.39) | 94215.73(77156.37-113435.81) | 223.14(185.70-265.81) | 0.02(0.01,0.02) | 178.48 | 0.49 |
| Kiribati | 161.33(121.33-209.15) | 260.80(199.98-330.73) | 289.20(221.13-367.29) | 256.57(197.84-325.81) | -0.05(-0.06,-0.04) | 79.26 | -1.62 |
| Kuwait | 4734.27(3446.16-6228.99) | 268.32(204.31-339.13) | 16560.80(12597.32-21280.18) | 265.73(206.19-337.41) | -0.04(-0.05,-0.02) | 249.81 | -0.97 |
| Kyrgyzstan | 7938.60(6158.44-10171.95) | 211.19(164.13-266.44) | 13860.55(10643.41-17767.58) | 208.81(162.23-265.38) | -0.03(-0.05,-0.01) | 74.6 | -1.13 |
| Lao People's Democratic Republic | 8209.24(6129.44-10526.99) | 260.13(198.67-329.35) | 19871.43(15202.59-24995.56) | 273.30(212.26-340.14) | 0.22(0.19,0.26) | 142.06 | 5.06 |
| Latvia | 6052.93(4715.36-7683.00) | 203.72(158.80-260.39) | 4570.12(3664.94-5643.27) | 207.79(160.53-263.33) | 0.09(0.08,0.10) | -24.5 | 2 |
| Lebanon | 6666.76(5232.10-8464.63) | 250.21(195.11-316.08) | 15639.22(12011.07-19916.86) | 250.49(193.78-317.83) | 0.00(-0.00,0.01) | 134.58 | 0.11 |
| Lesotho | 2312.01(1795.32-2936.34) | 203.73(160.68-256.54) | 3527.87(2670.02-4552.75) | 209.16(163.39-264.62) | 0.10(0.09,0.11) | 52.59 | 2.67 |
| Liberia | 3980.74(2995.41-5210.40) | 224.06(172.76-286.52) | 9906.48(7582.86-12705.31) | 220.25(171.04-278.02) | -0.02(-0.04,-0.01) | 148.86 | -1.7 |
| Libya | 7986.38(5953.77-10346.15) | 256.40(196.52-323.54) | 19835.98(15176.30-25346.26) | 245.85(188.86-312.40) | -0.15(-0.17,-0.12) | 148.37 | -4.12 |
| Lithuania | 8160.60(6294.68-10249.24) | 203.35(156.98-256.39) | 6748.40(5409.57-8529.89) | 209.51(164.07-264.29) | 0.10(0.09,0.11) | -17.31 | 3.03 |
| Luxembourg | 1235.75(973.75-1549.66) | 269.27(211.31-336.47) | 2192.17(1736.61-2745.30) | 271.58(211.27-341.73) | 0.03(0.02,0.03) | 77.4 | 0.86 |
| Madagascar | 18428.27(14026.01-23841.53) | 218.32(171.23-273.68) | 49223.87(37277.75-63697.19) | 217.68(169.13-274.60) | -0.00(-0.01,0.01) | 167.11 | -0.29 |
| Malawi | 14285.02(10846.84-18669.02) | 207.72(162.25-266.19) | 30257.08(22786.00-39389.65) | 206.77(161.15-264.04) | 0.01(-0.00,0.02) | 111.81 | -0.46 |
| Malaysia | 45291.14(34352.88-58899.94) | 292.69(226.99-370.59) | 105810.87(81281.08-136144.29) | 302.99(233.52-387.34) | 0.16(0.13,0.19) | 133.62 | 3.52 |
| Maldives | 451.25(342.28-586.16) | 292.36(228.23-366.88) | 2062.66(1565.45-2632.19) | 308.46(237.95-390.33) | 0.23(0.19,0.27) | 357.1 | 5.5 |
| Mali | 13561.89(10402.89-17366.18) | 222.54(173.06-280.92) | 36424.77(27601.81-47064.49) | 226.36(177.18-285.12) | 0.05(0.05,0.05) | 168.58 | 1.72 |
| Malta | 1091.03(860.33-1372.44) | 264.42(208.50-334.14) | 1443.39(1148.33-1790.43) | 267.72(210.46-335.98) | 0.02(0.01,0.03) | 32.3 | 1.25 |
| Marshall Islands | 83.28(62.58-107.37) | 267.47(206.66-339.63) | 148.33(112.92-189.77) | 266.62(204.97-337.11) | -0.01(-0.02,-0.00) | 78.11 | -0.32 |
| Mauritania | 3469.91(2637.10-4452.81) | 233.24(182.26-294.45) | 7910.26(6150.37-10167.49) | 235.69(185.12-297.99) | 0.03(0.03,0.04) | 127.97 | 1.05 |
| Mauritius | 3163.24(2397.75-4157.90) | 289.29(223.65-370.27) | 4481.72(3510.54-5532.14) | 297.12(231.36-373.43) | 0.13(0.11,0.16) | 41.68 | 2.71 |
| Mexico | 167905.34(136618.47-202959.88) | 247.28(204.86-294.29) | 343771.23(283935.35-410244.79) | 248.56(205.58-296.67) | 0.01(0.01,0.02) | 104.74 | 0.52 |
| Micronesia (Federated States of) | 202.87(152.08-263.79) | 266.01(205.21-337.33) | 266.16(202.30-341.35) | 265.56(202.96-337.94) | -0.00(-0.01,0.00) | 31.2 | -0.17 |
| Monaco | 110.09(88.52-138.12) | 283.96(224.63-362.70) | 133.52(106.73-166.69) | 284.99(223.29-364.26) | -0.01(-0.02,0.01) | 21.29 | 0.36 |
| Mongolia | 3380.72(2532.90-4386.52) | 206.21(160.16-260.36) | 7262.97(5513.99-9195.79) | 213.46(163.90-269.39) | 0.13(0.12,0.15) | 114.83 | 3.52 |
| Montenegro | 1444.73(1124.00-1840.60) | 219.87(171.08-279.75) | 1597.59(1264.48-2000.18) | 221.14(171.82-280.44) | 0.04(0.03,0.05) | 10.58 | 0.58 |
| Morocco | 50138.11(38056.36-64726.51) | 239.06(186.32-303.98) | 94369.51(73274.35-118604.34) | 239.86(186.41-301.40) | -0.00(-0.01,0.01) | 88.22 | 0.33 |
| Mozambique | 19422.00(14856.37-24714.68) | 201.55(160.16-254.94) | 45584.59(34468.91-59183.23) | 208.21(162.49-265.08) | 0.14(0.13,0.16) | 134.71 | 3.31 |
| Myanmar | 89132.17(68290.50-114348.13) | 253.95(197.56-319.80) | 155573.65(120754.38-195858.98) | 269.19(209.41-337.81) | 0.28(0.24,0.32) | 74.54 | 6 |
| Namibia | 2299.95(1746.82-2953.96) | 221.89(173.38-280.49) | 5047.83(3809.87-6471.15) | 226.24(174.11-287.11) | 0.09(0.08,0.10) | 119.48 | 1.96 |
| Nauru | 24.51(18.51-31.61) | 295.70(228.74-375.64) | 27.58(20.72-35.33) | 281.45(215.73-353.99) | -0.15(-0.24,-0.06) | 12.56 | -4.82 |
| Nepal | 42431.21(32342.83-53638.08) | 284.05(221.39-354.39) | 82907.71(63526.55-104491.11) | 277.57(215.13-345.00) | -0.10(-0.11,-0.09) | 95.39 | -2.28 |
| Netherlands | 65767.11(56912.72-75527.16) | 380.36(329.43-437.87) | 74733.69(62338.57-88904.05) | 364.86(302.96-441.61) | -0.17(-0.21,-0.12) | 13.63 | -4.07 |
| New Zealand | 13778.86(11383.70-16306.27) | 378.71(312.87-448.38) | 22083.28(18694.75-25936.36) | 381.86(319.64-450.34) | 0.02(0.01,0.03) | 60.27 | 0.83 |
| Nicaragua | 6257.84(4689.12-8092.41) | 235.72(185.89-297.43) | 15540.38(11960.48-19975.44) | 235.76(184.17-299.06) | 0.01(-0.00,0.02) | 148.33 | 0.02 |
| Niger | 11751.21(8923.61-15256.75) | 222.51(175.76-280.49) | 34150.83(25798.76-43742.20) | 221.80(171.24-278.39) | -0.01(-0.02,-0.00) | 190.62 | -0.32 |
| Nigeria | 162126.12(133406.39-194519.39) | 240.52(200.53-286.28) | 422166.44(345317.99-510658.44) | 248.73(207.33-296.93) | 0.15(0.13,0.17) | 160.39 | 3.41 |
| Niue | 5.75(4.45-7.21) | 286.67(221.40-359.63) | 5.17(4.04-6.45) | 290.41(223.15-368.02) | 0.08(0.06,0.09) | -10.09 | 1.3 |
| North Macedonia | 4574.20(3536.03-5845.91) | 218.99(169.56-279.30) | 6037.10(4762.80-7629.60) | 221.22(172.80-281.33) | 0.03(0.03,0.04) | 31.98 | 1.02 |
| Northern Mariana Islands | 157.02(117.01-204.82) | 322.26(244.76-409.92) | 167.72(130.74-208.92) | 308.90(236.55-389.76) | -0.16(-0.18,-0.13) | 6.81 | -4.15 |
| Norway | 13059.16(10968.73-15326.80) | 273.88(228.67-323.18) | 17557.86(14914.81-20467.28) | 274.25(230.81-325.38) | 0.01(0.01,0.02) | 34.45 | 0.14 |
| Oman | 4329.59(3222.92-5623.74) | 256.75(197.56-326.75) | 14659.10(11097.94-19177.26) | 258.00(199.45-329.40) | 0.02(0.01,0.02) | 238.58 | 0.49 |
| Pakistan | 238893.95(196996.77-284422.37) | 295.01(243.39-349.96) | 602480.38(495121.75-724316.72) | 290.38(240.87-344.82) | -0.08(-0.08,-0.07) | 152.2 | -1.57 |
| Palau | 42.68(32.42-54.28) | 288.81(223.49-364.56) | 64.93(51.10-81.83) | 293.72(224.83-370.52) | 0.04(0.03,0.06) | 52.12 | 1.7 |
| Palestine | 3355.32(2502.95-4408.07) | 243.27(187.56-311.55) | 10983.96(8281.59-14285.56) | 243.22(188.56-310.07) | -0.03(-0.05,-0.02) | 227.36 | -0.02 |
| Panama | 5062.77(3933.07-6504.07) | 243.08(190.94-307.09) | 10939.14(8468.73-13859.22) | 249.15(192.77-316.16) | 0.08(0.07,0.10) | 116.07 | 2.5 |
| Papua New Guinea | 8876.97(6707.07-11495.61) | 271.49(210.59-342.23) | 25422.42(19348.75-32869.48) | 273.43(211.32-346.80) | 0.01(-0.01,0.02) | 186.39 | 0.71 |
| Paraguay | 7643.41(5854.72-9756.54) | 240.20(187.50-299.61) | 17570.66(13620.71-22249.18) | 242.72(189.94-305.16) | 0.02(0.01,0.03) | 129.88 | 1.05 |
| Peru | 41991.12(32203.06-54260.80) | 237.94(185.35-302.17) | 92033.00(71389.97-116003.61) | 242.50(188.69-304.72) | 0.07(0.05,0.08) | 119.17 | 1.92 |
| Philippines | 144545.82(117486.83-172695.24) | 280.65(233.27-333.64) | 319558.44(263009.38-380175.90) | 284.73(236.89-337.52) | 0.09(0.06,0.12) | 121.08 | 1.45 |
| Poland | 90984.47(75569.98-108350.80) | 220.31(182.90-262.26) | 105993.68(89000.08-124491.20) | 226.82(188.66-270.46) | 0.09(0.09,0.10) | 16.5 | 2.95 |
| Portugal | 28889.54(22985.29-35627.98) | 258.38(203.94-323.10) | 34177.54(27377.89-42024.30) | 260.31(205.42-327.74) | 0.00(-0.01,0.01) | 18.3 | 0.74 |
| Puerto Rico | 8468.44(6625.23-10723.19) | 234.40(183.66-296.18) | 9198.78(7352.31-11487.00) | 236.73(185.65-304.10) | 0.04(0.03,0.05) | 8.62 | 0.99 |
| Qatar | 1413.73(1048.65-1859.99) | 273.24(213.72-349.05) | 11813.96(8815.00-15481.63) | 276.27(212.05-354.74) | 0.09(0.07,0.10) | 735.66 | 1.11 |
| Republic of Korea | 118524.45(90445.92-153355.91) | 257.35(201.39-326.37) | 173643.89(137977.61-214984.39) | 258.87(203.30-324.92) | 0.01(0.01,0.02) | 46.5 | 0.59 |
| Republic of Moldova | 9166.84(7068.83-11623.07) | 197.86(152.80-250.50) | 8987.35(7077.24-11285.40) | 198.51(154.40-249.50) | 0.01(-0.01,0.03) | -1.96 | 0.33 |
| Romania | 54245.04(42085.01-68705.36) | 217.04(167.13-276.78) | 49850.51(39344.34-62264.22) | 221.61(174.31-277.76) | 0.09(0.07,0.10) | -8.1 | 2.11 |
| Russian Federation | 322859.86(269208.06-383076.75) | 191.44(158.97-228.10) | 358773.70(301905.32-422537.31) | 207.01(171.73-246.76) | 0.38(0.30,0.46) | 11.12 | 8.14 |
| Rwanda | 10751.45(8145.32-14096.41) | 214.38(166.35-271.57) | 24482.95(18624.68-31581.90) | 216.56(169.89-274.17) | 0.08(0.06,0.10) | 127.72 | 1.02 |
| Saint Kitts and Nevis | 79.99(61.77-102.60) | 226.77(177.46-286.46) | 168.64(131.91-211.97) | 233.36(181.48-295.52) | 0.08(0.07,0.09) | 110.83 | 2.9 |
| Saint Lucia | 246.28(187.94-318.94) | 224.46(176.07-286.83) | 487.99(379.12-612.25) | 227.24(176.51-288.47) | 0.02(0.01,0.03) | 98.15 | 1.24 |
| Saint Vincent and the Grenadines | 193.13(146.89-251.24) | 220.85(172.94-280.24) | 284.06(224.28-355.45) | 223.99(176.47-281.73) | 0.05(0.04,0.06) | 47.08 | 1.42 |
| Samoa | 350.18(264.61-450.48) | 278.92(214.13-351.59) | 514.71(394.87-651.83) | 281.06(217.03-352.77) | 0.04(0.03,0.04) | 46.98 | 0.77 |
| San Marino | 74.51(59.14-93.71) | 272.57(214.44-344.89) | 108.48(86.42-134.19) | 269.73(210.46-339.49) | -0.04(-0.04,-0.03) | 45.59 | -1.04 |
| Sao Tome and Principe | 190.77(147.89-244.22) | 235.28(185.97-294.64) | 449.81(340.98-572.97) | 236.01(182.76-295.68) | 0.01(0.01,0.02) | 135.79 | 0.31 |
| Saudi Arabia | 32839.42(24668.90-42548.97) | 256.78(198.37-323.56) | 123530.58(94130.13-159015.42) | 256.72(199.11-326.64) | -0.00(-0.01,0.01) | 276.17 | -0.02 |
| Senegal | 11968.84(9075.53-15393.27) | 231.63(180.81-291.27) | 29281.44(22338.15-37737.28) | 232.56(182.72-294.10) | 0.02(0.01,0.02) | 144.65 | 0.4 |
| Serbia | 23604.97(18239.13-29770.38) | 219.61(169.04-278.35) | 23503.84(18699.01-29096.48) | 220.34(170.91-277.95) | 0.03(0.02,0.05) | -0.43 | 0.33 |
| Seychelles | 194.00(147.50-249.56) | 293.73(226.97-372.49) | 364.18(284.09-462.82) | 301.89(233.92-386.11) | 0.11(0.09,0.14) | 87.73 | 2.78 |
| Sierra Leone | 7073.72(5469.73-9188.41) | 224.79(176.34-283.97) | 15685.19(11666.84-20282.59) | 222.34(170.16-278.07) | -0.04(-0.05,-0.03) | 121.74 | -1.09 |
| Singapore | 8736.68(6749.69-11259.84) | 252.41(196.59-320.42) | 19472.33(15246.41-24379.10) | 255.17(200.39-322.59) | 0.02(0.01,0.02) | 122.88 | 1.09 |
| Slovakia | 12409.32(9643.58-15762.33) | 220.93(171.40-281.15) | 15099.14(11930.92-19272.88) | 225.78(176.81-290.63) | 0.08(0.07,0.08) | 21.68 | 2.19 |
| Slovenia | 5021.51(3883.83-6388.94) | 224.42(173.34-287.08) | 5692.76(4512.24-7172.18) | 228.75(177.97-292.74) | 0.05(0.04,0.06) | 13.37 | 1.93 |
| Solomon Islands | 623.50(469.26-799.97) | 261.40(203.01-330.50) | 1532.99(1166.67-1976.43) | 259.51(201.09-328.08) | -0.04(-0.06,-0.02) | 145.87 | -0.72 |
| Somalia | 11077.26(8416.09-14461.24) | 200.59(157.30-256.10) | 29072.02(21898.24-37418.65) | 196.06(152.11-248.66) | -0.07(-0.08,-0.06) | 162.45 | -2.26 |
| South Africa | 71308.01(58309.17-86000.65) | 227.34(187.98-270.92) | 138890.88(113941.79-166760.24) | 228.35(190.17-273.31) | 0.04(0.03,0.05) | 94.78 | 0.45 |
| South Sudan | 9323.84(7058.73-12093.71) | 224.18(177.02-284.50) | 15337.73(11606.02-19631.27) | 218.19(170.13-276.58) | -0.06(-0.08,-0.03) | 64.5 | -2.67 |
| Spain | 112695.31(106583.33-118017.29) | 264.13(248.77-276.72) | 151433.37(143138.29-159201.28) | 265.77(249.90-278.89) | 0.02(0.01,0.02) | 34.37 | 0.62 |
| Sri Lanka | 45929.87(35336.07-58552.92) | 281.00(217.13-354.73) | 70048.65(54736.43-87781.86) | 290.23(225.30-366.69) | 0.16(0.12,0.19) | 52.51 | 3.29 |
| Sudan | 33863.43(25593.43-43726.46) | 229.56(175.49-290.80) | 85031.40(64304.34-110411.92) | 231.44(179.66-292.51) | 0.02(0.01,0.03) | 151.1 | 0.82 |
| Suriname | 674.96(564.89-786.07) | 197.46(167.18-231.54) | 1236.93(1051.72-1454.33) | 198.51(169.00-233.35) | 0.03(0.02,0.04) | 83.26 | 0.53 |
| Sweden | 28130.83(24440.95-32092.49) | 278.14(240.60-320.21) | 33889.46(29160.03-39444.93) | 273.42(231.48-321.08) | -0.06(-0.07,-0.05) | 20.47 | -1.7 |
| Switzerland | 22389.09(17616.93-27981.03) | 272.73(213.43-340.80) | 29837.64(23778.82-37073.50) | 271.04(212.87-339.94) | -0.01(-0.02,0.00) | 33.27 | -0.62 |
| Syrian Arab Republic | 21263.53(15960.91-27553.24) | 241.95(186.90-306.30) | 33171.01(26211.29-41737.05) | 238.50(185.97-302.70) | -0.05(-0.06,-0.03) | 56 | -1.43 |
| Taiwan (Province of China) | 61577.73(47699.24-77394.14) | 291.77(230.54-362.27) | 93166.82(73649.62-115077.03) | 310.30(239.58-386.89) | 0.14(0.11,0.17) | 51.3 | 6.35 |
| Tajikistan | 8416.57(6373.90-10824.68) | 211.80(165.17-268.57) | 19674.15(14866.54-25060.39) | 208.44(162.07-262.80) | -0.04(-0.07,-0.01) | 133.76 | -1.59 |
| Thailand | 156768.96(118977.80-201591.05) | 280.28(216.20-353.83) | 234879.01(186113.37-292734.36) | 288.98(223.94-366.57) | 0.15(0.12,0.17) | 49.82 | 3.1 |
| Timor-Leste | 1567.93(1191.77-2015.74) | 248.88(194.97-311.39) | 2903.98(2197.94-3717.50) | 253.26(195.33-318.96) | 0.16(0.12,0.19) | 85.21 | 1.76 |
| Togo | 5520.21(4123.24-7098.66) | 224.95(176.74-281.05) | 15646.19(11930.04-19965.39) | 223.86(173.28-280.14) | -0.02(-0.02,-0.01) | 183.43 | -0.48 |
| Tokelau | 3.62(2.78-4.55) | 275.14(212.88-344.95) | 3.80(2.92-4.84) | 281.99(216.14-361.56) | 0.09(0.08,0.11) | 4.98 | 2.49 |
| Tonga | 207.27(159.95-265.66) | 282.09(219.95-359.11) | 258.01(196.32-323.81) | 283.93(218.35-355.40) | 0.02(0.01,0.03) | 24.48 | 0.65 |
| Trinidad and Tobago | 1990.73(1772.54-2241.79) | 182.50(162.02-205.53) | 3812.27(3053.09-4696.42) | 233.32(183.83-286.77) | 0.61(0.39,0.83) | 91.5 | 27.85 |
| Tunisia | 17387.55(13227.47-22480.27) | 246.77(191.15-312.57) | 32892.65(25577.67-41930.30) | 247.31(191.29-315.98) | -0.00(-0.01,0.00) | 89.17 | 0.22 |
| Turkey | 123012.82(101106.49-148654.31) | 243.88(201.93-290.92) | 231007.95(193584.89-273816.91) | 246.19(205.15-293.80) | 0.02(0.01,0.03) | 87.79 | 0.95 |
| Turkmenistan | 6233.68(4680.21-8135.04) | 213.59(166.81-273.34) | 11473.35(8785.60-14531.65) | 219.69(169.44-275.59) | 0.11(0.08,0.14) | 84.05 | 2.86 |
| Tuvalu | 22.41(17.06-28.51) | 262.87(201.79-332.63) | 32.33(24.97-40.60) | 270.35(209.58-338.88) | 0.08(0.07,0.09) | 44.27 | 2.84 |
| Uganda | 23448.83(17978.39-30249.52) | 207.40(162.97-262.42) | 64061.26(48497.59-83117.16) | 212.94(167.85-269.55) | 0.10(0.09,0.11) | 173.2 | 2.67 |
| Ukraine | 120056.75(99866.55-143709.82) | 204.06(168.74-244.24) | 107908.00(90380.82-126858.46) | 202.22(168.14-240.23) | -0.01(-0.03,0.00) | -10.12 | -0.9 |
| United Arab Emirates | 5651.40(4100.01-7439.62) | 275.86(212.19-353.30) | 39412.73(30201.78-50621.36) | 265.87(207.02-337.08) | -0.12(-0.13,-0.11) | 597.4 | -3.62 |
| United Kingdom | 158754.92(136151.18-182989.80) | 244.89(207.32-284.79) | 171821.47(146893.88-199169.16) | 214.28(180.74-250.43) | -0.33(-0.47,-0.19) | 8.23 | -12.5 |
| United Republic of Tanzania | 37996.35(28713.04-49325.63) | 214.52(169.07-271.80) | 98829.85(76249.34-125642.03) | 217.99(169.67-273.18) | 0.08(0.07,0.10) | 160.1 | 1.62 |
| United States Virgin Islands | 248.85(193.13-317.14) | 233.73(181.84-296.64) | 242.80(192.55-301.07) | 239.19(186.95-302.96) | 0.07(0.05,0.09) | -2.43 | 2.34 |
| United States of America | 1042439.46(869137.97-1231896.72) | 370.36(308.78-437.53) | 1332388.70(1126996.89-1559304.45) | 354.43(298.14-415.84) | -0.10(-0.15,-0.04) | 27.81 | -4.3 |
| Uruguay | 8854.77(6991.31-11044.41) | 277.75(217.83-349.27) | 10542.83(8361.72-13154.51) | 278.69(220.41-351.97) | -0.00(-0.02,0.01) | 19.06 | 0.34 |
| Uzbekistan | 34447.27(26524.14-44913.86) | 209.64(163.45-265.56) | 76049.99(58210.69-96817.98) | 212.70(164.22-269.00) | 0.06(0.04,0.07) | 120.77 | 1.46 |
| Vanuatu | 305.36(225.92-394.50) | 267.13(203.71-337.12) | 726.29(546.06-938.07) | 265.15(204.94-336.15) | -0.01(-0.03,-0.00) | 137.85 | -0.74 |
| Venezuela (Bolivarian Republic of) | 38326.80(29273.63-49349.25) | 243.05(191.06-306.70) | 69415.62(54852.06-86712.07) | 239.03(188.52-298.19) | -0.01(-0.03,0.00) | 81.12 | -1.65 |
| Viet Nam | 174128.11(132610.27-218984.76) | 306.26(234.24-379.05) | 356944.71(278085.91-441848.48) | 319.48(249.90-395.68) | 0.19(0.17,0.21) | 104.99 | 4.31 |
| Yemen | 20235.46(15371.73-26257.12) | 230.50(178.10-292.64) | 61792.31(47221.12-80436.59) | 225.33(176.32-287.01) | -0.06(-0.07,-0.04) | 205.37 | -2.24 |
| Zambia | 11221.61(8483.54-14444.39) | 213.41(168.18-266.02) | 32640.45(24654.79-42417.93) | 217.31(171.40-274.77) | 0.10(0.08,0.12) | 190.87 | 1.83 |
| Zimbabwe | 14945.91(11104.96-19656.81) | 212.89(164.77-272.73) | 26179.04(19783.25-33483.89) | 207.61(161.86-263.73) | -0.12(-0.15,-0.09) | 75.16 | -2.48 |

Abbreviations: ASR, age-standardized rate; RC, relative change; EAPC, estimated annual percentage change; SDI, sociodemographic index; UI, uncertainty interval; CI, confidence interval.

**Supplementary Tables S6** The number and ASR of DALYs of Schizophrenia burden for the 204 countries/regions.

| Location | 1990 Case number  (95% UI) | 1990 ASR  (95% UI) | 2021 Case number  (95% UI) | 2021 ASR  (95% UI) | EAPCs  (95%, CI) | RC of numbers (%) | RC of ASR (%) |
| --- | --- | --- | --- | --- | --- | --- | --- |
| Afghanistan | 9623.02(6841.78-12972.95) | 137.81(97.87-185.29) | 31056.98(21690.94-43493.39) | 135.78(96.75-187.94) | -0.00(-0.05,0.04) | 222.74 | -1.47 |
| Albania | 4192.29(2915.41-5818.61) | 138.75(97.13-189.22) | 4359.44(3023.97-5910.84) | 142.10(98.39-193.31) | 0.10(0.09,0.11) | 3.99 | 2.41 |
| Algeria | 30469.67(21305.95-42409.99) | 158.86(110.76-215.43) | 72512.58(51597.70-100702.95) | 157.60(112.69-217.68) | -0.02(-0.02,-0.01) | 137.98 | -0.79 |
| American Samoa | 78.71(54.76-110.87) | 194.20(136.35-267.42) | 92.43(64.77-125.50) | 186.35(131.43-252.77) | -0.11(-0.12,-0.09) | 17.43 | -4.04 |
| Andorra | 114.81(80.61-155.93) | 175.71(124.07-237.86) | 197.05(141.37-264.47) | 173.11(123.47-235.20) | -0.04(-0.05,-0.03) | 71.64 | -1.48 |
| Angola | 10444.63(7212.17-14458.32) | 138.40(95.79-188.49) | 33230.93(23005.40-46610.06) | 141.15(99.23-193.86) | 0.13(0.10,0.16) | 218.16 | 1.99 |
| Antigua and Barbuda | 84.81(58.64-118.87) | 149.66(104.26-205.61) | 157.36(109.15-215.40) | 149.48(103.76-204.67) | 0.01(-0.00,0.02) | 85.55 | -0.12 |
| Argentina | 56543.44(39203.10-76068.78) | 177.48(123.12-238.58) | 87784.97(60578.62-118096.44) | 176.76(121.75-238.35) | -0.01(-0.02,0.00) | 55.25 | -0.41 |
| Armenia | 4749.13(3373.73-6451.44) | 140.02(99.04-187.93) | 4935.74(3435.42-6643.96) | 141.98(98.27-193.16) | 0.08(0.07,0.09) | 3.93 | 1.4 |
| Australia | 45472.23(33577.29-55294.15) | 247.44(182.60-301.43) | 71431.67(53870.93-86605.39) | 247.58(186.20-300.20) | 0.01(0.00,0.02) | 57.09 | 0.06 |
| Austria | 15276.94(10766.89-20944.34) | 170.05(119.82-235.04) | 18647.65(13241.42-24826.44) | 169.98(120.07-229.08) | 0.00(-0.00,0.01) | 22.06 | -0.04 |
| Azerbaijan | 9412.43(6490.84-13004.36) | 139.88(94.17-192.47) | 17448.66(12195.78-24182.81) | 142.23(100.17-196.67) | 0.11(0.07,0.15) | 85.38 | 1.68 |
| Bahamas | 360.05(248.13-504.21) | 149.67(104.52-205.66) | 649.42(461.72-892.67) | 147.19(104.83-202.11) | -0.04(-0.05,-0.03) | 80.37 | -1.66 |
| Bahrain | 894.44(610.47-1241.88) | 167.98(117.65-231.73) | 3270.15(2245.21-4584.47) | 167.80(116.55-233.31) | 0.01(0.01,0.02) | 265.61 | -0.1 |
| Bangladesh | 147375.65(102280.91-201489.72) | 184.91(129.66-248.65) | 303903.11(213672.18-419010.10) | 181.55(127.73-250.72) | -0.07(-0.08,-0.06) | 106.21 | -1.82 |
| Barbados | 436.44(312.99-579.64) | 168.63(120.99-222.83) | 561.74(389.56-752.48) | 158.62(110.54-215.68) | -0.07(-0.10,-0.03) | 28.71 | -5.94 |
| Belarus | 14745.74(10502.13-20102.64) | 128.90(91.11-177.24) | 14656.80(10338.12-19441.64) | 130.94(91.44-175.78) | 0.08(0.06,0.10) | -0.6 | 1.59 |
| Belgium | 19602.27(13910.10-26315.07) | 169.61(120.45-228.95) | 22525.17(15260.35-29633.47) | 167.87(116.47-226.54) | -0.01(-0.03,-0.00) | 14.91 | -1.03 |
| Belize | 192.02(131.90-266.86) | 142.54(99.34-198.90) | 600.26(418.49-827.45) | 141.74(98.97-195.99) | -0.03(-0.04,-0.02) | 212.6 | -0.56 |
| Benin | 4673.65(3357.20-6445.12) | 144.30(102.83-195.01) | 14463.01(10164.56-19925.58) | 146.24(101.63-200.49) | 0.04(0.03,0.05) | 209.46 | 1.35 |
| Bermuda | 109.39(74.55-148.03) | 156.65(107.15-210.95) | 123.51(85.43-166.88) | 158.38(111.49-215.68) | 0.05(0.04,0.06) | 12.9 | 1.11 |
| Bhutan | 888.33(611.70-1230.66) | 187.43(129.70-250.93) | 1556.40(1082.37-2126.42) | 190.33(134.90-257.92) | 0.04(0.03,0.05) | 75.2 | 1.54 |
| Bolivia (Plurinational State of) | 7326.19(5092.51-10156.57) | 147.06(105.25-200.53) | 17514.53(12434.20-24115.88) | 149.05(104.41-204.46) | 0.05(0.04,0.06) | 139.07 | 1.35 |
| Bosnia and Herzegovina | 6658.90(4708.16-9056.39) | 135.94(96.51-184.73) | 5574.52(3928.28-7473.87) | 141.99(100.21-194.76) | 0.16(0.14,0.18) | -16.28 | 4.45 |
| Botswana | 1321.92(936.90-1825.73) | 139.26(98.52-191.06) | 3548.79(2439.45-4957.70) | 142.56(98.75-197.82) | 0.11(0.09,0.13) | 168.46 | 2.37 |
| Brazil | 202377.32(150091.77-260202.93) | 152.12(112.46-195.52) | 382072.21(278550.46-489510.54) | 153.38(112.55-197.29) | 0.05(0.04,0.07) | 88.79 | 0.83 |
| Brunei Darussalam | 409.39(287.36-571.64) | 171.17(122.55-236.20) | 905.16(636.00-1251.58) | 167.77(118.50-230.57) | -0.04(-0.05,-0.03) | 121.1 | -1.99 |
| Bulgaria | 13566.69(9396.72-18361.70) | 137.93(96.20-189.86) | 11371.67(7784.35-15231.00) | 139.66(96.59-191.71) | 0.06(0.05,0.07) | -16.18 | 1.26 |
| Burkina Faso | 8922.93(6269.87-12290.75) | 140.49(98.09-191.81) | 23662.11(16617.70-32387.48) | 143.29(100.35-195.07) | 0.07(0.06,0.07) | 165.18 | 2 |
| Burundi | 5322.42(3767.34-7437.71) | 134.81(94.86-185.56) | 13214.39(9140.57-18263.30) | 131.53(93.20-180.67) | -0.07(-0.08,-0.06) | 148.28 | -2.44 |
| Cabo Verde | 371.28(265.73-511.89) | 153.36(106.38-206.42) | 924.54(653.96-1246.76) | 153.91(109.85-206.31) | 0.03(0.02,0.04) | 149.01 | 0.36 |
| Cambodia | 12557.59(8945.61-17351.49) | 163.61(116.72-221.70) | 29743.52(21048.39-40481.85) | 172.53(123.00-235.10) | 0.24(0.20,0.28) | 136.86 | 5.45 |
| Cameroon | 10678.19(7418.92-14781.05) | 144.18(101.09-198.62) | 35776.33(24451.90-49152.36) | 144.04(98.78-194.38) | 0.01(-0.00,0.02) | 235.04 | -0.09 |
| Canada | 57358.81(43499.22-68428.80) | 185.07(140.21-220.79) | 79715.44(60208.10-95298.49) | 184.22(139.65-220.83) | -0.01(-0.01,-0.01) | 38.98 | -0.46 |
| Central African Republic | 2581.85(1824.61-3610.83) | 126.24(87.41-172.63) | 5460.54(3751.17-7589.99) | 124.80(85.12-171.28) | -0.02(-0.03,-0.01) | 111.5 | -1.15 |
| Chad | 5737.36(4075.02-7957.80) | 141.09(99.27-191.76) | 16091.80(11172.08-22295.37) | 142.48(100.96-196.95) | 0.06(0.05,0.07) | 180.47 | 0.99 |
| Chile | 22761.34(16118.75-31181.02) | 176.79(124.81-240.53) | 38642.13(27416.31-52523.53) | 178.89(126.12-242.12) | 0.04(0.03,0.05) | 69.77 | 1.19 |
| China | 2329187.49(1763155.68-2915190.38) | 195.67(147.78-244.07) | 3445845.29(2572494.69-4306768.38) | 203.88(152.53-255.67) | 0.08(0.06,0.11) | 47.94 | 4.2 |
| Colombia | 43502.60(29326.03-60237.75) | 153.42(106.41-210.61) | 83985.66(59214.72-116484.58) | 155.00(108.87-215.38) | 0.04(0.03,0.05) | 93.06 | 1.03 |
| Comoros | 469.05(322.86-653.94) | 144.20(99.91-199.43) | 1018.49(710.28-1399.60) | 143.62(101.10-197.20) | 0.02(0.01,0.03) | 117.14 | -0.4 |
| Congo | 2420.98(1741.43-3340.81) | 137.99(97.23-186.12) | 6813.98(4757.73-9270.26) | 138.55(97.70-187.63) | 0.06(0.03,0.08) | 181.46 | 0.41 |
| Cook Islands | 32.68(22.76-45.56) | 194.67(136.03-268.03) | 37.14(26.60-50.06) | 195.57(137.22-267.66) | 0.02(0.01,0.04) | 13.67 | 0.47 |
| Costa Rica | 4127.38(2881.36-5722.50) | 157.27(108.29-214.37) | 8413.47(5862.47-11392.45) | 157.53(110.09-213.39) | 0.02(0.01,0.03) | 103.85 | 0.17 |
| Croatia | 8029.17(5746.58-10825.33) | 143.10(101.93-195.37) | 7176.96(5024.16-9611.28) | 144.85(101.51-198.84) | 0.06(0.05,0.06) | -10.61 | 1.23 |
| Cuba | 16799.89(11747.50-23393.72) | 148.62(103.47-204.14) | 19796.65(14079.17-26787.04) | 146.75(104.23-199.36) | 0.00(-0.02,0.03) | 17.84 | -1.26 |
| Cyprus | 1395.44(978.21-1882.88) | 170.07(119.06-229.49) | 2896.03(2018.63-3873.00) | 170.57(119.34-230.09) | -0.01(-0.01,-0.00) | 107.54 | 0.29 |
| Czechia | 16408.21(11511.56-22335.87) | 144.05(100.66-198.42) | 18363.48(12832.19-24689.99) | 146.44(102.75-201.14) | 0.04(0.03,0.04) | 11.92 | 1.66 |
| C么te d'Ivoire | 12428.32(8766.11-17589.49) | 141.40(99.71-195.91) | 32776.08(22466.06-45400.99) | 144.24(101.47-197.91) | 0.04(0.03,0.06) | 163.72 | 2 |
| Democratic People's Republic of Korea | 37194.95(25685.23-50953.49) | 178.53(122.88-243.20) | 53902.97(38030.32-74512.37) | 171.76(121.75-239.10) | -0.15(-0.17,-0.14) | 44.92 | -3.79 |
| Democratic Republic of the Congo | 36313.32(24880.15-50183.40) | 133.52(91.82-180.53) | 92650.95(63823.95-127660.39) | 131.37(92.48-177.14) | -0.03(-0.07,0.01) | 155.14 | -1.61 |
| Denmark | 9470.21(6854.38-12495.15) | 157.37(114.23-207.52) | 15447.29(11675.22-18873.54) | 231.39(172.97-282.75) | 1.77(1.44,2.11) | 63.11 | 47.03 |
| Djibouti | 457.73(315.72-629.65) | 144.06(99.93-196.07) | 1785.15(1206.90-2459.95) | 143.34(97.44-196.11) | 0.00(-0.01,0.02) | 290 | -0.5 |
| Dominica | 89.55(62.12-123.23) | 144.05(98.84-196.30) | 107.96(73.80-145.32) | 143.90(98.72-195.74) | -0.00(-0.01,0.01) | 20.56 | -0.1 |
| Dominican Republic | 8368.24(5856.84-11588.07) | 142.18(101.07-193.16) | 16573.33(11445.83-22729.19) | 145.54(100.67-198.11) | 0.09(0.08,0.10) | 98.05 | 2.37 |
| Ecuador | 12438.99(8648.38-17265.74) | 152.42(106.37-211.96) | 27765.67(19613.59-38230.60) | 152.34(107.39-208.75) | 0.03(0.01,0.04) | 123.21 | -0.06 |
| Egypt | 70778.61(49466.29-97894.97) | 153.97(107.97-209.73) | 153468.55(109672.19-211068.52) | 155.18(110.19-211.67) | 0.04(0.03,0.05) | 116.83 | 0.78 |
| El Salvador | 5946.60(4167.99-8231.33) | 145.03(101.59-198.21) | 9363.64(6745.84-12634.67) | 147.05(104.52-198.07) | 0.06(0.05,0.07) | 57.46 | 1.39 |
| Equatorial Guinea | 401.34(286.69-550.83) | 133.13(95.15-181.14) | 1816.01(1268.91-2534.10) | 147.52(103.84-199.16) | 0.48(0.39,0.57) | 352.49 | 10.81 |
| Eritrea | 3244.63(2270.76-4464.48) | 133.91(94.03-183.73) | 7782.57(5270.91-10682.06) | 136.19(94.24-186.85) | 0.07(0.05,0.08) | 139.86 | 1.71 |
| Estonia | 2253.83(1573.51-3077.96) | 130.13(90.36-176.62) | 2041.89(1409.92-2778.39) | 134.58(93.55-185.01) | 0.13(0.11,0.14) | -9.4 | 3.42 |
| Eswatini | 757.14(530.44-1053.70) | 139.00(96.44-190.90) | 1414.68(975.03-1941.64) | 136.74(95.63-186.22) | -0.04(-0.06,-0.02) | 86.84 | -1.63 |
| Ethiopia | 48686.67(35973.49-63289.76) | 138.42(103.23-176.34) | 121956.11(89921.52-157646.20) | 141.78(104.33-182.66) | 0.12(0.10,0.14) | 150.49 | 2.43 |
| Fiji | 1227.11(860.16-1701.60) | 182.34(127.68-246.88) | 1729.03(1198.82-2371.03) | 183.94(127.67-251.90) | 0.05(0.04,0.06) | 40.9 | 0.88 |
| Finland | 10430.56(7582.28-13571.86) | 176.59(128.34-230.84) | 10935.62(7663.36-14617.00) | 167.62(115.54-226.57) | -0.17(-0.22,-0.13) | 4.84 | -5.08 |
| France | 106480.30(73896.56-141321.19) | 165.58(114.48-221.15) | 124342.00(86698.47-164638.90) | 165.09(115.78-224.03) | -0.02(-0.02,-0.01) | 16.77 | -0.29 |
| Gabon | 1110.64(786.68-1534.86) | 146.20(103.60-199.48) | 2370.41(1643.63-3281.43) | 145.60(100.82-200.12) | 0.02(0.01,0.02) | 113.43 | -0.41 |
| Gambia | 1003.13(699.87-1406.87) | 144.48(102.15-197.79) | 2665.94(1843.14-3676.87) | 143.05(99.64-196.42) | -0.02(-0.03,-0.01) | 165.76 | -0.99 |
| Georgia | 8256.87(5781.77-11403.90) | 141.01(98.70-194.70) | 5621.86(3906.46-7699.71) | 139.40(97.95-192.95) | 0.02(-0.01,0.05) | -31.91 | -1.14 |
| Germany | 148842.55(105715.57-199876.68) | 154.84(109.68-208.60) | 159030.99(113354.41-210121.87) | 155.11(111.22-207.98) | -0.02(-0.03,0.00) | 6.85 | 0.17 |
| Ghana | 16115.62(11223.11-22255.00) | 144.89(101.97-198.53) | 44261.02(30265.78-61196.94) | 148.63(102.67-201.84) | 0.11(0.10,0.12) | 174.65 | 2.59 |
| Greece | 19842.81(13868.37-27012.65) | 168.59(118.12-230.36) | 20599.30(14485.51-27144.43) | 167.20(117.55-227.51) | -0.02(-0.03,-0.01) | 3.81 | -0.82 |
| Greenland | 130.64(91.77-179.56) | 210.46(148.77-284.50) | 135.59(96.88-181.93) | 209.62(149.26-281.77) | 0.09(0.04,0.13) | 3.8 | -0.4 |
| Grenada | 98.73(69.25-135.48) | 142.59(99.81-194.06) | 166.62(117.97-224.24) | 145.34(101.77-196.04) | 0.07(0.06,0.08) | 68.76 | 1.92 |
| Guam | 280.27(198.46-391.58) | 205.89(145.55-283.58) | 342.51(249.20-465.09) | 201.85(146.46-273.90) | -0.04(-0.05,-0.03) | 22.21 | -1.96 |
| Guatemala | 8204.60(5820.74-11258.32) | 143.20(101.84-194.29) | 21217.94(15127.85-29096.89) | 144.41(101.77-198.00) | 0.03(0.02,0.04) | 158.61 | 0.85 |
| Guinea | 6372.73(4459.11-8769.88) | 144.49(100.02-196.64) | 14181.59(9951.78-19579.14) | 143.81(101.76-196.72) | -0.01(-0.02,0.01) | 122.54 | -0.47 |
| Guinea-Bissau | 969.03(672.93-1339.21) | 139.57(97.14-188.05) | 2227.61(1561.93-3076.30) | 140.13(98.65-189.85) | -0.00(-0.01,0.01) | 129.88 | 0.4 |
| Guyana | 868.41(607.02-1223.74) | 133.69(94.05-182.85) | 1053.85(733.75-1451.98) | 136.12(94.43-186.58) | 0.05(0.04,0.06) | 21.35 | 1.82 |
| Haiti | 6457.80(4382.66-8850.08) | 129.53(88.59-177.47) | 15356.58(11020.51-21143.61) | 127.21(90.21-173.49) | -0.04(-0.05,-0.04) | 137.8 | -1.8 |
| Honduras | 4814.51(3393.26-6656.77) | 146.69(103.49-199.61) | 13542.01(9444.27-18567.19) | 144.79(101.35-195.13) | -0.03(-0.04,-0.02) | 181.28 | -1.29 |
| Hungary | 16288.02(11205.87-22160.92) | 139.44(95.82-190.79) | 16400.12(11657.32-21946.93) | 143.28(101.85-193.81) | 0.09(0.08,0.10) | 0.69 | 2.75 |
| Iceland | 452.96(316.10-621.00) | 172.19(120.04-235.92) | 692.11(472.76-937.18) | 172.59(118.30-236.80) | 0.00(-0.01,0.01) | 52.8 | 0.24 |
| India | 1325494.09(982326.31-1705183.17) | 180.74(133.57-231.61) | 2781658.78(2073729.30-3593445.39) | 187.66(140.05-241.67) | 0.16(0.12,0.19) | 109.86 | 3.83 |
| Indonesia | 294863.18(217975.29-382497.85) | 180.41(133.44-230.69) | 578067.57(429387.99-731016.94) | 186.05(138.80-235.11) | 0.16(0.12,0.19) | 96.05 | 3.13 |
| Iran (Islamic Republic of) | 66785.23(49388.41-86252.07) | 157.90(116.85-202.20) | 158908.81(117439.49-203706.53) | 158.93(118.37-202.63) | 0.03(0.02,0.04) | 137.94 | 0.65 |
| Iraq | 20608.14(14396.19-28125.00) | 153.76(109.08-207.04) | 60598.67(42306.45-82918.26) | 153.35(108.10-209.01) | 0.04(0.03,0.06) | 194.05 | -0.27 |
| Ireland | 7336.50(5308.39-9512.17) | 206.19(149.22-267.34) | 11545.21(8101.69-15453.95) | 205.04(144.29-276.50) | 0.12(0.04,0.21) | 57.37 | -0.56 |
| Israel | 8129.29(5785.02-10849.15) | 174.98(124.30-233.37) | 16483.84(11519.18-22133.21) | 170.95(119.31-230.23) | -0.04(-0.06,-0.02) | 102.77 | -2.3 |
| Italy | 106325.41(78674.16-136008.74) | 160.31(117.86-205.62) | 117386.37(86078.84-149280.09) | 161.14(119.53-206.72) | 0.02(0.02,0.03) | 10.4 | 0.52 |
| Jamaica | 2900.91(2004.59-4022.58) | 146.12(100.49-197.97) | 4524.12(3140.67-6107.40) | 145.29(100.14-196.15) | -0.02(-0.02,-0.01) | 55.96 | -0.57 |
| Japan | 268804.41(198621.94-344319.47) | 183.66(136.65-236.57) | 261724.02(192639.04-329975.50) | 181.14(134.29-232.07) | 0.11(0.01,0.21) | -2.63 | -1.37 |
| Jordan | 4337.77(2936.17-6060.27) | 159.86(113.19-216.04) | 19714.59(13930.23-27659.28) | 158.80(114.16-221.64) | -0.02(-0.03,-0.01) | 354.49 | -0.66 |
| Kazakhstan | 21873.76(15359.24-30241.63) | 137.88(96.31-188.81) | 28334.51(19885.68-38931.65) | 141.15(100.02-193.66) | 0.12(0.09,0.15) | 29.54 | 2.37 |
| Kenya | 21808.19(15966.31-28288.54) | 141.55(104.25-183.22) | 60604.12(44503.66-77973.57) | 142.16(104.68-182.99) | 0.05(0.03,0.07) | 177.9 | 0.43 |
| Kiribati | 103.62(72.86-144.67) | 165.83(117.16-226.18) | 186.22(129.12-250.83) | 164.06(113.87-219.56) | -0.02(-0.03,-0.01) | 79.71 | -1.06 |
| Kuwait | 3086.74(2093.68-4353.23) | 172.81(121.11-237.17) | 10616.11(7273.22-14624.03) | 169.24(116.74-231.15) | -0.08(-0.10,-0.06) | 243.93 | -2.06 |
| Kyrgyzstan | 5114.71(3600.49-7115.18) | 135.35(95.69-186.67) | 8956.61(6235.94-12139.30) | 134.37(94.22-184.15) | -0.00(-0.02,0.02) | 75.11 | -0.72 |
| Lao People's Democratic Republic | 5321.66(3828.56-7564.11) | 167.46(117.89-234.47) | 12964.17(9128.34-17744.94) | 177.15(127.01-239.46) | 0.26(0.22,0.29) | 143.61 | 5.79 |
| Latvia | 3824.67(2664.54-5186.87) | 129.49(90.31-176.12) | 2873.18(2001.72-3933.07) | 132.61(92.86-182.85) | 0.13(0.11,0.14) | -24.88 | 2.41 |
| Lebanon | 4242.40(3011.19-5902.04) | 158.78(112.28-218.31) | 9897.90(6806.67-13571.57) | 158.25(110.24-216.68) | 0.01(-0.00,0.02) | 133.31 | -0.33 |
| Lesotho | 1479.84(1046.71-2016.09) | 129.70(91.22-175.08) | 2196.21(1548.94-3046.73) | 129.04(90.79-175.43) | 0.00(-0.01,0.01) | 48.41 | -0.51 |
| Liberia | 2485.42(1779.42-3409.59) | 138.94(99.35-186.43) | 6241.35(4312.12-8625.55) | 137.28(95.08-185.85) | 0.01(-0.01,0.03) | 151.12 | -1.19 |
| Libya | 5174.62(3566.54-7286.00) | 164.76(114.43-225.47) | 12645.21(8761.99-17305.41) | 156.09(108.45-212.39) | -0.18(-0.20,-0.15) | 144.37 | -5.26 |
| Lithuania | 5184.62(3680.42-7106.74) | 129.62(91.55-177.00) | 4224.47(2994.41-5771.69) | 133.11(93.28-183.80) | 0.11(0.10,0.13) | -18.52 | 2.7 |
| Luxembourg | 784.72(542.66-1052.64) | 171.59(119.73-231.38) | 1392.96(978.06-1877.21) | 173.53(122.91-234.53) | 0.04(0.04,0.05) | 77.51 | 1.13 |
| Madagascar | 11815.30(8074.51-16442.94) | 138.89(95.83-192.00) | 31652.09(22207.89-44353.91) | 138.79(97.28-189.41) | 0.03(0.02,0.04) | 167.89 | -0.07 |
| Malawi | 9078.26(6234.75-12412.86) | 130.90(91.33-179.25) | 19441.31(13523.03-26614.36) | 131.54(93.41-182.20) | 0.07(0.05,0.08) | 114.15 | 0.49 |
| Malaysia | 29498.24(20789.70-42195.53) | 189.04(133.04-266.36) | 68763.41(49314.57-96459.46) | 196.25(141.23-272.62) | 0.17(0.14,0.20) | 133.11 | 3.81 |
| Maldives | 293.50(205.54-411.02) | 188.52(134.05-258.58) | 1352.41(947.34-1900.25) | 200.74(143.69-277.15) | 0.27(0.22,0.31) | 360.79 | 6.48 |
| Mali | 8678.05(6083.35-11870.83) | 141.49(98.35-192.43) | 23381.76(15972.07-32468.82) | 143.82(100.29-196.60) | 0.07(0.06,0.08) | 169.44 | 1.65 |
| Malta | 699.32(480.20-942.47) | 169.72(118.20-229.10) | 912.59(645.33-1226.50) | 171.16(121.21-229.76) | 0.02(0.01,0.03) | 30.5 | 0.85 |
| Marshall Islands | 54.16(37.25-75.36) | 171.93(122.63-232.91) | 95.37(67.40-131.74) | 170.25(120.10-233.71) | -0.03(-0.05,-0.02) | 76.08 | -0.97 |
| Mauritania | 2236.44(1557.38-3104.21) | 149.14(103.90-202.78) | 5108.83(3624.26-7027.52) | 151.10(106.24-203.74) | 0.05(0.04,0.05) | 128.44 | 1.31 |
| Mauritius | 2050.61(1395.95-2859.31) | 186.19(128.45-256.05) | 2856.28(2057.60-3884.31) | 190.69(137.12-262.44) | 0.13(0.10,0.15) | 39.29 | 2.42 |
| Mexico | 107876.46(80611.38-139850.75) | 157.22(116.59-202.94) | 218630.72(162671.43-281592.83) | 157.99(117.62-203.56) | 0.01(-0.00,0.02) | 102.67 | 0.49 |
| Micronesia (Federated States of) | 131.82(93.07-185.15) | 171.17(120.04-234.70) | 171.58(120.67-236.81) | 170.52(119.84-233.15) | -0.01(-0.02,0.00) | 30.16 | -0.38 |
| Monaco | 69.77(47.79-94.21) | 181.96(123.68-247.84) | 83.84(58.64-109.92) | 181.74(127.11-243.38) | -0.02(-0.03,-0.00) | 20.16 | -0.12 |
| Mongolia | 2189.57(1508.67-3047.34) | 132.31(91.58-179.36) | 4667.04(3310.86-6385.41) | 136.66(97.88-187.25) | 0.13(0.11,0.15) | 113.15 | 3.29 |
| Montenegro | 931.27(640.22-1275.65) | 141.67(97.18-193.70) | 1013.58(711.96-1361.27) | 141.57(100.23-193.51) | 0.04(0.03,0.05) | 8.84 | -0.07 |
| Morocco | 32164.11(22449.50-44479.57) | 152.31(108.19-207.92) | 59744.56(41789.40-82108.50) | 151.73(106.42-208.61) | -0.01(-0.03,-0.00) | 85.75 | -0.38 |
| Mozambique | 12249.69(8557.53-16618.48) | 126.32(87.64-171.47) | 28639.19(20178.70-39794.77) | 129.44(90.19-175.42) | 0.14(0.12,0.17) | 133.8 | 2.47 |
| Myanmar | 57713.23(39903.74-79912.95) | 163.24(113.76-222.31) | 100678.02(70931.10-138999.25) | 173.89(122.71-239.61) | 0.31(0.27,0.36) | 74.45 | 6.52 |
| Namibia | 1475.40(1019.41-2046.50) | 141.24(97.49-191.07) | 3198.80(2243.28-4399.53) | 142.34(100.73-194.21) | 0.07(0.05,0.09) | 116.81 | 0.78 |
| Nauru | 15.94(11.05-22.16) | 190.51(133.85-260.17) | 17.81(12.62-24.66) | 179.92(127.86-245.42) | -0.16(-0.25,-0.07) | 11.74 | -5.56 |
| Nepal | 26929.77(18609.26-36641.00) | 178.80(125.22-240.43) | 52664.35(37320.84-72238.79) | 175.31(123.70-240.10) | -0.08(-0.10,-0.06) | 95.56 | -1.95 |
| Netherlands | 42175.12(30858.79-53489.48) | 244.44(179.12-309.94) | 47328.55(34644.39-61356.22) | 233.55(169.99-304.99) | -0.17(-0.22,-0.12) | 12.22 | -4.45 |
| New Zealand | 8712.87(6505.02-11202.93) | 239.65(178.85-307.93) | 13987.73(10515.93-17837.96) | 243.41(181.92-311.89) | 0.04(0.03,0.05) | 60.54 | 1.57 |
| Nicaragua | 4019.04(2839.66-5564.57) | 150.35(106.64-205.33) | 9940.91(7065.04-13710.06) | 149.95(107.50-204.34) | 0.01(-0.00,0.03) | 147.35 | -0.27 |
| Niger | 7534.63(5226.70-10401.33) | 141.45(98.32-191.65) | 22112.02(15588.16-30497.63) | 142.00(101.51-192.70) | 0.02(0.01,0.03) | 193.47 | 0.39 |
| Nigeria | 103175.71(75310.51-133026.28) | 151.94(111.79-196.24) | 270855.31(200286.42-347619.05) | 158.09(117.46-203.47) | 0.19(0.16,0.21) | 162.52 | 4.05 |
| Niue | 3.70(2.64-5.09) | 184.68(130.70-255.17) | 3.29(2.34-4.55) | 185.95(131.65-256.02) | 0.06(0.05,0.07) | -11.18 | 0.69 |
| North Macedonia | 2949.83(2026.37-4085.93) | 141.08(97.08-194.90) | 3831.34(2660.75-5158.06) | 141.42(97.64-191.21) | 0.03(0.02,0.04) | 29.88 | 0.24 |
| Northern Mariana Islands | 103.01(72.21-145.75) | 208.91(146.71-289.65) | 107.72(76.76-147.27) | 199.20(140.50-274.50) | -0.16(-0.18,-0.13) | 4.57 | -4.65 |
| Norway | 8268.79(6133.85-10467.51) | 174.41(129.23-222.02) | 11128.42(8266.64-14058.81) | 175.28(130.43-223.41) | 0.04(0.03,0.05) | 34.58 | 0.5 |
| Oman | 2810.36(1937.06-3896.33) | 164.55(115.57-225.43) | 9503.37(6572.00-12962.06) | 165.36(115.57-223.00) | 0.02(0.01,0.03) | 238.15 | 0.49 |
| Pakistan | 153536.37(114268.58-198832.17) | 188.12(139.32-242.62) | 385932.25(287149.73-501492.87) | 184.26(137.44-238.69) | -0.08(-0.09,-0.07) | 151.36 | -2.05 |
| Palau | 27.76(19.51-38.90) | 186.33(131.77-256.52) | 41.31(28.81-55.73) | 188.14(132.81-257.17) | 0.02(0.00,0.04) | 48.8 | 0.97 |
| Palestine | 2155.39(1516.10-2984.40) | 154.95(109.03-210.02) | 6998.96(4972.96-9733.67) | 153.58(109.15-210.65) | -0.05(-0.07,-0.03) | 224.72 | -0.88 |
| Panama | 3277.71(2287.57-4509.74) | 156.37(109.38-211.38) | 6997.92(4948.54-9573.94) | 159.41(112.62-218.07) | 0.07(0.05,0.08) | 113.5 | 1.94 |
| Papua New Guinea | 5716.79(3971.34-7961.46) | 173.27(121.27-237.99) | 16420.13(11616.49-22717.19) | 175.07(123.35-240.82) | 0.02(0.00,0.04) | 187.23 | 1.04 |
| Paraguay | 4919.55(3413.87-6783.49) | 153.51(107.66-208.75) | 11185.38(7804.52-15415.70) | 153.93(108.31-209.84) | 0.01(-0.00,0.01) | 127.37 | 0.27 |
| Peru | 27284.31(18939.67-37717.07) | 153.57(106.24-207.56) | 59289.56(41803.67-81120.16) | 155.92(109.18-213.29) | 0.08(0.06,0.09) | 117.3 | 1.53 |
| Philippines | 93781.63(69446.17-121585.12) | 180.41(134.03-230.88) | 207176.51(153151.57-265765.43) | 183.73(136.85-234.82) | 0.11(0.08,0.14) | 120.91 | 1.84 |
| Poland | 57764.99(42776.52-74185.80) | 140.17(103.78-179.92) | 67085.75(49305.35-85750.42) | 145.23(108.24-187.34) | 0.12(0.12,0.13) | 16.14 | 3.61 |
| Portugal | 18217.41(12919.12-24760.15) | 163.67(115.93-224.63) | 21444.71(15079.62-27999.88) | 165.37(116.55-220.81) | 0.02(0.00,0.03) | 17.72 | 1.04 |
| Puerto Rico | 5445.54(3854.89-7448.74) | 150.68(106.40-206.59) | 5795.81(4008.85-7718.19) | 151.38(105.49-205.06) | 0.04(0.02,0.05) | 6.43 | 0.47 |
| Qatar | 921.70(623.31-1302.55) | 175.51(123.17-244.39) | 7665.34(5100.38-10896.58) | 176.52(119.92-247.32) | 0.09(0.06,0.11) | 731.65 | 0.58 |
| Republic of Korea | 76585.96(54116.39-105994.51) | 165.19(117.01-227.06) | 111195.27(77616.88-151722.00) | 167.37(114.46-230.94) | 0.03(0.02,0.04) | 45.19 | 1.32 |
| Republic of Moldova | 5857.40(4154.41-8064.22) | 126.43(89.69-173.83) | 5717.94(3997.49-7693.01) | 127.35(88.41-173.14) | 0.03(0.01,0.04) | -2.38 | 0.72 |
| Romania | 34475.04(23792.54-46654.88) | 138.48(94.84-189.41) | 31578.72(22151.80-42708.92) | 142.20(99.78-195.60) | 0.12(0.11,0.14) | -8.4 | 2.68 |
| Russian Federation | 203951.44(150342.67-263148.01) | 121.27(89.50-156.28) | 225240.05(165472.10-287813.53) | 131.29(97.23-168.51) | 0.40(0.32,0.49) | 10.44 | 8.26 |
| Rwanda | 6905.41(4775.76-9616.46) | 136.44(96.89-188.24) | 15725.52(10947.88-21706.81) | 137.90(95.05-185.58) | 0.10(0.07,0.14) | 127.73 | 1.07 |
| Saint Kitts and Nevis | 51.25(35.83-70.50) | 144.70(99.39-198.24) | 107.03(74.40-146.31) | 148.56(104.82-203.50) | 0.08(0.07,0.09) | 108.83 | 2.67 |
| Saint Lucia | 158.26(108.97-220.14) | 143.22(99.79-194.52) | 309.78(217.22-427.05) | 144.95(102.82-201.03) | 0.03(0.02,0.04) | 95.73 | 1.21 |
| Saint Vincent and the Grenadines | 124.49(84.15-173.15) | 141.39(98.14-191.78) | 180.10(123.56-240.92) | 142.69(98.17-193.02) | 0.04(0.03,0.05) | 44.67 | 0.92 |
| Samoa | 227.86(161.25-323.25) | 179.85(129.41-248.83) | 331.97(235.56-462.80) | 180.43(126.93-248.40) | 0.02(0.01,0.03) | 45.69 | 0.32 |
| San Marino | 47.63(32.54-63.73) | 174.98(119.08-234.76) | 68.36(47.93-91.69) | 172.11(118.32-235.76) | -0.05(-0.06,-0.04) | 43.51 | -1.64 |
| Sao Tome and Principe | 123.32(85.71-170.31) | 151.23(102.79-205.61) | 291.72(205.97-398.82) | 151.84(107.65-207.02) | 0.02(0.01,0.03) | 136.55 | 0.41 |
| Saudi Arabia | 21257.03(14541.19-29472.84) | 164.03(114.77-224.60) | 79283.28(54248.15-110828.38) | 163.17(114.14-224.14) | -0.01(-0.03,0.00) | 272.97 | -0.52 |
| Senegal | 7669.90(5464.79-10712.23) | 147.28(104.04-201.63) | 18772.15(13318.37-25894.25) | 147.89(105.59-202.92) | 0.03(0.02,0.04) | 144.75 | 0.42 |
| Serbia | 15138.61(10477.71-20669.83) | 141.33(97.62-194.36) | 14944.82(10537.26-20197.69) | 141.66(99.51-193.55) | 0.04(0.02,0.06) | -1.28 | 0.24 |
| Seychelles | 126.68(89.92-176.78) | 191.00(135.87-263.98) | 235.02(168.24-324.78) | 195.20(140.99-271.30) | 0.11(0.08,0.14) | 85.53 | 2.2 |
| Sierra Leone | 4505.54(3123.46-6190.77) | 142.05(99.99-193.69) | 10079.96(6964.72-14072.67) | 141.44(97.29-191.95) | -0.02(-0.03,0.00) | 123.72 | -0.43 |
| Singapore | 5704.01(3974.64-7835.63) | 163.80(113.84-223.34) | 12604.44(8752.40-17009.16) | 165.87(116.34-224.03) | 0.03(0.02,0.04) | 120.98 | 1.26 |
| Slovakia | 7928.90(5603.04-10927.03) | 141.36(99.89-195.87) | 9563.25(6728.59-13022.21) | 144.59(100.24-197.45) | 0.10(0.09,0.11) | 20.61 | 2.29 |
| Slovenia | 3191.52(2241.18-4389.79) | 143.05(99.80-197.91) | 3594.92(2481.81-4902.47) | 146.68(102.25-200.95) | 0.07(0.06,0.07) | 12.64 | 2.54 |
| Solomon Islands | 406.02(289.42-570.15) | 168.54(117.81-232.01) | 995.10(685.83-1386.43) | 167.08(116.62-230.51) | -0.04(-0.06,-0.02) | 145.09 | -0.86 |
| Somalia | 7093.56(4986.04-9930.45) | 127.27(88.82-173.65) | 18720.10(13178.95-25882.65) | 124.77(88.99-168.39) | -0.05(-0.06,-0.04) | 163.9 | -1.96 |
| South Africa | 45408.96(33236.70-58393.23) | 143.60(106.85-184.97) | 86535.03(64068.04-111295.02) | 141.77(105.17-181.78) | -0.01(-0.03,0.00) | 90.57 | -1.27 |
| South Sudan | 5956.67(4173.21-8424.53) | 141.61(101.14-192.86) | 9741.51(6825.35-13200.84) | 137.38(97.42-183.30) | -0.05(-0.08,-0.02) | 63.54 | -2.98 |
| Spain | 71804.20(54027.82-86113.37) | 169.05(127.53-203.10) | 95958.18(72851.51-116032.62) | 170.29(130.39-207.74) | 0.02(0.01,0.03) | 33.64 | 0.74 |
| Sri Lanka | 29801.64(21029.73-41747.84) | 181.14(128.20-250.82) | 44840.55(31441.64-61027.07) | 186.59(131.49-254.84) | 0.17(0.13,0.20) | 50.46 | 3.01 |
| Sudan | 21714.49(15066.22-30175.29) | 146.02(102.37-197.09) | 54667.74(38506.59-75577.00) | 147.40(105.30-200.56) | 0.03(0.02,0.04) | 151.76 | 0.94 |
| Suriname | 435.08(314.58-564.71) | 126.49(91.16-164.02) | 780.20(571.78-989.13) | 125.54(92.05-159.50) | 0.00(-0.01,0.02) | 79.32 | -0.75 |
| Sweden | 17826.65(13011.31-22461.21) | 177.69(129.13-223.98) | 21412.98(15446.10-27384.01) | 174.70(129.30-225.04) | -0.06(-0.07,-0.05) | 20.12 | -1.68 |
| Switzerland | 14142.41(10092.57-19018.74) | 172.93(123.77-232.72) | 18793.78(12891.89-24928.22) | 172.47(118.52-232.61) | 0.01(-0.00,0.02) | 32.89 | -0.27 |
| Syrian Arab Republic | 13711.66(9373.99-19047.73) | 154.70(108.61-208.11) | 20955.22(14765.94-28669.99) | 150.63(106.88-204.91) | -0.08(-0.10,-0.05) | 52.83 | -2.63 |
| Taiwan (Province of China) | 40368.77(28431.55-55366.24) | 190.27(135.34-257.68) | 60048.35(42674.62-80385.96) | 202.09(143.27-277.90) | 0.13(0.10,0.16) | 48.75 | 6.21 |
| Tajikistan | 5477.82(3828.02-7566.19) | 136.75(96.57-187.96) | 12750.90(8885.27-17687.31) | 134.25(94.11-184.46) | -0.03(-0.06,0.00) | 132.77 | -1.83 |
| Thailand | 101594.89(70168.67-141257.00) | 180.28(125.62-245.94) | 150732.78(106024.43-206304.98) | 187.09(131.99-256.12) | 0.17(0.15,0.20) | 48.37 | 3.78 |
| Timor-Leste | 1009.16(705.47-1419.37) | 158.80(112.08-219.27) | 1878.32(1326.37-2625.15) | 162.49(114.00-222.89) | 0.20(0.15,0.25) | 86.13 | 2.33 |
| Togo | 3536.55(2475.43-4932.76) | 142.72(101.17-197.04) | 10065.69(7055.86-13801.61) | 142.95(99.97-193.28) | 0.01(-0.00,0.02) | 184.62 | 0.16 |
| Tokelau | 2.34(1.67-3.20) | 177.81(127.59-241.92) | 2.43(1.74-3.30) | 181.07(129.80-246.07) | 0.09(0.07,0.10) | 3.94 | 1.83 |
| Tonga | 135.15(94.75-187.73) | 182.81(127.58-250.14) | 166.47(120.82-224.72) | 182.66(131.96-245.66) | 0.00(-0.01,0.01) | 23.18 | -0.08 |
| Trinidad and Tobago | 1280.87(950.06-1641.76) | 116.72(85.06-149.61) | 2406.59(1675.84-3219.17) | 148.18(104.07-200.51) | 0.59(0.37,0.81) | 87.89 | 26.95 |
| Tunisia | 11217.36(7822.67-15478.88) | 158.17(109.82-215.50) | 20791.41(14619.48-28466.80) | 156.68(111.97-216.91) | -0.02(-0.03,-0.01) | 85.35 | -0.94 |
| Turkey | 79587.65(58712.44-103791.54) | 156.82(115.86-203.59) | 147440.79(109037.78-191909.70) | 157.39(116.54-204.67) | 0.01(0.01,0.02) | 85.26 | 0.36 |
| Turkmenistan | 4032.64(2807.32-5582.28) | 137.04(95.88-185.14) | 7397.38(5235.01-9992.77) | 141.23(100.26-190.50) | 0.13(0.10,0.16) | 83.44 | 3.06 |
| Tuvalu | 14.52(10.24-19.93) | 169.63(120.27-233.10) | 20.93(14.77-28.49) | 174.61(121.92-236.23) | 0.11(0.10,0.11) | 44.18 | 2.93 |
| Uganda | 14828.75(10448.35-20470.07) | 130.03(90.01-177.79) | 41092.13(28550.43-57472.94) | 134.98(95.67-185.70) | 0.16(0.14,0.17) | 177.11 | 3.8 |
| Ukraine | 75859.50(55058.49-99109.13) | 129.77(94.14-170.60) | 67672.23(49055.19-86447.66) | 128.25(93.89-166.01) | 0.01(-0.02,0.03) | -10.79 | -1.17 |
| United Arab Emirates | 3690.03(2523.49-5206.00) | 177.21(125.73-242.07) | 25400.79(17134.02-34834.53) | 170.29(118.91-235.62) | -0.12(-0.13,-0.11) | 588.36 | -3.91 |
| United Kingdom | 100550.15(74667.88-126988.92) | 155.91(115.27-198.07) | 107987.20(79458.61-136406.79) | 135.94(100.05-173.08) | -0.34(-0.48,-0.19) | 7.4 | -12.81 |
| United Republic of Tanzania | 24015.83(16681.60-33555.82) | 134.65(95.21-183.80) | 63339.83(44722.86-87448.67) | 138.53(97.36-188.59) | 0.15(0.13,0.16) | 163.74 | 2.89 |
| United States Virgin Islands | 159.89(113.74-220.27) | 149.88(107.17-206.45) | 151.56(108.09-204.52) | 152.23(107.28-210.14) | 0.04(0.02,0.07) | -5.21 | 1.57 |
| United States of America | 660739.04(489983.63-841531.53) | 235.03(173.66-299.91) | 824552.10(617697.50-1040824.39) | 221.40(164.64-281.52) | -0.13(-0.19,-0.07) | 24.79 | -5.8 |
| Uruguay | 5659.75(3876.78-7666.17) | 178.12(122.19-242.43) | 6675.90(4661.19-8966.50) | 177.53(124.90-240.31) | -0.01(-0.03,0.00) | 17.95 | -0.33 |
| Uzbekistan | 22287.39(15565.17-30782.95) | 134.61(94.41-183.29) | 49045.07(34530.72-67184.23) | 136.71(96.23-185.63) | 0.07(0.05,0.09) | 120.06 | 1.56 |
| Vanuatu | 198.35(137.41-272.94) | 171.86(120.89-234.53) | 470.62(331.18-655.96) | 170.61(119.51-235.26) | -0.01(-0.02,-0.00) | 137.26 | -0.73 |
| Venezuela (Bolivarian Republic of) | 24753.46(17141.79-33975.34) | 155.65(107.89-211.37) | 44276.05(30411.23-59979.52) | 152.92(106.22-207.16) | -0.02(-0.04,0.00) | 78.87 | -1.75 |
| Viet Nam | 113959.78(78978.22-155540.29) | 199.16(138.30-270.05) | 233506.45(163605.21-318209.00) | 209.00(148.09-283.33) | 0.21(0.19,0.23) | 104.9 | 4.94 |
| Yemen | 12851.86(8832.62-17801.74) | 145.22(103.15-197.60) | 39429.25(28251.53-54373.39) | 142.58(102.34-195.51) | -0.04(-0.06, -0.02) | 206.8 | -1.82 |
| Zambia | 7160.67(5012.06-9954.46) | 134.99(94.74-183.21) | 20850.11(14809.83-28987.39) | 137.25(97.67-187.61) | 0.12(0.09,0.15) | 191.18 | 1.67 |
| Zimbabwe | 9581.98(6619.88-13328.45) | 135.36(92.41-184.48) | 16711.13(11663.83-23310.41) | 131.21(91.44-183.22) | -0.12(-0.14,-0.09) | 74.4 | -3.07 |

Abbreviations: ASR, age-standardized rate; RC, relative change; EAPC, estimated annual percentage change; SDI, sociodemographic index; UI, uncertainty interval; CI, confidence interval.

**Supplemental Tables S7** The decomposition analysis Changes in incidence, prevalence and DALYs number of Schizophrenia by global and 5 SDI regions, according to population-level determinants from 1990 to 2021

| Sex | Location | Overall  difference | Effect | | | Percent（%） | | |
| --- | --- | --- | --- | --- | --- | --- | --- | --- |
|  |  |  | Aging | Population | Epidemiological changes | Aging（%） | Population（%） | Epidemiological changes（%） |
| Incidence | | | | | | | | |
| Both | Global | 350547.42 | -102905 | 463128.1 | -9676 | -29.36 | 132.12 | -2.76 |
|  | High SDI | 51357.79 | 21933.25 | 29491.81 | -67.269 | 42.71 | 57.42 | -0.13 |
|  | High-middle SDI | 70396.62 | 26922.11 | 35528.28 | 7946.231 | 38.24 | 50.47 | 11.29 |
|  | Middle SDI | 152466.19 | -51386.5 | 126690.7 | 77162 | -33.7 | 83.09 | 50.61 |
|  | Low-middle SDI | 94851.12 | -36115 | 130009.6 | 956.487 | -38.08 | 137.07 | 1.01 |
|  | Low SDI | 113824.94 | -1097.96 | 123958.8 | -9035.93 | -0.96 | 108.9 | -7.94 |
| Male | Global | 187899.3 | -51271.1 | 246015.3 | -6844.86 | -27.29 | 130.93 | -3.64 |
|  | High SDI | 25943.89 | 11142.65 | 15694.27 | -893.024 | 42.95 | 60.49 | -3.44 |
|  | High-middle SDI | 35701.65 | 12760.56 | 18865.72 | 4075.375 | 35.74 | 52.84 | 11.42 |
|  | Middle SDI | 82886.47 | -26778 | 67401.68 | 42262.8 | -32.31 | 81.32 | 50.99 |
|  | Low-middle SDI | 49725.97 | -19930.4 | 70509.59 | -853.25 | -40.08 | 141.8 | -1.72 |
|  | Low SDI | 57027.36 | -319.28 | 62871.24 | -5524.6 | -0.56 | 110.25 | -9.69 |
| Female | Global | 162746.3 | -51154.8 | 216730 | -2828.83 | -31.43 | 133.17 | -1.74 |
|  | High SDI | 25334.68 | 10745.44 | 13775.71 | 813.534 | 42.41 | 54.37 | 3.21 |
|  | High-middle SDI | 34303.35 | 14081.96 | 16643.87 | 3577.521 | 41.05 | 48.52 | 10.43 |
|  | Middle SDI | 69963.96 | -24301.7 | 58948.04 | 35317.61 | -34.73 | 84.25 | 50.48 |
|  | Low-middle SDI | 45178.19 | -16203.8 | 59321.68 | 2060.31 | -35.87 | 131.31 | 4.56 |
|  | Low SDI | 56766.3 | -772.81 | 61082.7 | -3543.59 | -1.36 | 107.6 | -6.24 |
| Prevalence | | | | | | | | |
| Both | Global | 9819028 | -92032.2 | 9675040 | 236020.2 | -0.94 | 98.53 | 2.4 |
|  | High SDI | 850432.9 | -29257 | 926721.1 | -47031.2 | -3.44 | 108.97 | -5.53 |
|  | High-middle SDI | 1061004 | -251100 | 944751.1 | 367352.8 | -23.67 | 89.04 | 34.62 |
|  | Middle SDI | 2374943 | -805415 | 3086005 | 94351.98 | -33.91 | 129.94 | 3.97 |
|  | Low-middle SDI | 3119436 | -280960 | 3310318 | 90078.06 | -9.01 | 106.12 | 2.89 |
|  | Low SDI | 2372223 | 68582.58 | 2303648 | -8.148 | 2.89 | 97.11 | 0 |
| Male | Global | 5132777 | -45269 | 5031418 | 146628.1 | -0.88 | 98.03 | 2.86 |
|  | High SDI | 423544.2 | -6091.75 | 482434.4 | -52798.5 | -1.44 | 113.9 | -12.47 |
|  | High-middle SDI | 558610.8 | -134039 | 499463.8 | 193185.7 | -24 | 89.41 | 34.58 |
|  | Middle SDI | 1269372 | -411972 | 1607449 | 73894.48 | -32.45 | 126.63 | 5.82 |
|  | Low-middle SDI | 1661923 | -145463 | 1787217 | 20168.58 | -8.75 | 107.54 | 1.21 |
|  | Low SDI | 1190320 | 38600.86 | 1167332 | -15613 | 3.24 | 98.07 | -1.31 |
| Female | Global | 4693420 | -36896.7 | 4627570 | 102746.6 | -0.79 | 98.6 | 2.19 |
|  | High SDI | 427710.6 | -23895.8 | 441451 | 10155.48 | -5.59 | 103.21 | 2.37 |
|  | High-middle SDI | 497786.4 | -118115 | 445986.9 | 169914.4 | -23.73 | 89.59 | 34.13 |
|  | Middle SDI | 1116298 | -388463 | 1473203 | 31557.03 | -34.8 | 131.97 | 2.83 |
|  | Low-middle SDI | 1468672 | -132802 | 1517333 | 84141.15 | -9.04 | 103.31 | 5.73 |
|  | Low SDI | 1182153 | 30018.51 | 1136323 | 15811.89 | 2.54 | 96.12 | 1.34 |
| DALYs | | | | | | | | |
| Both | Global | 6166263 | -707620 | 6740194 | 133688.9 | -11.48 | 109.31 | 2.17 |
|  | High SDI | 532195.8 | -8430.14 | 583728.5 | -43102.6 | -1.58 | 109.68 | -8.1 |
|  | High-middle SDI | 691871.1 | -150903 | 601346 | 241428.2 | -21.81 | 86.92 | 34.89 |
|  | Middle SDI | 1471548 | -552434 | 1972115 | 51866.56 | -37.54 | 134.02 | 3.52 |
|  | Low-middle SDI | 1956951 | -202426 | 2093828 | 65548.13 | -10.34 | 106.99 | 3.35 |
|  | Low SDI | 1513475 | 39932.45 | 1462347 | 11195.57 | 2.64 | 96.62 | 0.74 |
| Male | Global | 3281429 | -374913 | 3561287 | 95055.12 | -11.43 | 108.53 | 2.9 |
|  | High SDI | 270645.9 | 1538.779 | 307260.8 | -38153.6 | 0.57 | 113.53 | -14.1 |
|  | High-middle SDI | 367068.1 | -81845.8 | 320785.3 | 128128.5 | -22.3 | 87.39 | 34.91 |
|  | Middle SDI | 797719 | -286111 | 1038757 | 45073.23 | -35.87 | 130.22 | 5.65 |
|  | Low-middle SDI | 1060041 | -107213 | 1146859 | 20394.87 | -10.11 | 108.19 | 1.92 |
|  | Low SDI | 772411.7 | 23192.73 | 752737.9 | -3518.91 | 3 | 97.45 | -0.46 |
| Female | Global | 2891581 | -328143 | 3173191 | 46532.84 | -11.35 | 109.74 | 1.61 |
|  | High SDI | 261556 | -10459.2 | 274955.9 | -2940.7 | -4 | 105.12 | -1.12 |
|  | High-middle SDI | 321974.7 | -69672.8 | 281172.8 | 110474.8 | -21.64 | 87.33 | 34.31 |
|  | Middle SDI | 682272.7 | -262336 | 929301.7 | 15307.19 | -38.45 | 136.21 | 2.24 |
|  | Low-middle SDI | 904999.6 | -93180.1 | 942767.2 | 55412.53 | -10.3 | 104.17 | 6.12 |
|  | Low SDI | 741635.2 | 16850.27 | 709739 | 15045.93 | 2.27 | 95.7 | 2.03 |

**Supplemental Tables S8** The decomposition analysis Changes in incidence, prevalence and DALYs number of Schizophrenia by global and 21 GBD regions, according to population-level determinants from 1990 to 2021

| Location | Overall  difference | Effect | | | Percent（%） | | |
| --- | --- | --- | --- | --- | --- | --- | --- |
|  |  | Aging | Population | Epidemiological changes | Aging（%） | Population（%） | Epidemiological changes（%） |
| Incidence | | | | | | | |
| Andean Latin America | 3367.24 | -1675.17 | 5041.324 | 1.089 | -49.75 | 149.72 | 0.03 |
| Australasia | 3123.07 | -75.259 | 1975.065 | 1223.266 | -2.41 | 63.24 | 39.17 |
| Caribbean | 847.52 | -715.589 | 1748.732 | -185.628 | -84.43 | 206.34 | -21.9 |
| Central Asia | 5160.63 | 729.69 | 4536.444 | -105.5 | 14.14 | 87.9 | -2.04 |
| Central Europe | -415.66 | -637.715 | -1354.38 | 1576.438 | 153.42 | 325.84 | -379.26 |
| Central Latin America | 7481.79 | -8397 | 16007.55 | -128.762 | -112.23 | 213.95 | -1.72 |
| Central Sub-Saharan Africa | 16905.35 | -1042.76 | 18070.41 | -122.298 | -6.17 | 106.89 | -0.72 |
| East Asia | 54351.41 | -32485 | 49199.18 | 37637.27 | -59.77 | 90.52 | 69.25 |
| Eastern Europe | 2.13 | -1036.18 | -2418.32 | 3456.635 | -48647.1 | -113536 | 162283.3 |
| Eastern Sub-Saharan Africa | 40032.32 | -4809.85 | 45723.26 | -881.093 | -12.01 | 114.22 | -2.2 |
| High-income Asia Pacific | 5590.07 | 2140.897 | 1782.585 | 1666.586 | 38.3 | 31.89 | 29.81 |
| High-income North America | 17441.39 | 4021.09 | 14262.54 | -842.243 | 23.05 | 81.77 | -4.83 |
| North Africa and Middle East | 75242.09 | -524.111 | 80388.53 | -4622.33 | -0.7 | 106.84 | -6.14 |
| Oceania | 1939.46 | -161.307 | 1644.221 | 456.548 | -8.32 | 84.78 | 23.54 |
| South Asia | 220702.7 | -31949.5 | 249613.7 | 3038.382 | -14.48 | 113.1 | 1.38 |
| Southeast Asia | 48844.81 | -19680.9 | 45782.03 | 22743.7 | -40.29 | 93.73 | 46.56 |
| Southern Latin America | 1892.79 | -1540.58 | 3432.266 | 1.107 | -81.39 | 181.33 | 0.06 |
| Southern Sub-Saharan Africa | 3249.1 | -1380.16 | 4998.295 | -369.036 | -42.48 | 153.84 | -11.36 |
| Tropical Latin America | 6044.6 | -6839.37 | 12892.53 | -8.561 | -113.15 | 213.29 | -0.14 |
| Western Europe | 5562.33 | -2501.85 | 8415.269 | -351.097 | -44.98 | 151.29 | -6.31 |
| Western Sub-Saharan Africa | 83584.85 | -888.398 | 60540.83 | 23932.41 | -1.06 | 72.43 | 28.63 |
| **Prevalence** | | | | | | | |
| Andean Latin America | 100118.1 | 5401.217 | 91714.81 | 3002.107 | 5.39 | 91.61 | 3 |
| Australasia | 53776.11 | -3207.87 | 58157.5 | -1173.52 | -5.97 | 108.15 | -2.18 |
| Caribbean | 34836.19 | -5372.26 | 40536.22 | -327.776 | -15.42 | 116.36 | -0.94 |
| Central Asia | 77918.31 | 1896.505 | 73201.14 | 2820.666 | 2.43 | 93.95 | 3.62 |
| Central Europe | -31092 | -11941.2 | -29293.5 | 10142.8 | 38.41 | 94.22 | -32.62 |
| Central Latin America | 318793.5 | 18802.86 | 296273 | 3717.654 | 5.9 | 92.94 | 1.17 |
| Central Sub-Saharan Africa | 282789.4 | -9186.4 | 275738.3 | 16237.52 | -3.25 | 97.51 | 5.74 |
| East Asia | 838870.7 | -452323 | 1067029 | 224164.9 | -53.92 | 127.2 | 26.72 |
| Eastern Europe | -38993.2 | -16504.9 | -52919.5 | 30431.11 | 42.33 | 135.71 | -78.04 |
| Eastern Sub-Saharan Africa | 857956.7 | -7651.62 | 854084.7 | 11523.58 | -0.89 | 99.55 | 1.34 |
| High-income Asia Pacific | -6957.09 | -42731.5 | 47185.86 | -11411.4 | 614.22 | -678.24 | 164.03 |
| High-income North America | 332466.6 | -74119.8 | 467601.9 | -61015.5 | -22.29 | 140.65 | -18.35 |
| North Africa and Middle East | 1961153 | 144442.3 | 1779627 | 37083.07 | 7.37 | 90.74 | 1.89 |
| Oceania | 27660.71 | 237.084 | 27218.02 | 205.613 | 0.86 | 98.4 | 0.74 |
| South Asia | 6276080 | 99767.62 | 5941106 | 235206.7 | 1.59 | 94.66 | 3.75 |
| Southeast Asia | 976701.8 | -20873.2 | 913171.2 | 84403.75 | -2.14 | 93.5 | 8.64 |
| Southern Latin America | 72417.58 | -3508.05 | 74858.91 | 1066.718 | -4.84 | 103.37 | 1.47 |
| Southern Sub-Saharan Africa | 90808.4 | -5975.73 | 96026.7 | 757.429 | -6.58 | 105.75 | 0.83 |
| Tropical Latin America | 250184.3 | -12618.6 | 258632.6 | 4170.24 | -5.04 | 103.38 | 1.67 |
| Western Europe | 124856.7 | -46930.9 | 189580.7 | -17793.1 | -37.59 | 151.84 | -14.25 |
| Western Sub-Saharan Africa | 1189779 | 4411.845 | 1162204 | 23162.37 | 0.37 | 97.68 | 1.95 |
| **DALYs** | | | | | | | |
| Andean Latin America | 62636.88 | -4055.23 | 65404.47 | 1287.645 | -6.47 | 104.42 | 2.06 |
| Australasia | 34872.82 | -3475.28 | 38046.48 | 301.63 | -9.97 | 109.1 | 0.86 |
| Caribbean | 21174.56 | -3932.9 | 25835.03 | -727.569 | -18.57 | 122.01 | -3.44 |
| Central Asia | 59035.09 | 5348.617 | 52172.87 | 1513.603 | 9.06 | 88.38 | 2.56 |
| Central Europe | -25398.2 | -12783.7 | -19564.9 | 6950.459 | 50.33 | 77.03 | -27.37 |
| Central Latin America | 184388.1 | -25165.9 | 208149.8 | 1404.17 | -13.65 | 112.89 | 0.76 |
| Central Sub-Saharan Africa | 172035.4 | 3052.514 | 169741.8 | -758.873 | 1.77 | 98.67 | -0.44 |
| East Asia | 420305.2 | -462859 | 731472 | 151692 | -110.12 | 174.03 | 36.09 |
| Eastern Europe | -32496.5 | -17731 | -34785.7 | 20020.19 | 54.56 | 107.04 | -61.61 |
| Eastern Sub-Saharan Africa | 545573.5 | -6715.5 | 541433.7 | 10855.21 | -1.23 | 99.24 | 1.99 |
| High-income Asia Pacific | 2746.6 | -21843.1 | 30863.84 | -6274.19 | -795.28 | 1123.71 | -228.43 |
| High-income North America | 192170.6 | -46075.9 | 292526.4 | -54279.8 | -23.98 | 152.22 | -28.25 |
| North Africa and Middle East | 1244162 | 52098.35 | 1181760 | 10303.53 | 4.19 | 94.98 | 0.83 |
| Oceania | 19250.62 | -622.646 | 19874.4 | -1.138 | -3.23 | 103.24 | -0.01 |
| South Asia | 4117494 | 119785.2 | 3743673 | 254035.9 | 2.91 | 90.92 | 6.17 |
| Southeast Asia | 583101 | -122632 | 644092.5 | 61640.55 | -21.03 | 110.46 | 10.57 |
| Southern Latin America | 44741.09 | -2870.66 | 47554.92 | 56.828 | -6.42 | 106.29 | 0.13 |
| Southern Sub-Saharan Africa | 53734.91 | -4662.37 | 60276.42 | -1879.14 | -8.68 | 112.17 | -3.5 |
| Tropical Latin America | 142799.2 | -32056.5 | 170999.5 | 3856.223 | -22.45 | 119.75 | 2.7 |
| Western Europe | 71188.86 | -41561.2 | 125272.5 | -12522.5 | -58.38 | 175.97 | -17.59 |
| Western Sub-Saharan Africa | 759575.5 | 1816.021 | 737880.4 | 19879.08 | 0.24 | 97.14 | 2.62 |

**Supplemental Tables S9**. Joinpoint Analysis and APC in Incidence for schizophrenia by the SDI and Sex

| **Characteristics** | **Sex** | **K_Joinpoints** | **Joinpoints** | **APC** |
| --- | --- | --- | --- | --- |
| **Global** | Both | 7 | 1990, 1995, 1999, 2005, 2009, 2015, 2018 | -0.08, -0.11, -0.02, 0.05, -0.06, -0.1, 0.02 |
|  | Female | 4 | 1990, 1999, 2008, 2019 | -0.11, 0.02, -0.04, 0.06 |
|  | Male | 6 | 1990, 1995, 1999, 2005, 2010, 2019 | -0.04, -0.11, -0.05, 0.07, -0.1, 0.02 |
| **SDI** | | | | |
| **High SDI** | Both | 7 | 1990, 1995, 2000, 2005, 2010, 2015, 2019 | 0.04, -0.07, 0.22, 0.45, -0.29, -0.56, 0.23 |
|  | Female | 6 | 1990, 2000, 2005, 2010, 2015, 2019 | 0.05, 0.18, 0.34, -0.07, -0.46, 0.41, |
|  | Male | 7 | 1990, 1995, 2000, 2005, 2010, 2013, 2019 | 0.03, -0.18, 0.27, 0.51, -0.41, -0.6, 0.05 |
| **High-middle SDI** | Both | 4 | 1990, 2004, 2010, 2016 | 0.08, 0.3, -0.07, 0.47 |
|  | Female | 4 | 1990, 2001, 2010, 2016 | 0.05, 0.23, -0.13, 0.43 |
|  | Male | 4 | 1990, 2005, 2010, 2016 | 0.08, 0.36, -0.03, 0.48 |
| **Middle** | Both | 3 | 1990, 1998, 2013 | -0.18, -0.12, -0.01 |
|  | Female | 4 | 1990, 1997, 2006, 2011 | -0.26, -0.06, -0.21, -0.03 |
|  | Male | 6 | 1990, 1996, 2005, 2009, 2013, 2018 | -0.12, -0.16, -0.06, -0.1, -0.04, 0.04 |
| **Low-middle** | Both | 5 | 1990, 1995, 2010, 2015, 2019 | -0.05, -0.07, 0.28, -0.09, 0.01 |
|  | Female | 4 | 1990, 1995, 2010, 2015 | -0.01, -0.04, 0.35, 0.01 |
|  | Male | 5 | 1990, 1998, 2010, 2015, 2018 | -0.07, -0.11, 0.22, -0.19, -0.04 |
| **Low middle** | Both | 7 | 1990, 1994, 2001, 2004, 2010, 2015, 2018 | 0.01, -0.03, -0.08, -0.04, 0.08, -0.03, -0.08 |
|  | Female | 3 | 1990, 2010, 2018 | 0, 0.07, -0.1 |
|  | Male | 7 | 1990, 1994, 2001, 2004, 2010, 2015, 2019 | -0.01, -0.06, -0.17, -0.07, 0.09, -0.13, -0.03 |

APC: annual percent change; SDI: sociodemographic index

**Supplemental Tables S10**. Joinpoint Analysis and APC in Prevalence for schizophrenia by the SDI and Sex

| **Characteristics** | **Sex** | **K_Joinpoints** | **Joinpoints** | **APC** |
| --- | --- | --- | --- | --- |
| **Global** | Both | 7 | 1990, 1995, 2000, 2005, 2009, 2014, 2018 | -0.01, -0.07, -0.02, 0.16, 0.08, -0.03, 0.08 |
|  | Female | 4 | 1990, 2005, 2015, 2019 | -0.04, 0.1, 0, 0.12 |
|  | Male | 7 | 1990, 1995, 2000, 2005, 2010, 2014, 2018 | 0.02, -0.08, 0.01, 0.2, 0.03, -0.07, 0.08 |
| **SDI** | | | | |
| **High SDI** | Both | 7 | 1990, 1995, 2000, 2005, 2010, 2015, 2019 | 0.04, -0.12, 0.1, 0.41, -0.26, -0.59, 0.21 |
|  | Female | 6 | 1990, 1995, 2005, 2010, 2015, 2019 | 0.04, -0.04, 0.4, -0.05, -0.44, 0.39 |
|  | Male | 7 | 1990, 1995, 2000, 2005, 2010, 2015, 2019 | 0.04, -0.19, 0.23, 0.4, -0.46-0.73, 0.04 |
| **High-middle SDI** | Both | 4 | 1990, 2005, 2010, 2016 | 0.13, 0.42, 0.06, 0.66 |
|  | Female | 4 | 1990, 2005, 2010, 2016 | 0.12, 0.34, 0.04, 0.66 |
|  | Male | 4 | 1990, 2005, 2010, 2016 | 0.13, 0.5, 0.07, 0.64 |
| **Middle** | Both | 4 | 1990, 2005, 2010, 2018 | -0.06, 0.06, 0.13, 0.04 |
|  | Female | 4 | 1990, 1994, 2010, 2018 | -0.15, -0.04, 0.16, -0.03 |
|  | Male | 4 | 1990, 1994, 2005, 2010 | 0, -0.06, 0.16, 0.1 |
| **Low-middle** | Both | 4 | 1990, 2004, 2010, 2015 | -0.04, -0.01, 0.41, -0.06 |
|  | Female | 3 | 1990, 2010, 2015 | 0, 0.54, -0.02 |
|  | Male | 6 | 1990, 1995, 2005, 2010, 2015, 2018 | -0.02, -0.06, 0, 0.33, -0.14, -0.04 |
| **Low middle** | Both | 6 | 1990, 2000, 2006, 2011, 2014, 2018 | -0.07, -0.01, 0.06, 0.29, 0.03, -0.08 |
|  | Female | 5 | 1990, 2000, 2010, 2014, 2018 | -0.05, 0.06, 0.25, 0.11, -0.09 |
|  | Male | 4 | 1990, 2005, 2010, 2015 | -0.08, 0.01, 0.21, -0.08 |

APC: annual percent change; SDI: sociodemographic index
